# Proprioceptors-enriched neuronal cultures from induced pluripotent stem cells from Friedreich ataxia patients show altered transcriptomic and proteomic profiles, abnormal neurite extension, and impaired electrophysiological properties

**DOI:** 10.1093/braincomms/fcad007

**Published:** 2023-01-18

**Authors:** Chiara Dionisi, Marine Chazalon, Myriam Rai, Céline Keime, Virginie Imbault, David Communi, Hélène Puccio, Serge N Schiffmann, Massimo Pandolfo

**Affiliations:** Laboratory of Experimental Neurology, Université Libre de Bruxelles (ULB), 1070 Brussels, Belgium; Laboratory of Neurophysiology, ULB-Neuroscience Institute (UNI), Université Libre de Bruxelles (ULB), 1070 Brussels, Belgium; Laboratory of Experimental Neurology, Université Libre de Bruxelles (ULB), 1070 Brussels, Belgium; Institut de Génétique et de Biologie Moléculaire et Cellulaire UMR 7104 CNRS-UdS / INSERM U1258, Université de Strasbourg, 67404 Illkirch Cedex, Strasbourg, France; Institut de Recherche Interdisciplinaire en Biologie Humaine et Moléculaire (IRIBHM), Université Libre de Bruxelles (ULB), 1070 Brussels, Belgium; Institut de Recherche Interdisciplinaire en Biologie Humaine et Moléculaire (IRIBHM), Université Libre de Bruxelles (ULB), 1070 Brussels, Belgium; Institut de Génétique et de Biologie Moléculaire et Cellulaire UMR 7104 CNRS-UdS / INSERM U1258, Université de Strasbourg, 67404 Illkirch Cedex, Strasbourg, France; Institut NeuroMyoGene (INMG) UMR5310—INSERM U1217, Faculté de Médecine, Université Claude Bernard—Lyon I, 69008 Lyon, France; Laboratory of Neurophysiology, ULB-Neuroscience Institute (UNI), Université Libre de Bruxelles (ULB), 1070 Brussels, Belgium; Laboratory of Experimental Neurology, Université Libre de Bruxelles (ULB), 1070 Brussels, Belgium; Department of Neurology and Neurosurgery, McGill University, Montreal, Quebec H3A 2B4, Canada

**Keywords:** Friedreich ataxia, proprioceptive neurons, induced-pluripotent stem cells, *in vitro* characterization, isogenic controls

## Abstract

Friedreich ataxia is an autosomal recessive multisystem disorder with prominent neurological manifestations and cardiac involvement. The disease is caused by large GAA expansions in the first intron of the *FXN* gene, encoding the mitochondrial protein frataxin, resulting in downregulation of gene expression and reduced synthesis of frataxin. The selective loss of proprioceptive neurons is a hallmark of Friedreich ataxia, but the cause of the specific vulnerability of these cells is still unknown. We herein perform an *in vitro* characterization of human induced pluripotent stem cell-derived sensory neuronal cultures highly enriched for primary proprioceptive neurons. We employ neurons differentiated from healthy donors, Friedreich ataxia patients and Friedreich ataxia sibling isogenic control lines. The analysis of the transcriptomic and proteomic profile suggests an impairment of cytoskeleton organization at the growth cone, neurite extension and, at later stages of maturation, synaptic plasticity. Alterations in the spiking profile of tonic neurons are also observed at the electrophysiological analysis of mature neurons. Despite the reversal of the repressive epigenetic state at the *FXN* locus and the restoration of *FXN* expression, isogenic control neurons retain many features of Friedreich ataxia neurons. Our study suggests the existence of abnormalities affecting proprioceptors in Friedreich ataxia, particularly their ability to extend towards their targets and transmit proper synaptic signals. It also highlights the need for further investigations to better understand the mechanistic link between *FXN* silencing and proprioceptive degeneration in Friedreich ataxia.

## Teaser

Dionisi, Chazalon *et al.*, report the identification of defects in axonal extension and synaptic transmission in sensory neurons in Friedreich ataxia. Pathological features are not fully reverted after removal of the GAA expansion mutation. Further investigations are needed to clarify the effects of FXN silencing in proprioceptive neurons.

## Graphical Abstract

**Graphical Abstract:**
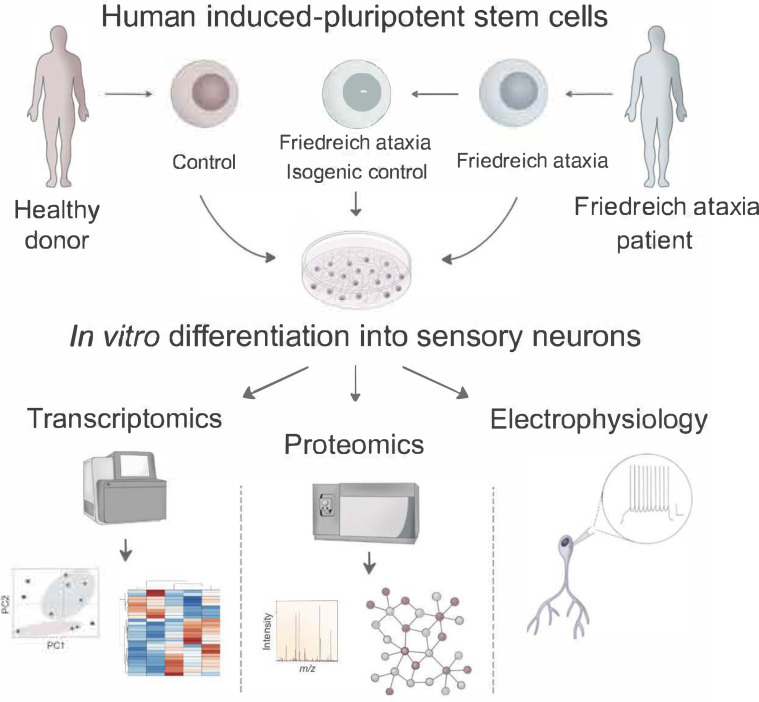

## Introduction

FRDA is the most common autosomal recessive inherited ataxia in Caucasians, accounting for half of the inherited degenerative ataxias and for three-quarters of those with onset before age 25. The disease is caused by a GAA expansion mutation in the first intron of the *FXN* gene on chromosome 9q21.11.^1^ The mutation causes frataxin deficiency by promoting chromatin condensation and disrupting gene transcription.^2^ Frataxin is thought to be a component of the protein complex that assembles Fe-S clusters in the mitochondrial matrix.^3^ Thus, mitochondrial dysfunction and ROS production are proposed to be key features of the pathophysiology of FRDA.^4^ Symptoms include progressive afferent and cerebellar ataxia, dysarthria, pyramidal weakness, skeletal abnormalities (scoliosis and *pes cavus*), cardiomyopathy, insulin resistance and beta cell dysfunction.^5,6^ FRDA is characterized by severe loss of PPNs of the DRGs,^7^ key components of the sensory system that provide information about body position in space and movement.^8^ PPNs pathology is early event in FRDA, already present before symptoms onset. Both a developmental deficit and a progressive degeneration are thought to occur, but the relative contribution of these two components is still unknown, as is the cause of the specific vulnerability of these neurons in FRDA. Indeed, frataxin is a widely expressed protein and the reason for the involvement of a limited set of cells and organs in FRDA is still an open question. Several animal models of the disease have been developed so far, allowing a better understanding of the disease pathophysiology and enabling pre-clinical testing of potential therapeutics.^9–12^ However, the expanded GAA repeat causing FRDA originated from the poly-A segment connecting the two halves of a primate-specific Alu element, so the repeat is not present in the genome of other species. Together with the lethality of systemic *Fxn* KO, this has been a constrain in the generation of mouse models of FRDA. Some of the current models are conditional KOs, which have no GAA repeat expansion and have complete loss of frataxin in targeted tissues, which undergo relentless degeneration and cell death. In other models, expanded GAA repeat have been inserted into the *Fxn* gene or are present in large transgenes containing the human *FXN* locus on a mouse *Fxn*^−/−^ background. Despite low levels of frataxin, the GAA-carrying models show mild or no phenotype, possibly because mice can tolerate much lower levels of the protein than humans. Limitations of mouse models make cell models derived from FRDA patients even more relevant for the study of FRDA pathogenesis. While easily accessible cells as PBMCs and skin fibroblasts have provided some valuable information, these cell types are not affected in the disease, indicating that they can tolerate low frataxin levels without triggering pathological cascades leading to cell dysfunction and death. Conversely, directly obtaining from patients specifically vulnerable cells, as neurons and cardiomyocytes, is nearly impossible. This limitation can be circumvented with the use of hiPSCs. Not only hiPSCs have the potential to generate every cell type of the human body, their differentiation process can at least partially model cellular development. Moreover, the recent development of FRDA sibling ISO CT lines generated by removal of the GAA expansion mutation by means of genome editing approaches, has enabled the direct assessment of the effects induced by the presence or absence of frataxin expression in genetically matched cell lines,^13,14^ reducing the variability due to the use of hiPSCs with different genetic backgrounds.

So far, most studies on hiPSCs-derived neurons in FRDA have utilized differentiation protocols predominantly generating neurons with an immature dorsal cortical phenotype, which do not model specifically vulnerable cells in the disease. Only recently, with the development of a few protocols for the generation of mixed populations of DRG neurons from hiPSCs, a few studies about the possible alterations existing in FRDA sensory neurons have started to emerge.^15,16^

We previously developed a protocol for the rapid differentiation of PPN-enriched cultures from hiPSCs.^17^ In this study, we exploited that system for the in-depth characterization of sensory neurons derived from healthy donors (CT) and FRDA patients as well as from FRDA sibling ISO CTs.^13–15^ We started from the epigenetic analysis of the *FXN* locus and with the investigation of the transcriptomic and proteomic profile of developing or fully differentiated neurons. The analysis was implemented with the characterization of the morphological features of neuronal cultures and with the assessment of the electrophysiological properties of mature neurons. The inclusion of the ISO CT lines helped us to evaluate the possible effects induced by the specific removal of the GAA expansion mutation responsible for the disease. Our study provides new and more specific insights into the pathological features of PPNs in FRDA and highlights the need for a further investigation of the role of GAA expansion mutation and frataxin deficiency in PPNs degeneration in FRDA.

## Materials and methods

### Induced pluripotent stem cell culture and neuronal differentiation

hiPSCs were obtained by reprogramming of human fibroblasts or PBMCs from two healthy donors (*CT*: HEL46.11, male; HEL24.3, male) and five FRDA patients (*FRDA*). The following FRDA lines were used: HEL135.2 (male; 980/1180 GAA repeats); ULBi004FA4 (female; 500/750 GAA repeats); ULBi005FA1 (male; 879/1080 GAA repeats); 4259.11 (male; 550/830 GAA repeats); 4676.2 (male; 700/700 GAA repeats). Three Isogenic Controls (*ISO CT*: E35, 4259.11, 4676.2) were also included in the study. The ISO CT lines 4259.3C7 and 4676.2D3 were derived from the FRDA lines 4259.11, 4676.2, respectively, with the direct excision of the GAA expansion mutation by CRISPR-Cas9, targeting a site 334 bp upstream and a site 896 bp downstream of the GAA expansion.^13,14^ The ISO CT line E35 was derived from another FRDA line (line GM03816: female, 223/490 GAA repeats; not included in the study), by homologous recombination with a correction vector plasmid, *FXN*-*HdAV*, containing 19 kb of the healthy human *FXN* gene.^15^ Participating individuals provided written informed consent according to the respective Institutional Review Board or Ethic Committee.

The 10 lines utilized in the study were indicated as follows: *CT1*: HEL46.11; *CT2*: HEL24.2; *FA1*: HEL135.2; *FA2*: ULBi004FA4; *FA3*: ULBi005FA1; *FA4*: 4259.11; *FA5*: 4676.2; *IcFA4*: 4259.3C7; *IcFA5*: 4676.2D3; *IcFAg*: E35.

Human iPSCs were cultured under feeder-free conditions, in Essential 8 Medium (E8, Thermo Fisher Scientific, Cat. N. A1517001) or mTeSR-1 Medium (Stemcell Technologies, Cat. N. 85850) on Matrigel-coated tissue culture plates (Corning, Cat. No. 356231; 0.05 mg/ml Matrigel solution in DMEM/F12 medium). Cells were fed daily and passaged every 3 days using 0.5 mM EDTA.

Neuronal differentiation was performed as previously described.^17^ Briefly, hiPSCs were plated as single cells on Matrigel treated dishes (0.5 mg/ml Matrigel solution in DMEM/F12 Medium) in E8/mTeSR-1 Medium supplemented with 10 μM ROCK Inhibitor (Y-27632 dihydrochloride, Sigma, Cat. Y0503). The day after seeding (0 DIV), the spent medium was replaced with fresh medium without Y-27632, and cells were allowed to proliferate for other 24 hours, reaching a 60–80% confluency. To initiate sensory differentiation (1 DIV), cells were treated with the following factors: 100 nM LDN193189 and 10 μM SB431542 were added from 1 DIV to 5 DIV, 3 μM CHIR99021 was added from 2 DIV to 7 DIV, 10 μM DAPT and 9 μM SU5402 were added from 2 DIV to 8 FIV. From 1 to 8 DIV, cells were fed daily, and medium was gradually switched from E6 Medium (Thermo Fisher, Cat. N. A1516401) to N2-A Medium with a 25% increment every 2 days. N2-A medium consisted of Neurobasal-A Medium (Thermo Fisher, Cat. N. 10888022), supplemented with 1% N2 Supplement (Thermo Fisher, Cat. N. 17502001) and 1% GlutaMAX (Thermo Fisher, Cat. N. 35050061).

Starting on 9 DIV, cells were fed in N2-B medium, consisting of Neurobasal-A medium supplemented with 1% N2 Supplement, 1% B27 Supplement (Thermo Fisher, Cat. N. 17504001), 1% GlutaMAX and 1% MEM Non-Essential Amino Acids (Thermo Fisher, Cat. 11140050). Medium was replaced every 2 days. 40 ng/ml NT3 and 5 ng/ml BDNF were added from 9 DIV until the last day of differentiation (19-21 DIV), while 5 ng/ml Nerve Growth Factor (NGF) and 5 ng/ml Glial-Derived Neurotrophic Factor (GDNF) were added on 9 and 10 DIV. Small molecule inhibitors and neurotrophic factors utilized in the study were obtained from STEMCELL Technologies.

### Chromatin immunoprecipitation

Investigation of the epigenetic marks of H3 at *FXN* locus was performed by ChIP in fully differentiated neurons obtained from nine hiPSC lines (CT 1-2, FRDA 1-5, IcFA4, IcFA5). Assays were carried out using the Abcam ChIP Kit (ab500), following manufacturer’s instructions. Briefly, differentiated neurons at 20 DIV were fixed with 1.1% formaldehyde and chromatin from lysed nuclei was sheared for 10 min using a 30 sec ON—30 sec OFF sonication cycle in Bioruptor (Diagenode), keeping a temperature of +4°C. Sheared chromatin was immunoprecipitated with antibodies specific for each marker, keeping the samples in incubation overnight at +4°C, in constant rotation. Antibody-antigen complexes were recovered with unblocked Protein A Sepharose beads for 60 min at +4 °C. Finally, quantitative PCR (qPCR) was performed on the eluted DNA using multiple primer pairs to amplify distinct regions within the 5′ end of the *FXN* gene. qPCR was carried out on a QuantStudio3 Real-Time PCR System (Thermo Fisher) using Power SYBR Green Master Mix (Thermo Fisher, Cat. No. 4367659). Primer sets utilized were obtained from Chan *et al.*^18^ and are listed in Supplementary Table 1.

Antibodies used in the assay included: Histone H3 (2 μl/10^6^ cells; Abcam, ab1791); Histone H3 Acetyl K9 (2 μl/10^6^ cells; Abcam, Ab4441); Histone H3 Trimethyl K9 (2 μl/10^6^ cells; Abcam, ab8898); Histone H3 Acetyl K27 (2 μl/10^6^ cells; Abcam, ab4729); Histone H3 Trimethyl K27 (5 μl/10^6^ cells; Abcam, ab6002). 10^6^ cells were used for each marker.

Signals from the immunoprecipitated DNA were calculated as percentage of input and normalized to signals from histone H3 antibody. Results were expressed independently for the acetylated and tri-methylated isoforms of H3K9 and H3K27, and then as ratio between the two, for each region of investigation. ChIP experiments were performed in two biological replicates per cell line. Statistical analysis was performed using *GraphPad Prism v9.2.0* software, dividing the samples in three groups: CT, FRDA and ISO CT lines. Statistical analysis of data was performed using a two-way ANOVA followed by Bonferroni’s test for multiple comparisons (*P < 0.05, **P < 0.01, ***P < 0.001).

### Transcriptome analysis

Transcriptomic analysis was performed by bulk RNA-sequencing for all lines available at three different stages of differentiation: iPSCs, developing neurons (9 DIV) and mature neurons (20 DIV), for a total of 30 samples processed. The 10 lines available were organized in three groups: CT (CT1, CT2), FRDA (FRDA1-5) and ISO CT (IcFAg, IcFA4, IcFA5).

For each sample, RNA was extracted and purified with the RNeasy Mini RNA Kit (Qiagen, Cat. N. 74104), following manufacturer’s instructions. RNA quality was examined on NanoDrop ND-1000 Spectrophotometer (Isogen) and only samples with 260/280 and 260/230 absorbance ratios of ∼2.0 were utilized for the analysis.

Total RNA was kept at −80°C until use. RNA concentration and quality were evaluated a second time prior to library generation using an Agilent Fragment Analyzer automated CE system (Advanced Analytical Technologies, Inc., UK), following manufacturer’s procedure. RNA libraries were generated starting from 1 μg of total RNA, using TruSeq Stranded mRNA Sample Preparation Kit (Illumina). The generated libraries were sequenced on the Illumina HiSeq4000 system as single-end 50-base reads, following Illumina’s instructions. Reads were preprocessed to remove adapter, polyA and low-quality sequences (Phred quality score below 20). Reads shorter than 40 bases were discarded for further analysis. This process was performed using bowtie v2.2.8 aligner. Reads mapping to rRNA sequences were removed for further analysis.

Reads were mapped onto the hg38 assembly of Homo sapiens genome using STAR v2.5.3a. Read coverage over genes in all samples was computed using geneBodyCoverage from RSeQC v2.6.4. Gene expression quantification was performed for uniquely aligned reads using HTSeq-count v0.6.1p1, with gene annotations from Ensembl release 102 and ‘union’ mode. Only non-ambiguously assigned reads were retained for further analysis. PCA was performed to show the main sources of variance in the analysed data using *R* software v3.3.2.

Differential gene expression analysis was performed using the method proposed by Love *et al.*^19^ and implemented in the Bioconductor Package *DESeq2* v1.16.1. Genes with no *P*-value in the resulting file corresponded to gene with high Cook’s distance and were filtered out. *P*-values were adjusted to multiple testing using the Benjamini and Hochberg method. Genes with no adjusted *P*-value in the resulting file corresponded to gene filtered out in the independent filtering step. Independent filtering based on the mean of normalized counts was performed to filter out genes with little or no chance of showing significant evidence of differential expression, resulting in increased power of detection.

Significantly DEGs were selected using the following thresholds: absolute Log_2_(Fold-Change) > 1 and adjusted *P*-value < 0.1. Data were represented as Normalized Read Counts in the log2 scale, using GraphPad Prism v9.2.0 software. GO enrichment analysis was performed for the biological functions of identified genes using the Panther classification system (Panther v.14.0). *P*-values were log10-transformed, and their sign inverted for plotting. Only enriched pathways with *P*-value < 0.01 were considered significant and represented.

### Mass spectrometry-based proteomic analysis

*Sample collection and preparation.* For proteomic analysis, differentiated neurons from all lines available were collected at 20 DIV, washed twice in cold PBS supplemented with Protease and Phosphatase Inhibitors at 0.1% and spun at 300 g for 3 min. After the last washing, the supernatant was discarded, and cell pellets were stored at −80°C until use. For proteomic sample preparation, cell pellets were extracted using 500 µl chlorhydrate of guanidine 8 M and homogenized in a Bullet Blender (Next Advance) for 5 min, at speed 8, with 100 µl of zirconium beads 1 mm, keeping a constant temperature of +4°C. After centrifugation for 15 min at 16 000 g, at +4°C, the supernatant was collected, and protein concentration was measured with filter paper dye-binding assay. 100 µg of proteins from each sample were incubated for 1 h at +4°C with 25 mM dithiothreitol (DTE), followed by incubation with 71 mM iodoacetamide (IAA) for 1 h at +4°C, in the dark. The solution was diluted two times in H_2_O and proteins were precipitated by adding four volumes of cold acetone. After 1 h of incubation at −20°C, samples were spun for 20 min at 13 000 rpm, at +4°C. Proteins were incubated with 4 µg of Trypsin Mass Spectrometry Grade (Promega) in 25 mM NH_4_HCO_3_, overnight at +37°C. Digestion was stopped by adding formic acid (HCOOH) at a final concentration of 0.2%. Peptides were then purified using Oasis HLB 30 cc (Waters) according to manufacturer’s instructions. Peptides were evaporated at 60°C and resuspended in 10 µl of 0.1% HCOOH in H_2_O. The peptide concentration was determined using Pierce Quantitative Fluorometric Peptide Assay (Thermo Fisher, Cat. No. 23290). All samples were spiked with iRT peptides (Biognosys) at the concentration of 1/10.

*DDA for generation of a cell-specific spectral library*. For the generation of spectral libraries, samples from CT, FRDA and ISO CT lines were pooled and processed with the same protocol. 300 µg of peptides were injected into a column C18 (5 µm, 2.1 × 250 mm^2^; Vydac) at 200 µl/min using a gradient of 5–50% acetonitrile/0.1% TFA. 70 fractions were collected ( fraction/1.5 min) and concatenated in 15 fractions (Fr1–Fr16–Fr31–Fr46–Fr61; Fr2–Fr17–Fr32–Fr47–Fr62; Fr3–Fr18–Fr33–Fr48–Fr63; etc.). Fractions were purified using Oasis HLB 30 cc (Waters) according to manufacturer’s instructions. Peptides were evaporated at 60°C and resuspended in 10 µl of 0.1% HCOOH in H_2_O. Peptide concentration was determined using Pierce Quantitative Fluorometric Peptide Assay. All samples were spiked with iRT peptides (Biognosys) at the concentration of 1/10. 8 µg of peptides of each fraction were injected into a Triple TOF 5600 mass spectrometer (Sciex, Concord, Canada) interfaced to an Eksignet NanoLC Ultra 2D HPLC System (Eksignet, Dublin, CA) using Data-Dependent-Acquisition (DDA). MS1 spectra were collected in the range of 400–1250 m/z for 250 ms. The 20 most intense precursors with charge state 2–4 were selected for fragmentation, and MS2 spectra were collected in the range of 50–2000 m/z for 100 ms; precursor ions were excluded for reselection for 12 s. Combined data were searched using ProteinPilot 4.5 (Sciex) and the Paragon Algorithm (Sciex). Data were searched against the human SwissProt database (Jan 2021).

*DIA (SWATH-MS) and Differential Expression Analysis.* 1 µg of peptides was injected using SWATH-MS acquisition on a Triple TOF 5600 mass spectrometer (Sciex, Concord, Canada) interfaced to an Eksigent NanoLC Ultra 2D HPLC System (Eksignet, Dublin, CA). Peptides were injected on a separation column (Eksigent ChromXP C18, 150 mm, 3 µm, 120A) using a two steps acetonitrile gradient (5–25% ACN/0.1% HCOOH for 98 min, then 25–60% ACN/0.1% HCOOH for 60 min) and were sprayed online in the mass spectrometer. Swath acquisitions were performed using 71 windows of variable effective isolation width to cover a mass range of 400–1250 m/z. SWATH MS2 spectra were collected from 50 to 2000 m/z. The collision energy for each window was determined according to the calculation for a charge 2+ ion centred upon the window with a spread of 15. An accumulation time of 45 ms was used for all fragment-ion scans in high-sensitivity mode and for the survey scans in high-resolution mode acquired at the beginning of each cycle, resulting in a duty cycle of ∼2.3 s. Spectra were aligned using *SWATH 2.0* in the PeakView v2.2 Software (Sciex) against the cell-specific spectral library (generated from the search result, allowing no modifications; 5123 protein entries). iRT peptides (Biognosys) were used for retention time calibration. Data were processed in PeakView using a XIC extraction window of 30 min and XIC width of 30 ppm. Peak areas from peptides with >99% confidence and <1% Global False Discovery Rate (FDR) were extracted using MarkerView v1.2.1 (SCIEX). Normalized peak intensity values for extracted proteins were analysed by Student’s *t*-test. Proteins with an absolute Log_2_(Fold Change) > 1 and *P*-value < 0.01 were considered as differentially expressed in this study. Data were represented as Log_2_(Mean Peak Intensity). Functional interactions of differentially expressed proteins were predicted using the Search Tool for Retrieval of Interacting Genes (STRING-DB) database. Gene ontology (GO) enrichment analysis was performed for the biological functions of identified proteins. FDR values were log10-transformed, and their sign inverted for plotting. Only enriched pathways with adjusted *P*-value (FDR) < 0.01 were considered significant and represented.

### Neurite outgrowth

For the analysis of neurite outgrowth, developing neurons at 9 DIV were replated into sterile cloning cylinders (O.D. 8 × H 8 mm^2^; Merck, Cat. No. CLS31668), located in the middle of Matrigel-coated glass coverslips (22 mm diameter, Cat. No. 631.0159, WDR), into six-well plates. Neurons were seeded at a density of 2500 cells/cylinder and were kept in culture accordingly to the differentiation protocol. The day after seeding, the cylinder was gently removed, allowing the radial extension of neurites by developing neurons. At 20 DIV, neurons were fixed with 3.7% paraformaldehyde in PBS for 10 min, permeabilized with ice cold 0.1% Triton X-100 in PBS and incubated in blocking buffer (5% Normal Donkey Serum; Abcam, Cat. N. ab7475) for 30 min at room temperature. Nuclear bodies and neurites were stained overnight at +4 °C with an anti-tubulin III antibody (1:1000; Abcam, Cat. No. ab18207), followed by incubation with Donkey Anti-Rabbit IgG Alexa Fluor 488 (1:1000, Abcam, Cat. No. ab150073). Images were acquired using ZEISS Axio Zoom.V16 Microscope and processed using Zeiss ZEN 2.6 Blue Microscopy Software.

To quantify axonal growth, we adapted the Sholl method of concentric rings to our cultures. Prior to processing, images were modified with ImageJ v1.5.3 (National Institutes of Health, Bethesda, MD) to eliminate fluorescence background and artefacts. For application of the Sholl analysis, we employed the ShollAnalysis ImageJ plug-in, which counts the number of intersections of neurites as a function of distance from the cell soma or explant. Immunofluorescent images were imported into ImageJ and converted in greyscale, the size scale was set accordingly, and the brightness/contrast threshold was selected manually to remove the thinner and shorter neurites emerging from the clusters. Sholl analysis was performed by selecting the centre of the neuronal body cluster as the centre of outgrowth (start radius = 0 inch) and using a step size of 0.5 inch and an end radius of 8 inch. These parameters were chosen to divide the image into concentric annuli at a radial distance of 1000 μm from each other. Intersections were counted from the border of the neuronal body cluster (set as 0 μm) outwards. Results were inputted into *GraphPad Prism 9.2.0* software and statistical analysis was performed between CT and FRDA groups at each individual distance from the body cluster (two-way ANOVA with Bonferroni’s test for multiple comparisons; *P < 0.05, **P < 0.01, ***P < 0.001).

### Electrophysiological analysis

Individual culture slides from CT (CT1 and 2), FRDA (FRDA 1, 2 and 5) and ISO CT (IcFA4, IcFA5) at 19–21 DIV were treated as described in our previous report.^17^ Neuronal cultures were maintained immersed in a thermoregulated chamber, continuously superfused with an oxygenated artificial CSF (aCSF) containing (in mM): NaCl 127, KCl 2.5, NaH2PO4 1.25, MgCl2 1, NaHCO3 26, D-glucose 10, CaCl2 2, bubbled with 95% O2 and 5% CO2 (pH of 7.3, 300–316 mOsm, rate of 1.5–2 ml/min, temperature of 30°C). Patch clamp experiments were performed in whole cell configuration with a solution containing biocytin 0.5% (Sigma-Aldrich, Cat. B4261) and (in mM): KMeSO4 125, KCl 12, CaCl2 0.022, MgCl2 4, HEPES 10, EGTA 0.1, Na2-phosphocreatine 5, Mg2-ATP 4, Na2-GTP 0.5 (pH of 7.2, 292 mOsm). First, passive properties [capacitance (Cm, pF), membrane resistance (Rm, MΩ), membrane time constant (*τ*, ms)] and series resistances were extracted in voltage-clamp mode, as previously shown.^17^ Then, in current-clamp mode, cell excitability was investigated by setting the resting membrane potential at −60 mV and injecting 1 sec depolarizing steps in 10 pA increments (from 0 to 100 pA). Signals were sampled at 10 kHz with a gain of 2 mV/pA. Firing frequency was calculated as number of automatically detected APs over 1 sec step duration, with voltage threshold set at 0 mV for each depolarizing step. Spikes with a peak amplitude below 0 mV were not included in the estimation of firing frequency and neurons were considered to be in accommodation from that point on. The neuron RMP was derived from the averaged off-line values of potential fluctuations during the entire step at 0 pA of injected current. The liquid injection potential of the solution, equal to 6.6 mV, was not subtracted in the calculation of RMP. For cells showing spontaneous AP firing, the RMP was measured manually by the value of neuron potential at 0.02 sec before the first action potential without any current injection. Series resistance was not compensated during recordings.

Series resistances averaged for each neuron group were not significantly different. For single AP recorded neurons, series resistances were 29.0 ± 2.6 MΩ in CT (*n* = 11), 35.3 ± 2.9 MΩ in FA (*n* = 10) and 31.9 ± 3.2 MΩ in IC neurons (*n* = 5); *P* value = 0.2707. Series resistances for burst neurons were 33.2 ± 4.3 MΩ in CT (*n* = 3), 28.5 ± 3.9 MΩ in FA (*n* = 6) and 32.4 ± 7.6 MΩ in IC neurons (*n* = 3); *P* value = 0.7580. For tonic neurons, series resistances were 33.0 ± 1.5 MΩ in CT (*n* = 22); 36.6 ± 1.5 MΩ in FA (*n* = 26) and 36.4 ± 1.4 MΩ in IC (*n* = 17). *P* value = 0.1849 (one-way ANOVA). If access resistance between the beginning and the end of recording changed more than 25%, neurons were not included in the analysis. Passive properties and neuronal excitability were analysed by IgorPro 6.3 software (WaveMetrics, Portland, USA) using Patcher’sPower Tools, NeuroMatic plugins and Microsoft Excel software. For analysis of passive properties, mean values of neurons from same culture replicates were used (between 1 and 10 neurons per replicate). Data are represented as mean of culture replicates ± SEM. For analysis of active properties of tonic neurons, mean values of neurons with homologous firing patterns from same culture replicates were used (between 1 and 10 neurons per replicate). Data are represented as mean of culture replicates ± SEM. When possible, recorded neurons were identified through biocytin-TrKC co-expression, as previously described.^17^

Series resistances averaged for each neuron group were not significantly different. For single AP recorded neurons, series resistances were 29.0 ± 2.6 MΩ in CT (*n* = 11), 35.3 ± 2.9 MΩ in FA (*n* = 10) and 31.9 ± 3.2 MΩ in IC neurons (*n* = 5); *P* value = 0.2707. Series resistances for burst neurons were 33.2 ± 4.3 MΩ in CT (*n* = 3), 28.5 ± 3.9 MΩ in FA (*n* = 6) and 32.4 ± 7.6 MΩ in IC neurons (*n* = 3); *P* value = 0.7580. For tonic neurons, series resistances were 33.0 ± 1.5 MΩ in CT (*n* = 22); 36.6 ± 1.5 MΩ in FA (*n* = 26) and 36.4 ± 1.4 MΩ in IC (*n* = 17). *P* value = 0.1849 (one-way ANOVA).

If access resistance changed more than 25% between the beginning and the end of the recording, the neuron was discarded. Analyses of passive properties and excitability of recorded neurons were performed with IgorPro 6.3 software (WaveMetrics, Portland, USA) using Patcher’sPower Tools, NeuroMatic plugins and Microsoft Excel software. For analysis of passive properties, mean values of neurons from same culture replicates were used (between 1 and 10 neurons per replicate). Data are represented as mean of culture replicates ± SEM. For analysis of active properties of tonic neurons, mean values of neurons with homologous firing patterns from same culture replicates were used (between 1 and 10 neurons per replicate). Data are represented as mean of culture replicates ± SEM. When possible, identity of recorded neurons was assessed for each group with biocytin-TRKC double immunostaining, as previously described.^17^

### Immunoblotting

Western Blot analysis of frataxin expression was performed on mature neuronal cultures at 20 DIV, in all CT and FRDA lines available, along with IcFA4 and IcFA5. For protein extraction, neurons were harvested, washed twice in PBS followed by centrifugation at 200× g for 3 min, and cell pellet was solubilized in chilled 1 × PTR Extraction Buffer (Abcam, Cat. No. ab193970) in water, supplemented with 1 × Extraction Enhancer Buffer (Abcam, Cat. No. 193971) and protease inhibitors (Roche, cOmplete, Mini Protease Inhibitor Cocktail, Cat. No. 11836153001). Samples were incubated on ice for 1 hour, followed by centrifugation at 18.000×g for 20 min at 4°C. The supernatant was collected and stored at −80°C until use. Protein concentration was determined with Pierce BCA Protein Assay Kit (Thermo Fisher, Cat. N. 23225), following manufacturer’s instructions. Protein separation was performed by SDS-PAGE in 12% polyacrylamide gels. Proteins were denatured in Laemmli buffer (4 × Laemmli Sample Buffer, Bio-Rad, Cat. No. 1610747) for 10 min at 95 °C. 20 μg of total protein were loaded per sample and electrophoresis was performed in Tris-glycine SDS running buffer at 100 mV for 1.5–2 hours. Proteins were then transferred onto 0.45 μm pore-sized nitrocellulose membranes at 100 V for 1 hour at 95 °C, using a wet blotting device. Following transfer, membranes were blocked for 5 min with EveryBlot Blocking Buffer (Bio-Rad, Cat. No. 12010020) and incubated with primary antibodies diluted in TBST/Blocking Buffer (1:1), overnight at 4°C with gentle shaking. Membranes were rinsed three times for 5 min with TBST and incubated with secondary antibodies diluted in TBST/Blocking Buffer for 1 hour at room temperature on a shaking platform in the dark. After two washes in TBST and one wash in TBS of 5 min each, blots were dried at room temperature and wrapped in Whatman filter paper and in aluminium foil. Fluorescent signals were captured using the c600 AzureBiosystems detector and cSeries software. Images were converted in greyscale and band intensity quantified with ImageJ v1.5.3 software. Experiments were performed in duplicate, and data analysed with GraphPad Prism v9.2.0 software (two-way ANOVA with Bonferroni’s test for multiple comparisons; *P < 0.05, **P < 0.01, ***P < 0.001).

The following antibodies were used: Frataxin (1:20; Abcam, Cat. No. ab110328), GAPDH-Loading Control (1:5000; Abcam, Cat. No. ab9485), Goat anti-Mouse IgG (H + L), DyLight 800 4×PEG (1:10 000, Thermo Fisher, Cat. N. SA535521), Goat anti-Rabbit IgG (H + L) and DyLight 680 (1:15 000, Thermo Fisher, Cat. No. 35568). Precision Plus Protein Kaleidoscope Standards (Bio-Rad, Cat. No.1610375) were used as control.

### Statistical analysis

Where not specifically indicated, data were visualized and tested for significance using GraphPad Prism v9.2.0 software. In the comparisons between CT, FRDA and ISO CT groups for Chromatin Immunoprecipitation and Sholl analysis, data were analysed with a two-way ANOVA followed by Bonferroni’s test for multiple comparisons. Statistical analysis of passive properties and resting membrane potential from culture replicates was performed with one-way ANOVA followed by Tukey’s test for multiple comparisons. Statistical analysis of active firing properties of recorded neurons from different culture replicates was performed with two-way ANOVA followed by Tukey’s test for multiple comparisons. Significance is defined as *P < 0.05, **P < 0.01, ***P < 0.001, ****P < 0.0001 for all analyses.

### Data availability

The authors declare that all data supporting the findings of this study are available within the article or in the supplementary data files. The dataset generated in the study and processed data are available from the corresponding author upon reasonable request.

## Results

### Epigenetic analysis of *FXN* locus in differentiated neurons

**Figure 1:**
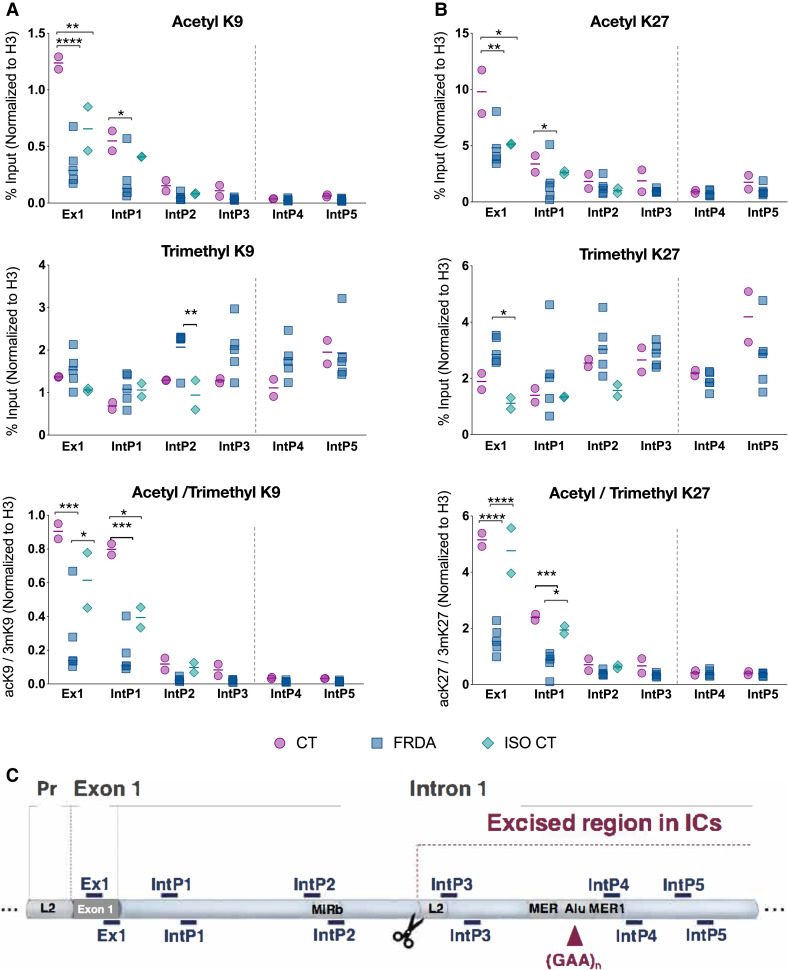
**Epigenomic analysis of *FXN* locus. (A, B)** Investigation of Histone H3 post-translational modifications of *FXN* 5′-end at exon 1 (Ex1), intronic region (intron 1) upstream (IntP1, IntP2, IntP3) and downstream (IntP4, IntP5) of GAA expansion. Chromatin from differentiated neurons was immunoprecipitated using antibodies specific for the Acetylated (ac) and Trimethylated (3m) isoform of Lysine 9 (K9) and Lysine 27 (K27) of H3. Eluted DNA was amplified by qPCR. ChIP-qPCR data were obtained with the percentage of input method and normalized to Histone H3 signals. Results were indicated for each marker, as well as for the ratio between the acetylated and trimethylated isoforms of K9 (A) and K27 (B). ChIP was performed on two independent chromatin preparations for each line included in the study (CT: *n* = 2; FRDA: *n* = 5; ISO CT: *n* = 2). Dots represent the mean of two biological replicates per cell line. Horizontal bars represent mean values for each group of investigation (CT, FRDA and ISO CT lines). For ISO CT group, IcFA4 and IcFA5 were used: in these cell lines, regions immediately upstream and downstream of GAA expansion were removed and no amplification was detected for IntP3, IntP4 and IntP5. Statistical analysis was performed using a two-way ANOVA followed by Bonferroni’s test for multiple comparisons (adjusted *P*-value: *P < 0.05; **P < 0.01; ***P < 0.001; ****P < 0.0001). The vertical broken line shows the location of the GAA repeats. **(C)** Schematic representation of the *FXN* region investigated by ChIP, from the promoter (Pr) to exon 1 and intron 1. Primer sets used for qPCR are indicated as dashes. Putative regulatory non-coding elements (L2, MIRb, MER, MER1, Alu) located in that region of the gene are also represented.

Previous studies showed reduced histone acetylation and increased tri-methylation of H3K9 and H3K27 in the *FXN* locus as hallmarks of FRDA.^20^ We used ChIP to assess these key histone modifications in fully differentiated sensory neuronal cultures (Fig. 1) obtained from nine hiPSC lines (two CTs, five FRDA and the ISO CTs lines IcFA4 and IcFA5), in a region spanning from the first exon of the gene to 880 bp downstream of the GAA expansion^18^ (Fig. 1C). Statistically significant differences between CT and FRDA lines were observed in the level of acetylation of H3K9 and K3K27 in the coding region (exon1) and in the proximal intronic region (Fig. 1A and B). Also, increased trimethylation for H3K9 and H3K27 was observed in FRDA lines, but that was mainly in the regions upstream and downstream of the GAA repeats (Fig. 1A and B, *middle*). ISO CT lines showed a partial recovery of the altered epigenetic marks. The direct comparison of IcFA4 and IcFA5 lines (Supplementary Figure with their sibling FRDA lines (Supplementary Fig. 1) indicated a partial recovery in the level of acetylation of H3K9 and a reduced level of trimethylation of both H3K9 and H3K27 in different regions of the gene. No significant differences were observed instead for H3acK27 between FRDA and ISO CT neurons (Fig.1B, *up*). When we looked at the combined effects of histone acetylation and trimethylation, the recovery of the epigenetic signatures in the isogenic control lines was more evident. Thus, it appears that different mechanisms contribute to gene silencing in FRDA, depending on the distance from the expanded GAA repeats, and that the removal of the GAA repeats can lead to a reversal of the repressive marks in the ISO CTs. In mammals, trimethylation of H3K9 and H3K27 depends on different effectors: H3K9 methylation depends on the histone methyltransferase SUV39H1 and its interaction with the HP1 protein, while H3K27 is methylated by the action of the Polycomb repressive complex 2 (PRC2) and the methyltransferase EZH2.^21^ Looking at the epigenetic profile of *FXN* in our cultures, the action of PRC2 seemed to extend to the entire locus, while the action of SUV39H1 looked limited to the regions immediately upstream and downstream of the GAA repeats. An inhibition of both systems seemed to follow the excision of the GAA expansion mutation in ISO CT neurons.

Of note, increased markers of gene silencing (constant trend of reduced acetylation and increased trimethylation of H3K9 and H3K27) were also observed in CT lines in the regions flanking the GAA repeats (Fig. 1A and B). This agrees with the DNA methylation profile at the *FXN* locus highlighted in different cellular models, including hiPSC-derived sensory neurons:^22,23^ in fact, although a higher rate of DNA methylation was observed in FRDA cells, a sort of physiological increase was observed also in non-affected cells upstream of the GAA repeats.

Overall, our data confirmed the presence of repressive histone marks in FRDA compared to CT sensory neurons along with the possibility to partially revert those marks through the removal of the GAA expansion mutation. This reversal was sufficient to restore the expression of frataxin protein to CT levels (Supplementary Figure of transcriptomic alterations in FRDA and ISO CT lines).

**Figure 2:**
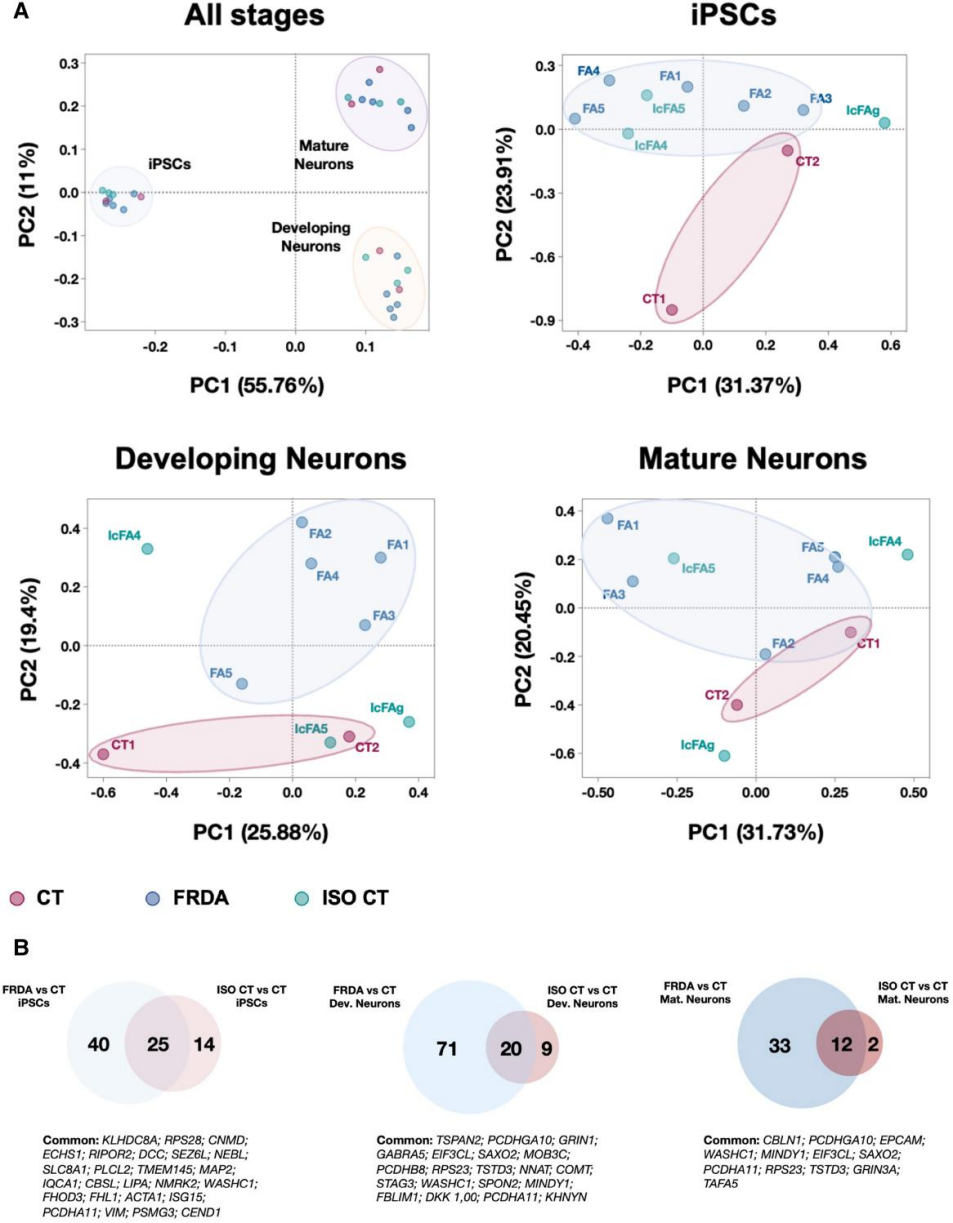
**Transcriptome profiling of FRDA differentiating cultures.** (**A)** Principal Component Analysis (PCA) of transcriptome profile at different stages of differentiations (iPSCs, Developing Neurons and Mature Neurons) for all lines, coloured by cluster membership. **(B)** Venn Diagrams of differentially expressed coding genes in the comparison of FRDA or ISO CT with CT lines, at each stage of differentiation. Common DEGs are indicated under the corresponding diagram (*n* = 2 for CT; *n* = 5 for FRDA; *n* = 3 for ISO CT).

**Figure 3:**
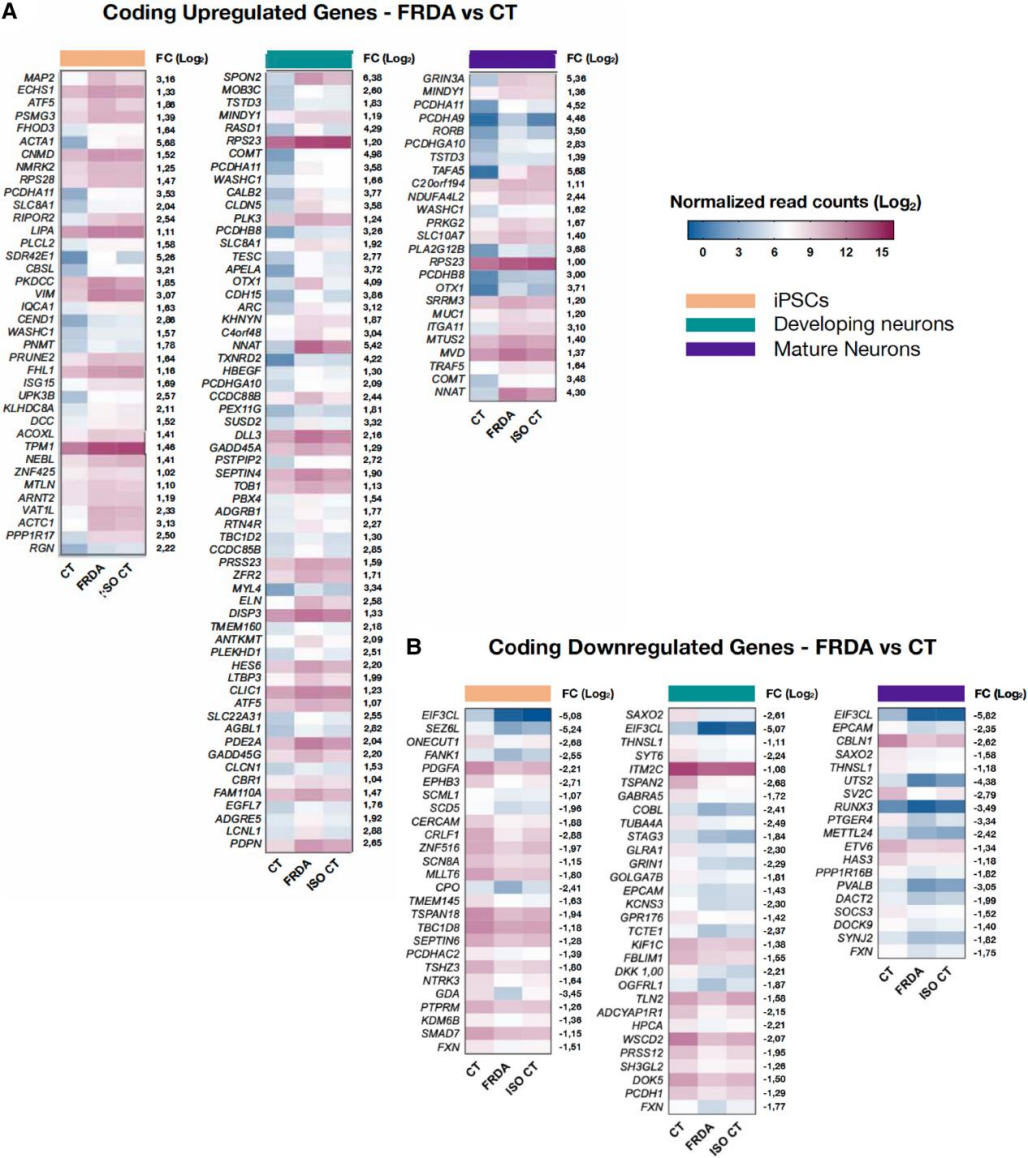
**Heatmaps representing differentially expressed coding genes between FRDA and CT.** [|Log_2_(Fold Change)| > 1; adjusted *P*-value < 0.1] is shown for each stage of differentiation (from left to right: iPSCs; developing neurons; mature neurons). For each gene, the log_2_(Fold Change; FC) between FRDA and CT is shown on the right side of the corresponding heatmap. For each comparison, the transcriptional level of identified DEGs was represented also for the ISO CT group, allowing the evaluation of DEG expression after removal of the GAA expansion mutation. Colour scale represents normalized read count values in the log_2_ scale.

Bulk RNA sequencing was performed for CT, FRDA and ISO CT lines at three different stages of differentiation: iPSCs; developing neurons, prior to complete differentiation supported by treatment with neurotrophic factors, and fully mature neurons (Fig. 2 and Supplementary Figure. All available lines were included in the study (two CT, five FRDA and three ISO CT lines). We separately compared FRDA versus CT, FRDA versus ISO CT and ISO CT versus CT lines. Differentially expressed genes (DEGs) were selected using a cut-off of absolute Log_2_(Fold Change) > 1 and adjusted *P*-value < 0.1. For each comparison, the level of expression of DEGs was then investigated also in the other group, in order to have a complete view of gene expression for all groups. Results were expressed as normalized read counts the in log2 scale (Figs. 2 and 3 and Supplementary Figs. 3–5).

Principal Component Analysis (PCA) revealed well separated clusters for the three stages of differentiation, confirming the ability of all lines to respond similarly to the differentiation protocol. A major difference occurred between iPSCs and the other two stages (PC1 = 55.76%), while developing and mature neuron clusters were closer (PC2 = 11%), suggesting that only minor changes occurred in the final stage of differentiation (Fig. 2A).

PCA performed at each differentiation stage revealed only minor differences between FRDA and CT lines, as indicated by the proximity of the two groups and the low PC values (iPSC: PC1 = 31.37%, PC2 = 23.91%; developing neurons: PC1 = 25.88%, PC2 = 19.4% and mature neurons: PC1 = 31.73%, PC2 = 20.45%). It was not possible to define a clear clustering for the ISO CT lines, with IcFA4 and IcFA5 being unable to significantly diverge from their related FRDA lines (FA4 and FA5, respectively). DEGs were only detected between FRDA and CT or ISO CT and CT lines with no clear reversal of the transcriptional profile between ISO CT and FRDA lines for most identified DEGs (Fig. 2B). Accordingly, ISO CT and FRDA groups shared most DEGs in their comparison to CT lines (Fig. 2B; Supplementary Fig. 3). Overall, the number of identified DEGs was quite low, allowing a deeper assessment of their biological function (Supplementary Table 2).

It is important to mention that we did not use purified PPN cultures. While we previously showed^17^ that our differentiation protocol mostly generates PPNs, it also generates mechanoreceptive neurons (25–30%), which share with PPNs an immediate common progenitor and several markers. It is also likely that our cultures show some minor variability in terms of the specific subtypes of mechanoreceptor and PPN populations known to be present in DRGs. However, by comparing our transcriptomic data with available data from transcriptional profiling of mouse (which may have species-specific differences) and human DRG neurons,^24–28^ we confirmed the presence of PPN markers in finally differentiated cultures without any statistically significant difference between CT, FRDA and ISO CT lines (Supplementary Fig. 3A). A medium-high level of expression for mechanoreceptive-specific markers was also observed, while expression of nociceptive-specific markers was very low (Supplementary Fig. 3B). Thus, we can assume that differences among CT, FRDA and ISO CT cultures were not due to the presence of different proportions of DRG neuronal subtypes. We can also assume that transcriptomics differences in FRDA lines most likely reflect gene expression differences in PPNs, which were the most abundant category of neurons in the differentiated cultures and are the earliest and most severely affected DRG neurons in FRDA.

Downregulation of *FXN* expression was detected in FRDA compared to CT lines at all stages, along with an upregulation of gene expression in ISO CT lines (Fig. 3), in agreement with previous studies,^13,15,16^ and with the reversal of the epigenetic profile and recovery of FXN biosynthesis observed in this study.

Most DEGs were detected in immature neurons, supporting the existence of an important developmental component in FRDA. Some of them persisted in fully mature neurons (Fig. 3). However, we cannot exclude that some of the differences observed for developing neurons were due to the persistence of cell type heterogeneity at this stage when sensory neuronal cultures were probably not yet completely defined.

**Figure 4:**
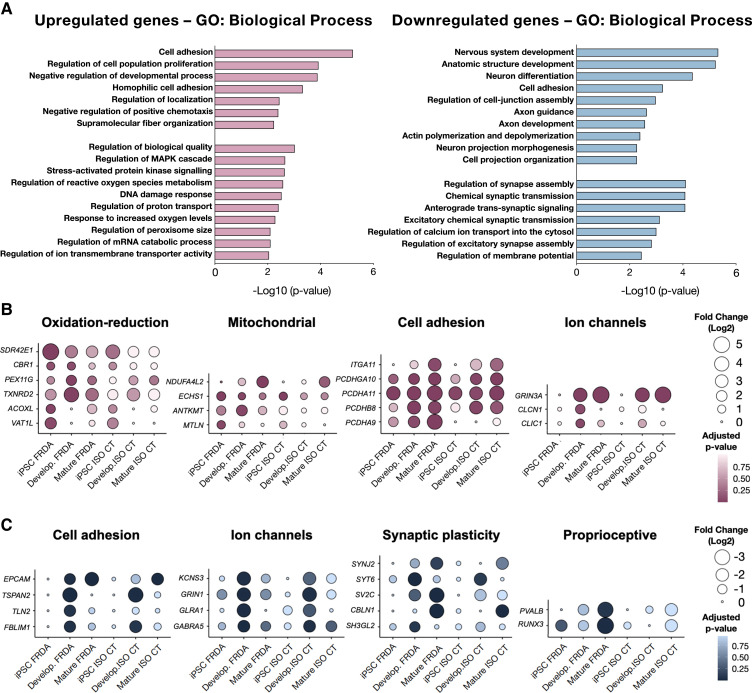
**Biological processes of differentially expressed genes. (A)** Gene ontology analysis for coding upregulated (left) or downregulated genes (right) in FRDA developing and mature neurons in the comparison with CT cells. Only GO terms that were significantly overrepresented (Panther classification system; *P*-value < 0.01) are shown. **(B, C)** Balloon plots for upregulated (B) or downregulated (C) genes in FRDA neurons, classified by their biological functions. The log_2_(Fold change) expression of selected genes is represented for FRDA (*n* = 5) and ISO CT (*n* = 3) lines in their comparison to CTs (*n* = 2) for all stages of differentiation (iPSCs, developing and mature neurons).

Gene ontology analysis for upregulated genes in FRDA developing and mature neurons indicated the involvement of stress activated pathways, MAPK cascade regulation, response to increased oxygen levels, as well as negative regulation of cellular chemotaxis, dysregulation of cell adhesion and activation of catabolic activities (Fig. 4A). We identified specifically involved genes in protein ubiquitination and degradation (*MINDY, AGBL1, KHNYN*),^29,30^ and genes which are known to be upregulated in the presence of peripheral axonal injury (*GADD45A, GADD45G, NNAT, RTN4R*).^31–34^ Interestingly, some of the upregulated genes encoding for adhesion molecules were members of the clustered protocadherin family of homophilic cell-adhesion molecules (*PCDHA9, PCDHA11, PCDHB8, PCDHGA10*) (Fig. 4B and C). They belong to gene clusters which are associated in tandem on chromosome 5, in a unique genomic organization, and their regulated expression is critical for the establishment and function of specific cell-cell connections in the brain and in the spinal cord.^35^

We also detected the upregulation of genes involved in oxidation-reduction processes (*SDR42E1, CBR1, PEX11G, TXNRD2, ACOXL, VAT1L*), although they were not those previously associated with FRDA^36,37^ (Supplementary Table 3) and others encoding mitochondrial proteins (Fig. 4B). The latter included *NDUFA4L2*, which encodes a subunit of NADH dehydrogenase (Complex I); *ANTKMT*, participates in the regulation of mitochondrial ATP synthesis coupled proton transport; *ECHS1*, participating in mitochondrial fatty acid β-oxidation; and *MTLN,* which encodes the protein Mitoregulin, involved in several processes, including fatty acid-beta oxidation and regulation of the mitochondrial membrane potential. However, these changes could not be clearly linked to the expected deficiency in Fe-S proteins in FRDA. We also did not detect any change in the expression level of genes encoding Fe-S proteins or involved in the control of iron homeostasis (Supplementary Table 3).

Downregulated genes in FRDA mainly encoded proteins involved in axon development and guidance, actin polymerization and depolymerization, cell-junction assembly and synaptic plasticity (Fig. 4A). For some of these genes, mostly involved in cytoskeleton organization and axon guidance (*COBL, TUBA4A, KIF1C, TLN2, DOK5, TSPAN2* etc.), differences were already evident and even more statistically significant in developing than in mature neurons. For others, instead, mainly related to synaptic plasticity (*SYNJ2, SV2C, CBLN1, UTS2*, etc.), there was further downregulation in mature neurons (Fig. 2B). We also confirmed^17^ the downregulation of two specific proprioceptive markers, the transcription factor RUNX3 and the Ca^2+^-binding protein parvalbumin (PVALB*)* (Fig. 4C). Of note, RUNX3 is known to be essential for target-specific axon pathfinding of TRKC^+^ DRG proprioceptive neurons.^38,39^ Finally, some of the identified DEGs encoded subunits of ion channel neurotransmitter receptors known to be expressed in proprioceptive neurons. We observed a massive upregulation of *GRIN3A* along with a downregulation of *GRIN1* and *GABRA5* in FRDA neurons. *GRIN3A* and *GRIN1* are involved in glutamate neurotransmission, while *GABRA5* encodes a GABA-A receptor subunit (Fig. 4C). Proprioceptive afferents express these receptors at the presynaptic level.^25,40^ *GRIN3A* and *GRIN1* are both located on chromosome 9 (9q31.1 for *GRIN3A* and 9q34.3, close to telomeric region, for *GRIN1*) and encode distinct subunits of the NMDA family of ionotropic glutamate receptors. A correct balance between different subunits is critical for a proper function of NMDA tetrameric receptors, which are involved in a number of physiological and pathological processes in the nervous system, including synaptic plasticity, refinement of synaptic connections and excitotoxicity:^41^ GluN3A, the subunit encoded by *GRIN3A*, is usually expressed at lower levels compared to GluN1 and GluN2 (encoded by *GRIN1* and *GRIN2*, respectively) and exhibits atypical biophysical properties, such as a reduced permeability to Ca^2+^ and a lower sensitivity to Mg^2+^ block at hyperpolarized potentials.^42^ It is plausible to hypothesize that the concomitant upregulation of *GRIN3A* and downregulation of *GRIN1* at the presynaptic level could be associated with an alteration of the excitatory signalling in affected neurons.

Although our analyses mainly focused on developing and mature neurons, significant alterations were already evident at the iPSC stage (Fig. 2B), for example, for genes that could play important roles in neuron development (*CEND1, DCC, SEZ6L, EPHB3, GDA* etc.), in the regulation of cytoskeletal structures (*MAP2, FHOD3, ACTA1, WASHC1, ACTC1* etc.) and in the cellular response to reactive oxygen species (*SLC8A1, SDR42E1, ACOXL, VAT1L, SCD5* etc.).

For most identified DEGs, we could not observe a clear recovery in ISO CT lines, whose transcriptional profile resembled that of FRDA cells. To confirm that this lack of recovery was not due to intragroup variability for FRDA lines, we also performed a one-to-one comparison between IcFA4 and IcFA5 with their sibling FRDA lines (Supplementary Fig. 4): also in this case, a partial reversal was observed only for a few genes, without any clear association between the presence or absence of the GAA expansion mutation and the transcriptional profile of sibling lines.

Finally, an important finding of our analysis was the detection of numerous aberrantly expressed non-coding RNAs (ncRNAs) (Supplementary Fig. 6). Some of them were either massively upregulated (*RPS4XP22, RCN1P2, H19* etc.) or downregulated (*AP000763.2, AC098847.1, ZBTB8OSP2, RHOXF1-AS1* etc.) in FRDA and ISO CT lines at all stages of differentiation. As these elements participate to gene expression control at the epigenetic, transcriptional and post-transcriptional level, their aberrant expression might impact PPNs development and physiology in FRDA.^43,44^

### Proteome profiling of mature neurons

We performed a quantitative proteomic analysis of mature neurons from all available CT, FRDA and ISO CT lines. As for transcriptomics, we separately compared FRDA versus CT, FRDA versus ISO CT and ISO CT versus CT neurons and then assessed the expression of DEPs also in the third group to have a direct comparison of protein expression. Protein abundance was estimated using the normalized Mean Peak Intensity of peptide spectra, and differentially expressed proteins were selected using a cut-off of absolute Log_2_(Fold Change) > 1 and a *P*-value < 0.01 (Fig. 4).

**Figure 5:**
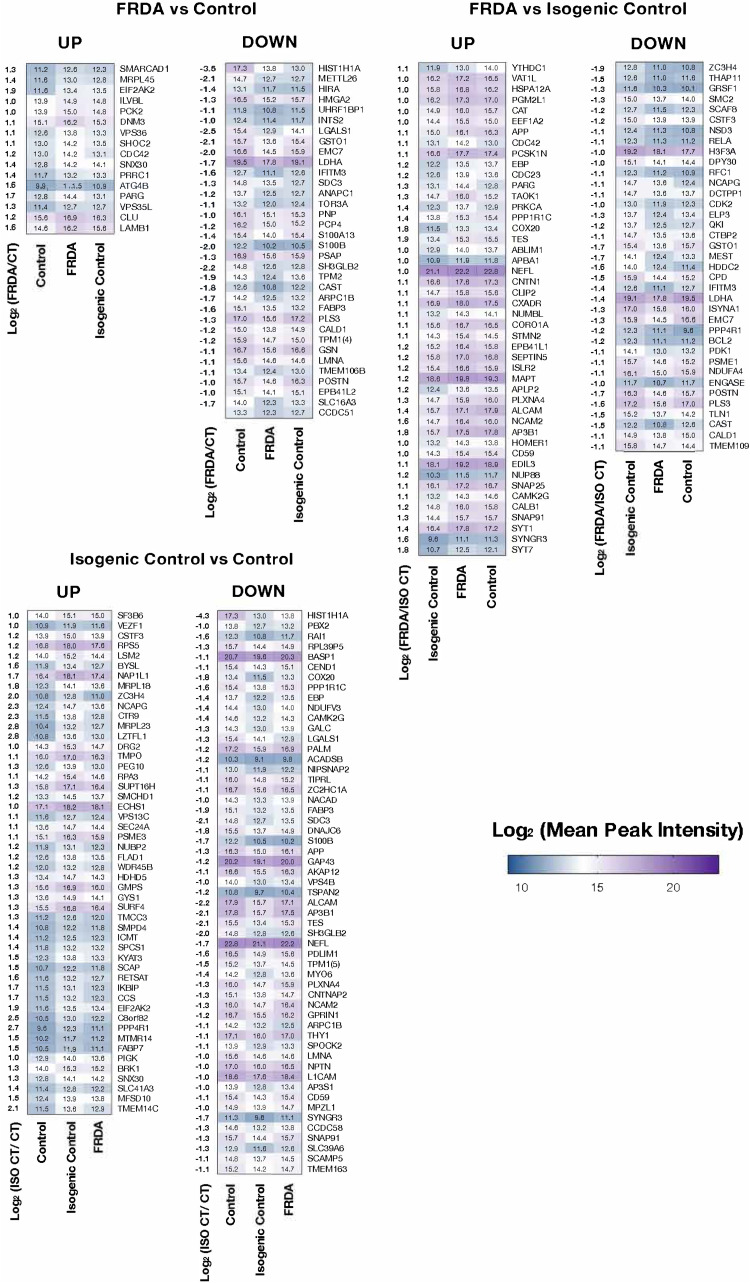
**Heatmaps of differentially expressed proteins in the comparison between FRDA and CT (left), FRDA and ISO CT (middle), ISO CT and CT (right) neurons.** The log_2_(Fold Change) between the two groups analysed (FRDA/CT, FRDA/ISO CT or ISO CT/CT) is shown for each protein on the left side of the corresponding heatmap. For each comparison, the level of expression of identified proteins was represented also for the third group, allowing a direct comparison among all lines. Colour scale represents mean peak intensity of protein spectra in the log_2_ scale. Exact values of mean peak intensities for each protein are indicated in the heatmaps (*n* = 2 for CT, *n* = 5 for FRDA, *n* = 3 for ISO CT).

DEPs were detected in all cases. We obtained 50 proteins that were significantly differentially expressed between FRDA and CT cells, 105 DEPs between ISO CT and CT neurons, and 83 DEPs between FRDA and ISO CTs (Fig. 5, Supplementary Fig. 7).

**Figure 6:**
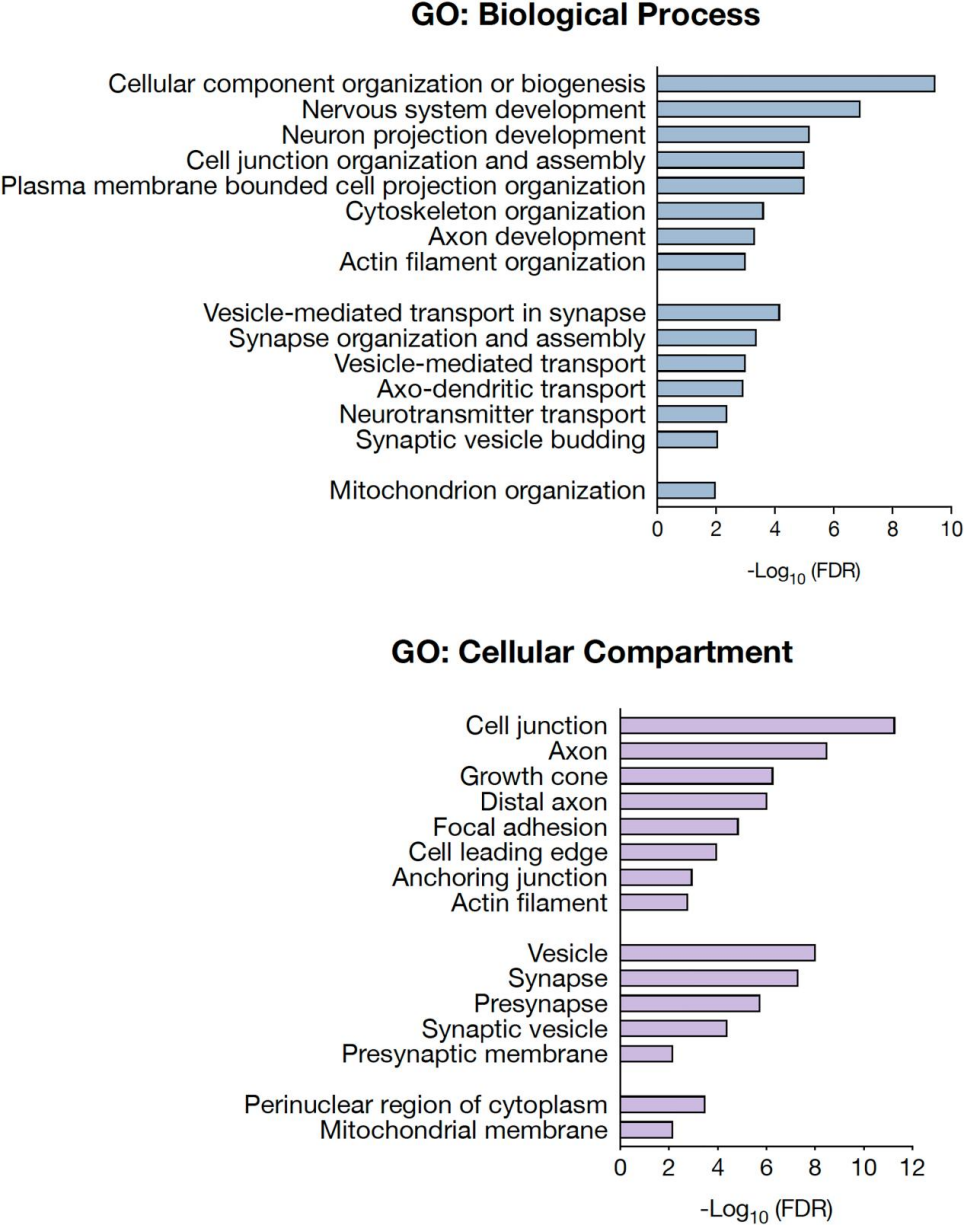
**Biological processes and cellular localization of differentially expressed proteins.** Gene Ontology (GO) analysis of biological processes (left) and cellular compartment (right) for differentially expressed proteins in FRDA and ISO CT neurons. Only GO terms that were significantly overrepresented [False Discovery Rate (FDR) < 0.01] are shown.

Proteomic analysis of differentiated cultures focused on the biological functions and interactions among the detected DEPs. Top enriched pathways were mainly related to neuron projection development, cell junction organization and assembly, and synaptic plasticity (Fig. 6). Accordingly, proteins were mainly localized in the distal axon at the growth cone and at synapses (Fig. 6).

**Figure 7:**
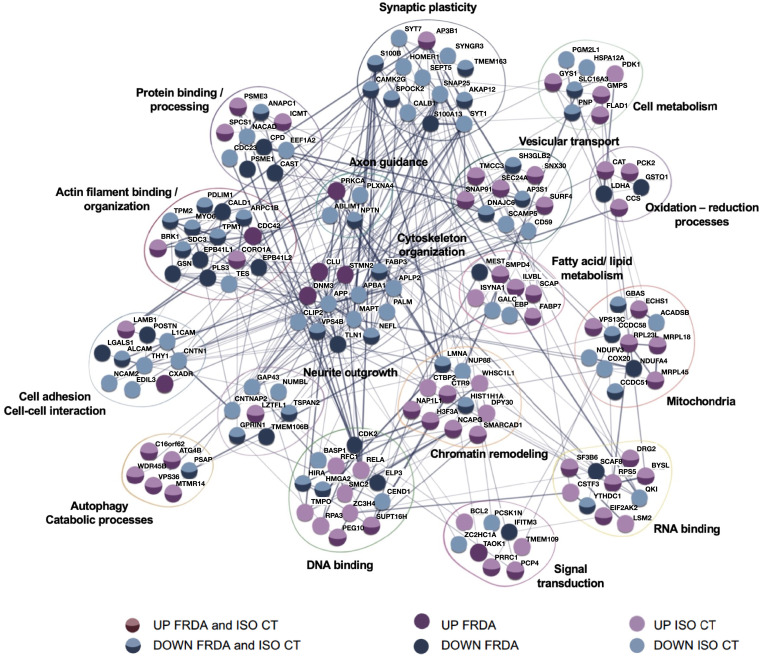
**Protein network of differentially expressed proteins.** Known interactions (STRING-DB) and biological processes of upregulated and downregulated proteins in FRDA and ISO CT neurons are shown.

Most DEPs identified for FRDA and ISO CT neurons are associated in a tight network and take part in the same biological functions (Fig. 7). Though transcriptomics and proteomics data indicate involvement of the same processes and pathways, there is no full correspondence between DEGs and DEPs. This may be due to post-translational modifications and changes in the degradation rate for specific proteins, in addition to the lower sensitivity of proteomics in comparison to RNA-seq.

Proteomic analysis identified proteins involved in cytoskeleton organization and assembly (DNM3, STMN2, NEFL, MAPT, PALM etc.), some of which are specifically related to actin filaments, major components of the growth cone (GSN, ARPC1B, BRK1 etc.), others are associated with cell adhesion (N2CAM, ALCAM, L1CAM, CNTN1 etc.), and axon guidance (ABLIM1, NPTN, PRKCA, PLXNA4) (Figs 4C and 5B). In FRDA and ISO CT, there were lower protein levels of neurofilament light chain (NFL, encoded by NEFL), increasingly used as a biomarker of neuroaxonal damage in many neurological conditions, including FRDA,^45,46^ despite no difference at the transcriptomic level [FRDA versus CT neurons: log_2_(Fold change)= 0.48, adj *P*-value = 0.89; ISO CT versus CT neurons: log_2_(Fold change)= −0.36, adj *P*-value = 0.99], suggesting loss from degenerating axons or post-transcriptional downregulation of this protein.

DEPs involved in vesicular trafficking (SH3GLB2, DNAJC6, SEC24A, TMCC3 etc.), synaptic vesicle recycling (SYT1, SYT7, SYNGR3 etc.) and in synaptic organization and assembly (HOMER1, SNAP25, S100B, etc.) were also detected (Figs 6 and 7).

As for transcriptomics, we also observed a dysregulation of proteins involved in oxidation-reduction processes (CAT, CCS, GSTO1, PCK2, LDHA, COX20), in fatty acid metabolism (ILVBL, SCAP, FABP7, ACADSB etc.) and in autophagy or catabolic processes (VPS36, ATG4B, MTMR14, etc.), localized either in the cytosol or in mitochondria. Other aberrantly expressed mitochondrial proteins included ribosomal proteins (MRPL18, MRPL45, RPL23L), two components of the mitochondrial respiratory chain, NDUFV3 and NDUFA4 of complex I and IV, respectively, and other proteins taking part to the regulation of the mitochondrial matrix volume and mitochondrial transmembrane potential (CCDC51, CCDC58, GBAS/NIPSNAP2, VPS13C).^47,48^ Finally, some of the identified proteins were specifically involved in chromatin remodelling and epigenetic gene control (LMNA, NAP1L1, SMARCAD1, NCAPG, CTR9 etc.), in regulation of transcription and response to DNA damage (HIRA, HMGA2, RFC1, SUPT16H etc.) and in RNA processing (RPS5, EIF2AK2, INTS2, SF3B6 etc.) (Figs 6 and 7). It is important to note that this classification is not exhaustive, as most DEPs are involved in more than one process. A complete list of DEPs with their biological functions is provided in Supplementary Table 4.

Although a significant number of DEPs was identified between FRDA and ISO CT lines, they have known interactions with other DEPs identified between CT and FRDA or ISO CT neurons or both, suggesting that they may be part of a wide homeostatic response to changes originally triggered by the presence of the GAA expansion mutation and persisting in the isogenic controls. Moreover, when we performed a one-to-one comparison of sibling FRDA and ISO CT lines for all identified DEPs, we observed that only a few were differentially expressed between sibling lines (Supplementary Fig. 8).

### Irregular neurite extension in FRDA neurons

**Figure 8:**
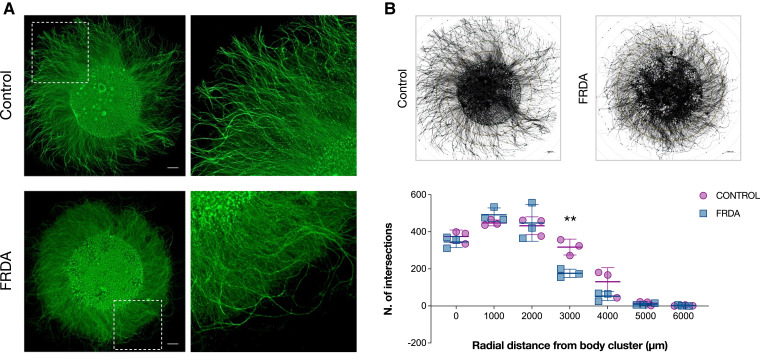
**Morphological analysis of neurite outgrowth. (A)** Representative images of proprioceptive enriched cultures from CT (up) and FRDA (down) lines at 20 DIV labelled with Tubulin β-III. Neuronal cell bodies were isolated in a cluster, allowing radial neurite extension. Scale bar: 1000 μm. **(B)** Schematic representation of morphometric Sholl analysis. Neuron reconstruction and analysis were performed using the *ShollAnalysis* plug-in of *ImageJ* v1.5.3 software (up). The number of intersections between neurites and concentric spheres centred in the neuronal body cluster was determined at various distances, starting from the edge of cluster (0 μm) with 1000 μm increments (down). Each dot in the plot corresponds to a biological replicate (*n* = 3 for CT; *n* = 3 for FRDA). Horizontal bars represent mean values of CT and FRDA biological replicates at each distance from cell cluster (mean ± SD). Statistical analysis was performed with two-way ANOVA followed by Bonferroni’s test for multiple comparisons (adjusted *P*-value: **P < 0.01).

Since a significant fraction of DEGs and DEPs between FRDA and CT neurons was associated with cytoskeletal organization and axon development, we tested if these differences affected neurite extension in our cultures (Fig. 8). We isolated the neuronal bodies of developing neurons in the centre of a plate, allowing the radial extension of their axons in the presence of neurotrophic factors for 12 days. Both FRDA and CT neurons showed the ability to extend long neurites, reaching a maximum external radius of 4000 μm from the edge of soma cluster (Fig. 8A). Direct measurement of single neurite length was hindered by the high density of neurites in culture. However, some irregularities were observed for FRDA neurons, whose longest axons looked tortuous, sometimes with complex twisting. Statistically significant differences between FRDA and CT neurons at the Sholl analysis^49^ were observed at 3000 μm of radial distance from the body cluster (two-way ANOVA with Bonferroni’s test for multiple comparisons; adjusted *P*-value = 0.002), indicating the inability of FRDA neurites to reach the same distances of CTs, probably because of their irregular trajectories (Fig. 8B). These morphological alterations seem to support the existence of deficits in axon development in FRDA neurons.

### Investigation of electrophysiological properties of mature neurons

**Figure 9:**
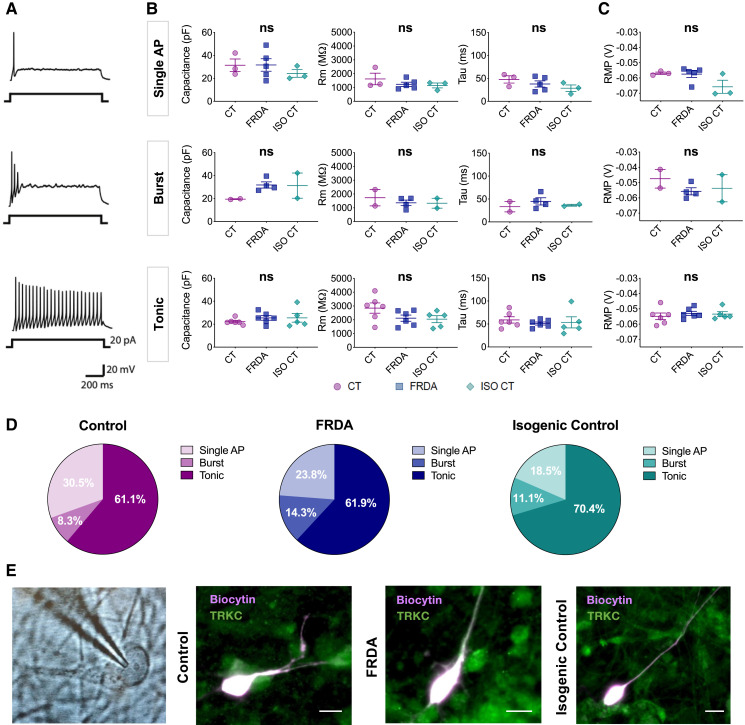
**Electrophysiological properties of differentiated neurons.** Electrophysiological properties of mature differentiated neurons at 19-21 DIV in CT, FRDA, and ISO CT cultures. **(A)** Representative traces of current-clamp recordings at 20 pA of injected current showing three different firing patterns: single action potential (AP) irrespective of the current injected (up), short duration burst of APs (middle) or tonic AP firing (bottom). **(B, C)** Scatter dot plots representing mean values of Capacitance, Membrane Resistance (Rm), Membrane Time Constant (Tau) **(B)** and Resting Membrane Potential (RMP) **(C)** for single AP, burst and tonic neurons in CT, FRDA, and ISO CT culture replicates. Dots represent mean values for neurons from independent culture replicates (between 1 and 10 neurons per replicate). Horizontal bars represent mean values of culture replicates for each group (mean ± SE). No significant differences were observed between the three groups for any of the analysed properties (one-way ANOVA with Tukey’s test for multiple comparisons; ns). **(D)** Percentages of cells showing the three different firing patterns in differentiated CT (left), FRDA (middle) and ISO CT (right) cultures. They were almost equally represented in the three groups (Chi-square test, confidence interval 99%, ns). *n* = 36 for CT, *n* = 42 for FRDA, *n* = 27 for ISO CT. **(E)** Representative image of neuron identified with a 63 × water immersion objective and infrared CCD camera during recordings, followed by representative fluorescent images of biocytin-filled TRKC^+^ neurons in CT, FA and ISO CT cultures (scale bars = 10 μm).

We performed whole-cell patch-clamp recordings to investigate the passive and active electrical properties of differentiated neurons at 19-21 DIV, when they seemed to have already reached a stage of functional maturation, as we previously shown.^17^ Recorded pseudo-unipolar neurons in CT, FRDA and ISO CT cultures were marked with a biocytin-TrKC double immunolabelling (Fig. 9E). TrKC was chosen for its abundant and clear expression in culture and because it is the only common marker which is equally expressed among the different subtypes of proprioceptive neurons. It is important to mention, however, that some TrKB^+^/TrKC^+^ double positive mechanoreceptive neurons might be present in culture, even if they should only represent a small percentage of finally differentiated cultures.

We could detect the three different firing patterns usually observed for DRG neurons, including proprioceptors^25,50–53^ (Fig. 9A), which were almost equally represented in CT, FRDA and ISO CT neurons (Fig. 9D). Some neurons exhibited a rapid adaptation and generated a single action potential (AP) independently of the intensity of injected currents (Fig. 9A, *up*); others displayed a burst of APs followed by accommodation upon sustained stimulation (Fig. 9A, *middle*) and a third type of neuron, the most represented in culture, exhibited a tonic firing pattern, followed by accommodation upon increased current stimulations (Fig. 9A, *bottom*). These different behaviours likely corresponded to rapidly (single AP and burst) and slowly adapting (tonic) neurons, both of which have been detected in muscle spindle and Golgi tendon organs.^25^

The analysis of passive properties for culture replicates of neurons with different firing patterns revealed the absence of significant differences between CT, FRDA and ISO CT neurons (Fig. 9B). Single AP neurons showed a capacitance (Cp) of 31.4 ± 5.5 pF in CT, 31.7 ± 5.4 pF in FRDA, 24.3 ± 3.3 pF in ISO CT (*P* = 0.5967), a membrane resistance (Rm) of 1617.9 ± 419.5 MΩ in CT, 1233.3 ± 153.8 MΩ in FRDA, 1149.2 ± 176.7 MΩ in ISO CT (*P* = 0.4400) and a membrane time constant (Tau, τ) of 47.7 ± 7.9 ms in CT, 37.8 ± 6.8 ms in FRDA, 28.6 ± 7.1 ms in ISO CT (*P* = 0.3051).

For burst neurons, Cp was 19.5 ± 0.4 pF in CT, 31.8 ± 2.7 pF in FRDA, 31.3 ± 11.0 pF in ISO CT (*P* = 0.2758), Rm was 1730.7 ± 591.2 MΩ in CT, 1362.3 ± 199.8 MΩ in FRDA, 1331.6 ± 359.3 MΩ in ISO CT (*P* = 0.7043), τ was 33.2 ± 11.3 ms in CT, 44.9 ± 7.9 ms in FRDA and 36.4 ± 2.1 ms in ISO CT (*P* = 0.6192).

Finally, tonic neurons exhibited a Cp of 22.3 ± 1.1 pF in CT, 25.5 ± 2.0 pF in FRDA, 25.5 ± 3.7 pF in IC (*P* = 0.5342), a Rm of 2849.2 ± 372.5 MΩ in CT, 2119.1 ± 223.8 MΩ in FRDA, 2042.2 ± 254.5 MΩ in ISO CT (*P* = 0.1367), a τ of 58.8 ± 6.9 ms in CT, 51.1 ± 3.3 pF in FRDA and 53.5 ± 12.1 pF in ISO CT (*P* = 0.7561).

Results suggested that the presence of the GAA expansion mutation was not causing any significant alteration of neuronal intrinsic electrical passive properties in our cultures.

The RMP was also evaluated for each firing pattern (Fig. 9C). Also in this case, no significant differences were observed among CT, FRDA and ISO CT culture replicates: single AP neurons showed a RMP of −56.9 ± 0.8 mV in CT, −57.4 ± 2.2 mV in FRDA, −65.7 ± 4.3 mV in ISO CT (*P* = 0.1016); for burst neurons the RMP was of −47.5 ± 6.1 mV in CT, −55.7 ± 2.3 mV in FRDA, −53.7 ± 8.9 mV in IC (*P* = 0.5103) and finally, the RMP for tonic neurons was −54.9 ± 2.2 mV in CT, −53.0 ± 1.3 mV in FRDA and −53.5 ± 1.6 mV in ISO CT (*P* = 0.7173).

**Figure 10:**
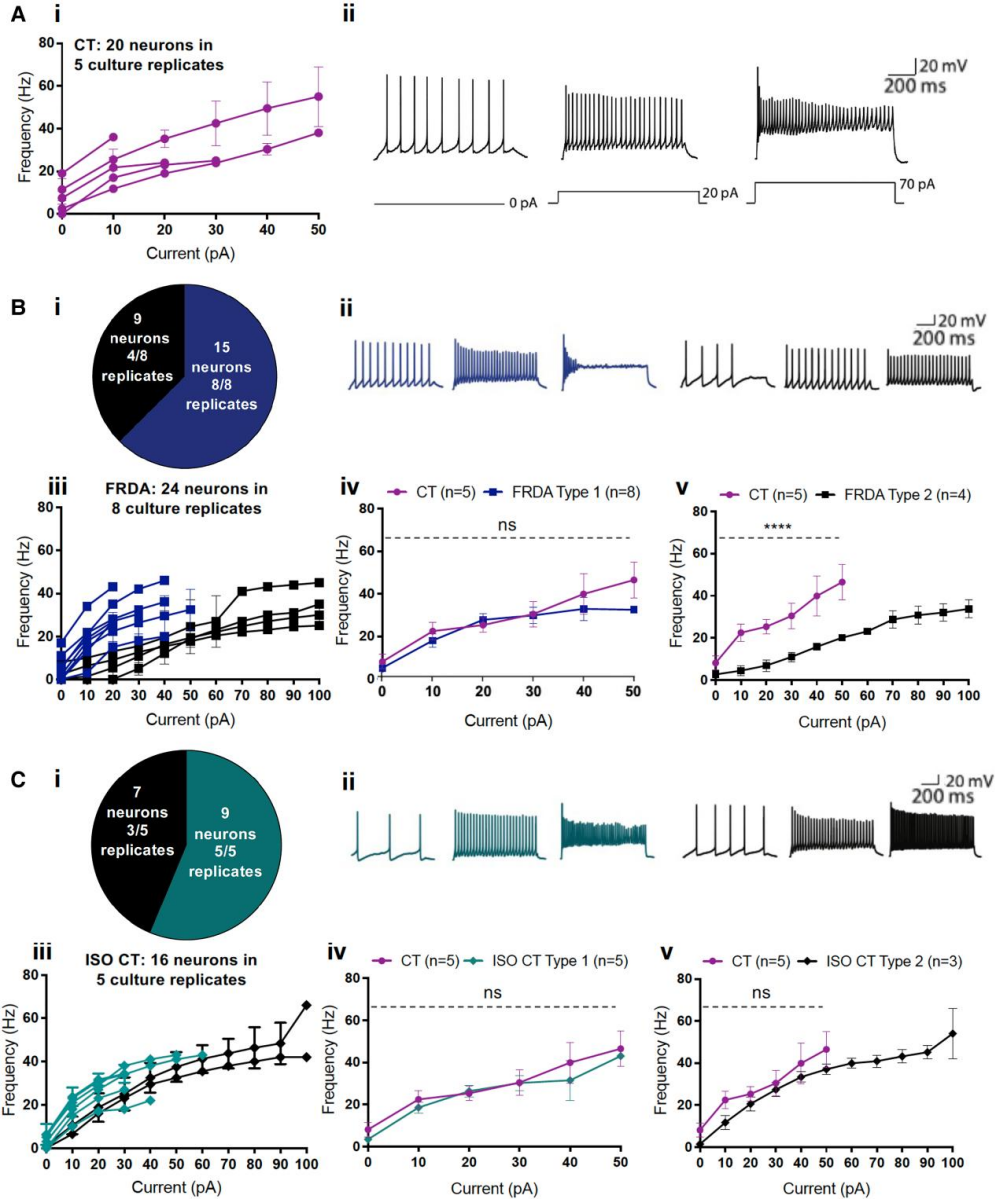
**Firing properties of tonic differentiated neurons.** Firing responsiveness to depolarizing 1 sec current steps in CT, FRDA and ISO CT tonic neurons. **(A) (i)** Current-frequency scatter dot plot with connecting lines for individual culture replicates of CT tonic neurons (magenta) in response to current steps from 0 to 50 pA. Each line corresponds to a single culture replicate (between 1 and 10 neurons per replicate). CT neurons exhibited accommodation starting at 30 to 50 pA of injected currents. **(ii)** Representative traces of current-clamp recordings in CT neurons in response to 0 (left trace), 20 (middle trace) and 70 pA (right trace) of injected current. **(B) (i)** Pie chart indicating the number of FRDA neurons with two different tonic firing patterns and the number of culture replicates in which they were observed (Type 1: rapid accommodation lighter; Type 2: slower accommodation darker) and **(ii)** representative traces of current-clamp recordings for each type (at 0, 20 and 70 pA of injected current). **(iii)** Current-frequency scatter dot plot with connecting lines for individual culture replicates for tonic FRDA neurons of either Type 1 or Type 2 in response to depolarizing current steps. Each dot corresponds to mean ± SEM of replicates for increasing injected currents (between 1 and 3 neurons per replicate). All culture replicates are represented. **(iv)** Comparison of firing frequencies between Type 1 (lighter squares; *n* = 8) and **(v)** Type 2 (darker squares; *n* = 4) FRDA neurons and CT neurons (circles; *n* = 5). Each dot represents the mean firing rate observed at a defined injected current for recorded neurons of the same type from different culture replicates. Type 1 FRDA neurons showed no significant differences in AP frequency compared to CT neurons and accommodation at 30–50 pA. Type 2 FRDA tonic neurons displayed a slower accommodation and a significant reduction of AP frequency compared to CT neurons (two-way ANOVA with Tukey’s test for multiple comparisons; ns for Type 1 neurons; ****P < 0.0001 for Type 2 neurons). Results are expressed as mean of replicates ± SEM for each injected current. **(C) (i)** Pie chart indicating the number of ISO CT neurons with two different tonic firing patterns and the number of culture replicates in which they were observed (Type 1: rapid accommodation; Type 2: slow accommodation) and **(ii)** representative traces of current-clamp recordings for each type. **(iii)** Current-frequency scatter dot plot with connecting lines for culture replicates of tonic ISO CT neurons of either Type 1 or Type 2 in response to depolarizing current steps. Each dot corresponds to mean ± SEM of replicates for increasing injected currents (between 1 and 4 neurons per replicate). All culture replicates are represented. **(iv)** Comparison of firing frequencies between Type 1 (lighter squares; *n* = 5) and **(v)** darker squares Type 2 (; *n* = 3) ISO CT neurons and CT neurons (circles; *n* = 5). Each dot represents the mean firing rate observed at a defined injected current for recorded neurons of the same type from different culture replicates. Type 1 ISO CT tonic neurons showed no significant differences in AP frequency compared to CT neurons and accommodation at 30–50 pA. Type 2 ISO CT tonic neurons showed no significant differences in AP frequency compared to CT neurons (two-way ANOVA with Tukey’s test for multiple comparisons; ns) but displayed a slower accommodation. Results are expressed as mean of replicates ± SEM for each injected current.

We next focused on the characterization of the active firing properties of tonic neurons in CT, FRDA and ISO CT lines (Fig. 10). Those neurons exhibited either a spontaneous activity (13/20 tonic CT, 13/24 tonic FRDA and 7/16 tonic ISO CT neurons) or a low threshold of AP generation [Rheobase = 10 ± 0 pA CT versus 15.45 ± 2.473 pA FRDA versus 10 ± 0 pA ISO CT (Mean ± SEM)], accordingly to what is usually observed for proprioceptive neurons. Most of these neurons started to show accommodation at about 30–50 pA of injected currents.

Nevertheless, there were still some alterations in the spiking profile of tonic FRDA (Fig. 10B) and ISO CT neurons (Fig. 1C) as compared to CT neurons (Fig. 10A). Indeed, in contrast to tonic CT neurons that all accommodated rapidly (between 30 and 50 pA of depolarizing current step injection), two different behaviours were observed in FRDA culture replicates, that we defined as ‘Type 1’ and ‘Type 2’: Type 1 FRDA neurons (15 neurons in 8 replicates) showed no significant differences in AP frequency compared to CT neurons and accommodation at 30–50 pA (Fig. 10Biv). Type 2 FRDA neurons (9 neurons in 4 replicates), instead, showed no accommodation until 100 pA of injected current and a significant reduction of AP frequency compared to CT neurons in response to the same level of injected currents (30.5 ± 6.0 Hz in CT, 11.0 ± 2.2 Hz in FRDA for 30 pA of injected current; *P*-value < 0.0001; two-way ANOVA with Tukey’s test for multiple comparisons) (Fig. 10Bv). Two different patterns were observed also in ISO CT culture replicates, defined again as Type 1 (nine neurons in five replicates) and Type 2 (seven neurons in three replicates). As for Type 1 FRDA neurons, Type 1 ISO CT neurons showed no differences compared to CTs (Fig. 8Civ), whereas Type 2 ISO CT neurons showed delayed accommodation, though maintaining a similar AP frequency profile than CT neurons in response to increasing injected currents up to 50 pA (30.5 ± 6.0 Hz in CT, 27.3 ± 3.4 Hz in ISO CT for 30 pA of injected current, *P*-value = 0.96; two-way ANOVA with Tukey’s test for multiple comparisons) (Fig. 10Cv).

The different behaviours observed for FRDA and ISO CT neurons in their comparison to CTs suggested an alteration in the expression or function (kinetics or sensitivity) of ion channels involved in firing frequency regulation and firing pattern determination, with particular regard to voltage-gated ion channels. However, the complexity of the proprioceptive system, which involves different classes of ion channels with diverse sensitivities to mechanical, chemical and electrical stimuli, does not allow any clear conclusions at this stage and calls for a deeper investigation of the observed differences.^25,54–56^

## Discussion

We herein present an in-depth characterization of hiPSC-derived sensory neuronal cultures highly enriched for PPNs, whose abnormal development and degeneration are hallmarks of FRDA.

We attempted to address the question of the high sensitivity of PPNs in FRDA by *in vitro* characterization of FRDA, CT and ISO CT hiPSC-derived sensory neurons at the genetic, transcriptomic, proteomic, morphological and electrophysiological level. The inclusion of FRDA sibling ISO CT lines also helped us to address the question if the removal of the expanded GAA repeats would be sufficient to fully correct the phenotype of FRDA cells.

As for primary fibroblasts, lymphoblasts, tissues from FRDA patients and animal models of the disease,^9,20,57^ we confirmed the epigenetic repression of the *FXN* locus in mature sensory neurons with the contribution of different effectors depending on the distance from the GAA repeat expansion. While reduced acetylation of H3K9 and H3K27 was prominent in the coding region and proximal intronic site, increased H3K9 and H3K27 trimethylation prevailed in the regions flanking the GAA expansion mutation, implying the action of both SUV39H1/HP1 and PRC2. Our observations are in line with the DNA methylation profile at the *FXN* locus.^22,23^ We also suggest the existence of a physiological silencing of the intronic region flanking the GAA repeats in CT neurons, as indicated by the combined reduced acetylation and increased trimethylation of H3K9 and H3K27 in that site. The removal of the GAA expansion mutation in ISO CT neurons was only able to induce a partial reversal of the repressive marks, which was, however, sufficient to restore frataxin expression.

The transcriptomic and proteomic analyses of our cultures indicated a dysregulation of pathways involved in the organization of axonal cytoskeleton at the growth cone, in neurite extension and axon guidance, and, mostly at later stages of maturation, in synaptic plasticity and chemical transmission. Numerous altered markers were already present in immature neurons, supporting the existence of a developmental component in FRDA. These findings were strengthened by the defects observed at the morphological levels in FRDA cells. Differentiating neurons showed the ability to extend very long processes in cultures, but FRDA neurites exhibited a more complex and tortuous course.

Our study also revealed signs of oxidative and mitochondrial stress, although the involved factors were not the same as reported in other FRDA models^10–16,58–60^ such as SOD2, NRF2 and iron regulatory proteins, which were not differentially expressed in our cultures. However, our study did not assess post-translational modifications affecting the functional properties of these proteins, including the presence of Fe-S clusters in some of them. This aspect can be assessed in future studies.

Finally, the electrophysiological analysis of our cultures detected irregular firing properties in tonic FRDA and ISO CT neurons, although only FRDA neurons also exhibited a reduction of their firing frequencies in comparison to CTs, with a partial recovery observed in ISO CT cells. A common feature was, instead, the delayed accommodation in response to sustained depolarizing stimuli: this could be the consequence of alterations in the expression or in the kinetics of inactivation of sodium channels or in the potassium conductance through the membrane, both of which are involved in the regulation of adaptation to continuous stimulations in those neurons. However, the electrical response of proprioceptive neurons to stimulation is complex and involves different types of mechanosensitive and voltage-gated ion channels.^25,54–56^ The complexity of these events makes the interpretation of our results difficult at this stage. A deeper investigation of the different channels and interactions involved in proprioceptive signalling is needed.

Taken together, our results suggest that in FRDA, PPNs might not be able to properly reach and innervate their targets in muscles or in the spinal cord. Since proper targeting is critical for neuronal survival, this could consequently lead to proprioceptive degeneration. Our findings are in line with other recent studies involving the usage of hiPSC-derived mixed sensory neuronal cultures or DRG organoids:^15,16^ affected sensory neurons showed a dysregulation of pathways related to axonogenesis and chemical synaptic transmission, while DRG organoids derived from FRDA patients displayed a severe impairment in axonal spreading *in vitro*, along with the inability to form proper contacts with intrafusal fibres in DRG-muscle cell co-cultures.

Interestingly, a recovery of the pathological features in DRG organoids was observed only after the excision of the entire *FXN* intron 1 and not only the region flanking the GAA expansion mutation. However, in that case the limited excision of the GAA repeat did not lead to full recovery of frataxin levels, while in our ISO CT lines frataxin levels were comparable to CT lines. We suspect that the persisting differences may represent non-completely erased homeostatic responses to frataxin deficiency in the original FRDA lines, as supported by similar changes in non-coding RNAs in FRDA and ISO CT lines. Many of these transcripts were either massively upregulated or downregulated both in FRDA and ISO CT lines at all stages of differentiation. A deeper investigation of these regulatory factors may help in elucidating pathogenic processes and homeostatic responses in FRDA.

Our study also confirmed changes in RUNX3 and TRKC-NT3 signalling, the main intrinsic and extrinsic factors in PPN survival and specification. RUNX3, which we found downregulated in FRDA PPNs, plays a critical role in proprioceptive pathfinding,^38,39^ while the TRKC-NT3 signalling regulates many pathways that promote neurite outgrowth and synaptic plasticity,^60^ which we found altered in our study. This is supported by the observation that animal models of RUNX3 and TRKC deficiency resemble many pathological features observed in FRDA.^39,61,62^

In conclusion, our analysis led to the identification of a significant number of differentially expressed genes and proteins that could play a critical role in the determination of the pathological features of FRDA. However, further and more detailed investigations are needed to highlight the contribution and the specific role of these elements. At this stage, in fact, it is difficult to address which of these markers or biological processes are the result of the disease and which could be the cause. These studies may also help addressing some unsolved questions in FRDA^63^ from the relatively high levels of frataxin in FRDA patients compared to other loss-of-function disorders, to the equivocal role of ROS production as main cause of cellular dysfunction and death and to the high sensitivity of a limited number of cell types despite the extensive distribution and expression of frataxin in the body.

## Supplementary Material

fcad007_Supplementary_Data

## Abbreviations

3m =: Trimethylation
aCSF =: artificial CSF
AC =: Acetylation
ChIP =: Chromatin Immunoprecipitation
Cp =: Membrane Capacitance
DDA =: Data-dependent acquisition
DEG =: Differentially expressed gene
DEP =: Differentially expressed protein
DIA =: Data-independent acquisition
DRG =: Dorsal root ganglion
FC =: Fold change
FDR =: False discovery rate
Fe-S =: Iron-Sulfur
FRDA =: Friedreich ataxia
GO =: Gene ontology
H3K27 =: Lysine 27 of histone 3
hiPSCs =: Human induced-pluripotent stem cells
ISC =: Iron-sulfur cluster
ISO CT =: Isogenic control
KO =: Knock out
NcRNA =: Non-coding RNA
PBMCs =: Peripheral blood mononuclear cells
PCA =: Principal component analysis
PRC2 =: Polycomb repressive complex 2
PPN =: Primary proprioceptive neuron
Rm =: Membrane resistance
RMP =: Resting membrane potential
RNA-seq =: RNA-sequencing
ROS =: Reactive oxygen species
τ =: Tau. Membrane time constant
