# Supplementary material for: Proprioceptors-enriched neuronal cultures from induced pluripotent stem cells from Friedreich ataxia patients show altered transcriptomic and proteomic profiles, abnormal neurite extension, and impaired electrophysiological properties
Source: Brain Commun. 2023 Jan 18;5(1):fcad007. doi: 10.1093/braincomms/fcad007 (PMC9972525; doi:10.1093/braincomms/fcad007)
Supplement: fcad007_Supplementary_Data [file fcad007_Supplementary_Data.pdf]

Supplementary Figure 1. Direct comparison of histone post-translational modifications in two FRDA and corresponding ISO CT fully differentiated sensory neuronal cultures

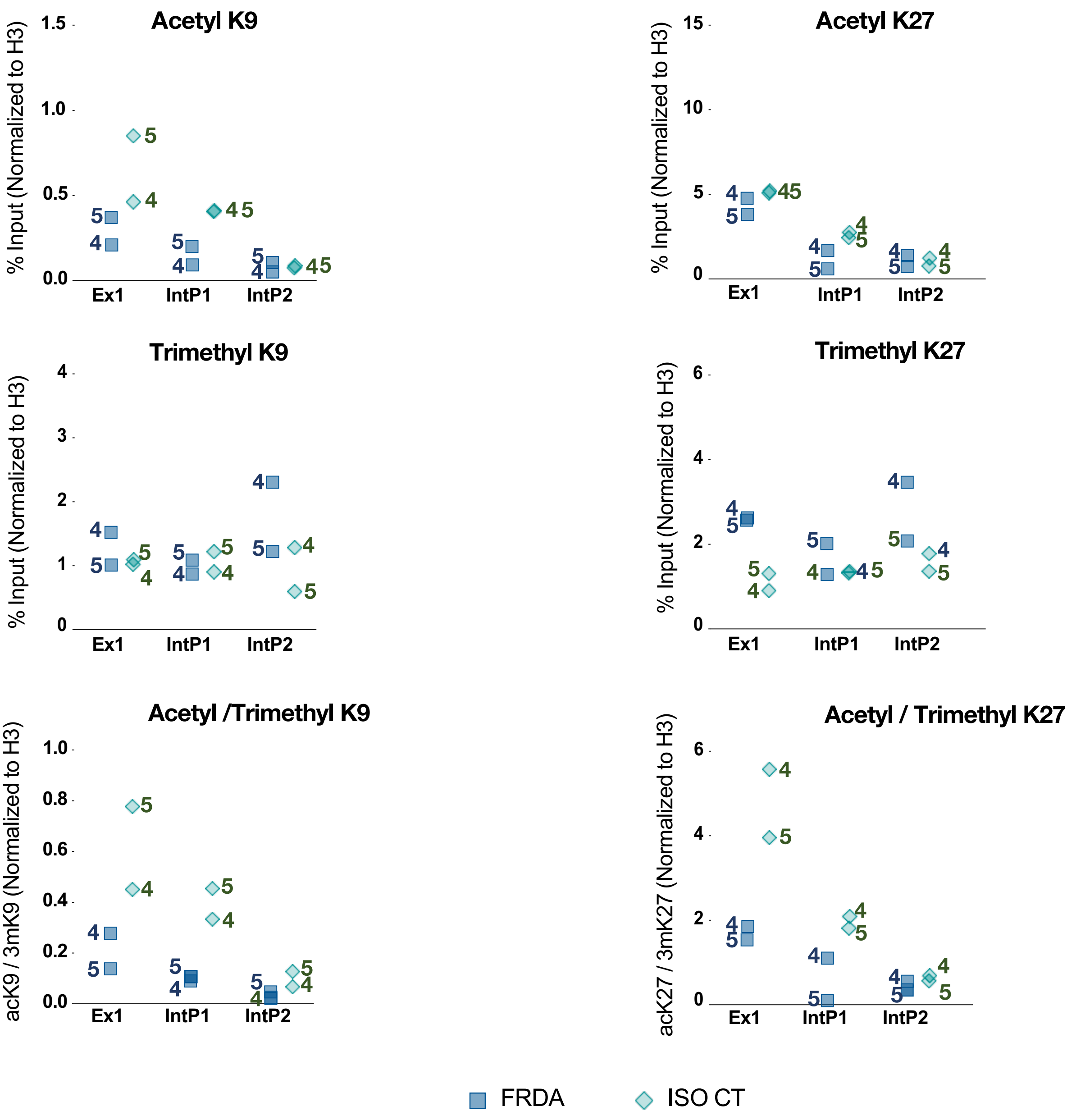

Supplementary Figure 2. Western Blot analysis of frataxin expression

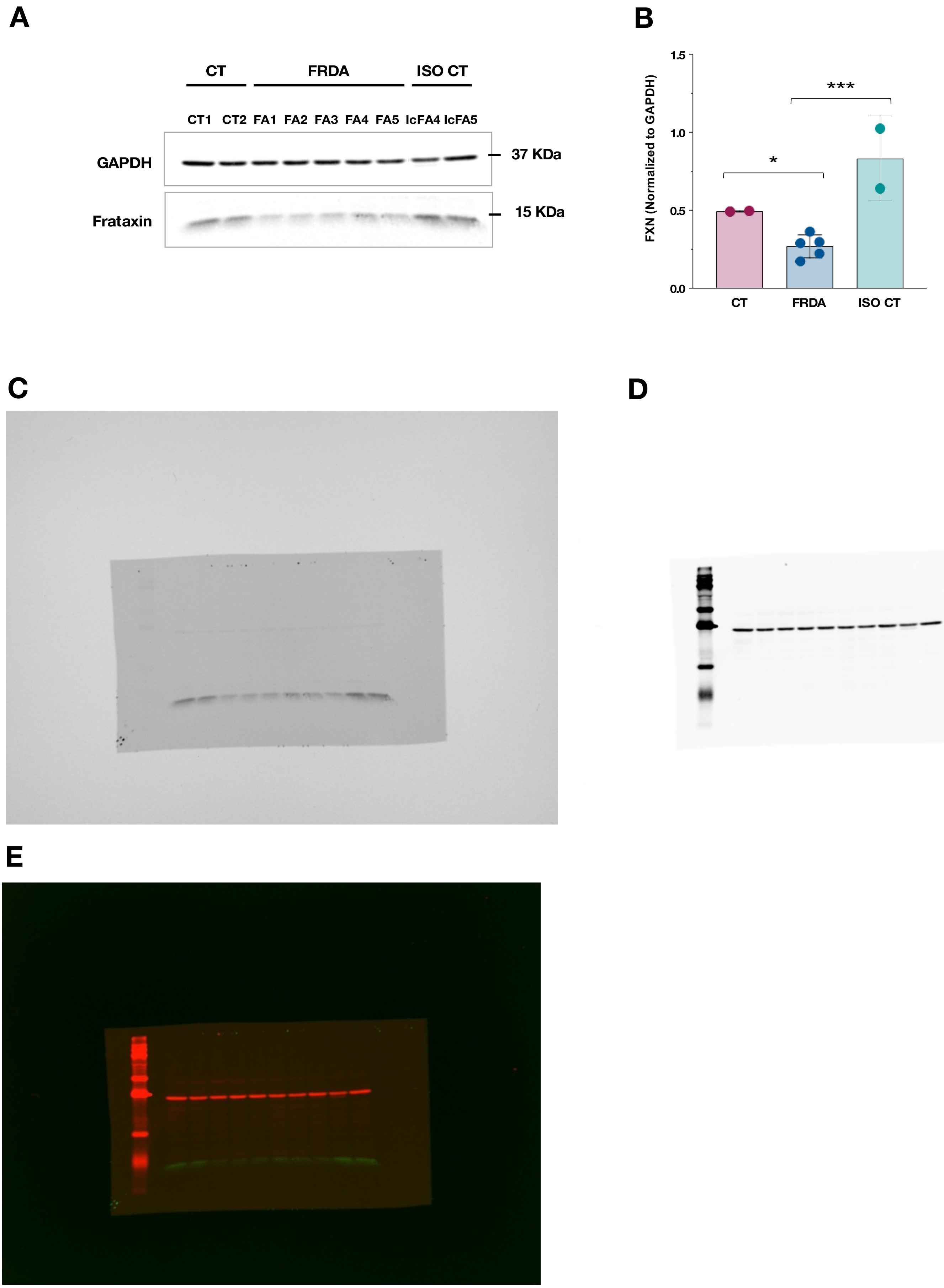

Supplementary Figure 3. Transcriptome profiling of differentiating isogenic control cultures

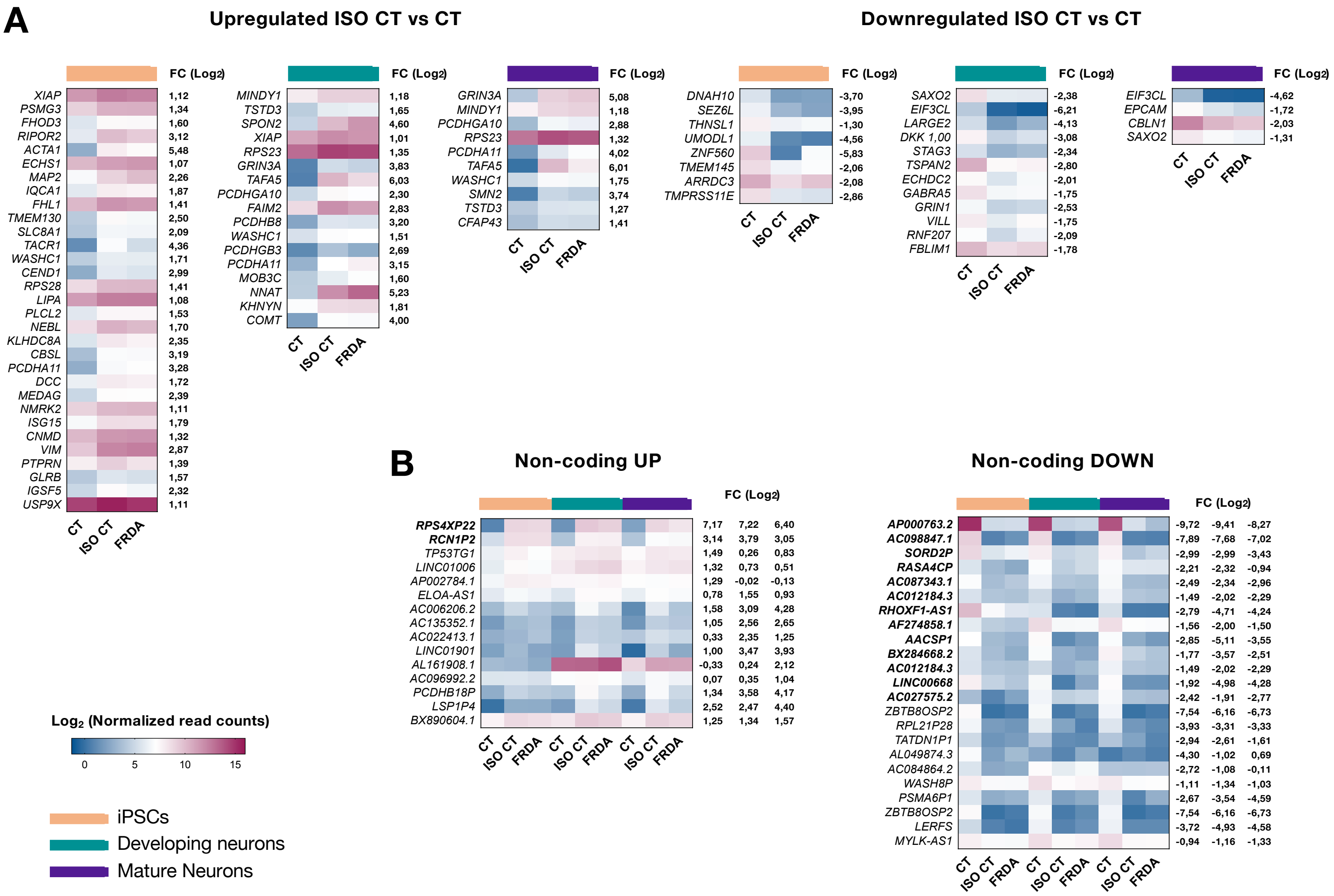

Supplementary Figure 4. Analysis of sensory neuronal markers in differentiated cultures

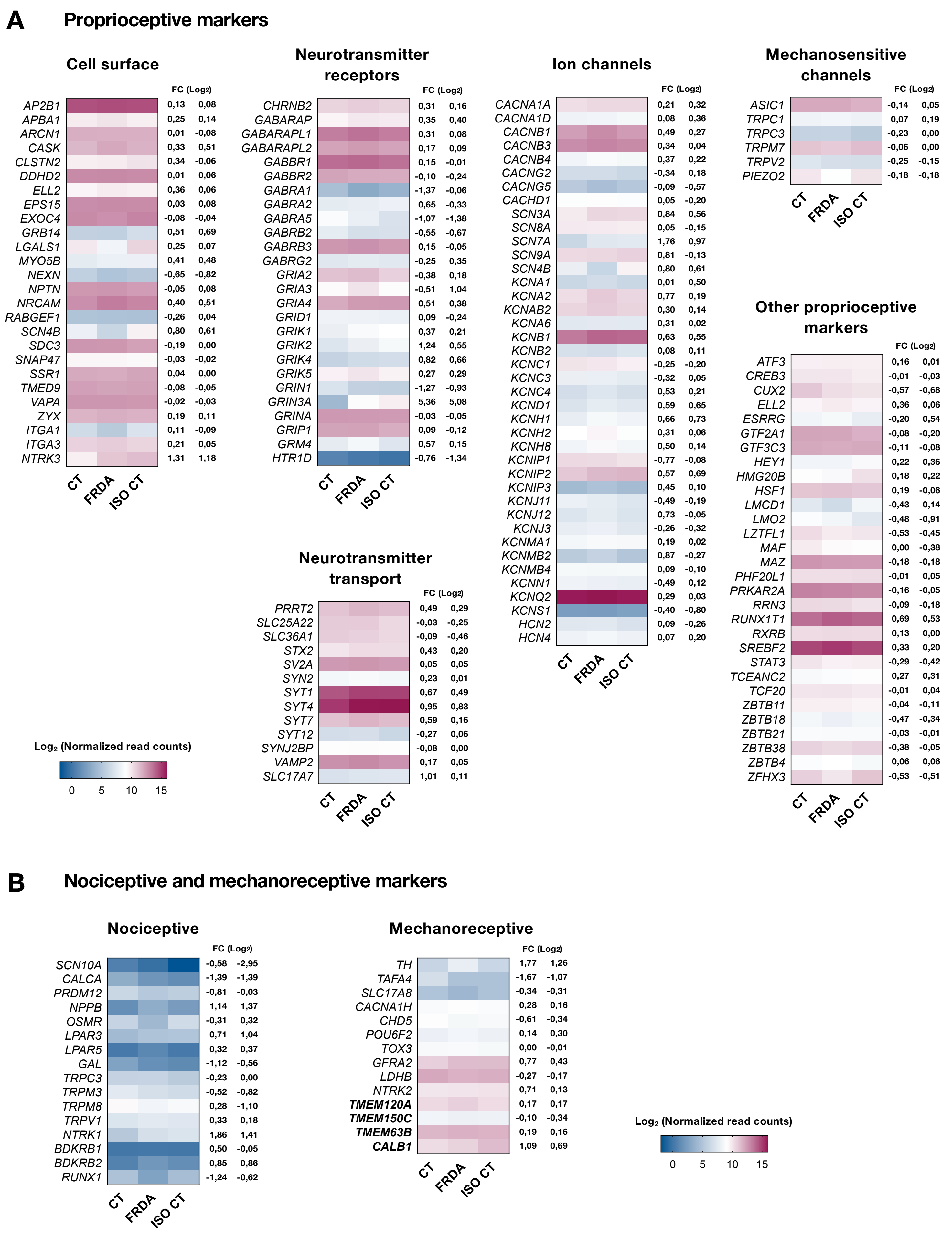

Supplementary Figure 5. Transcriptome profile comparison of FA4 e FA5 with IcFA4 and IcFA5 for differentially expressed genes (DEGs).

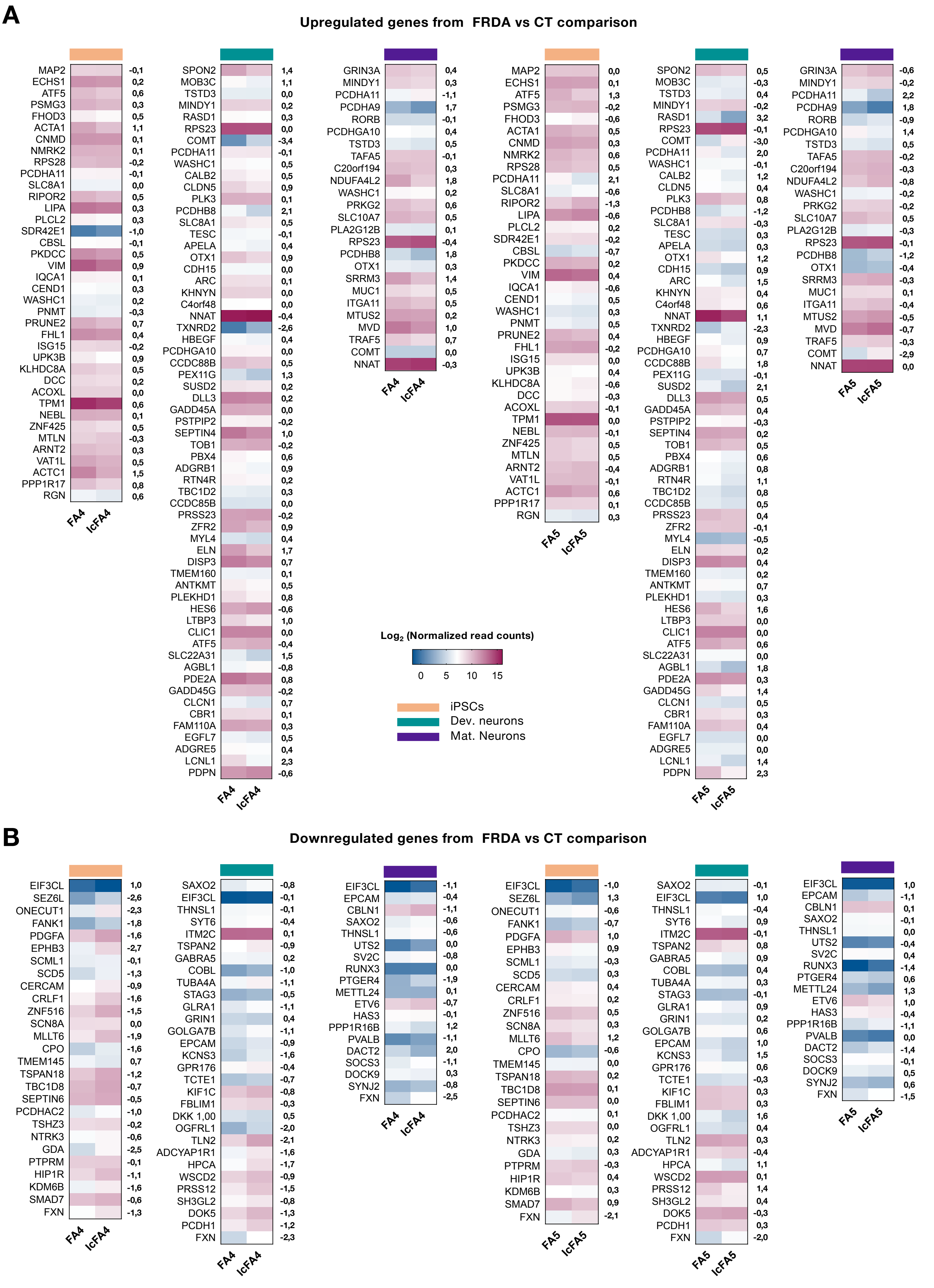

Supplementary Figure 6. Transcriptome profile comparison of FRDA, CT and ISO CT lines for non-coding RNAs

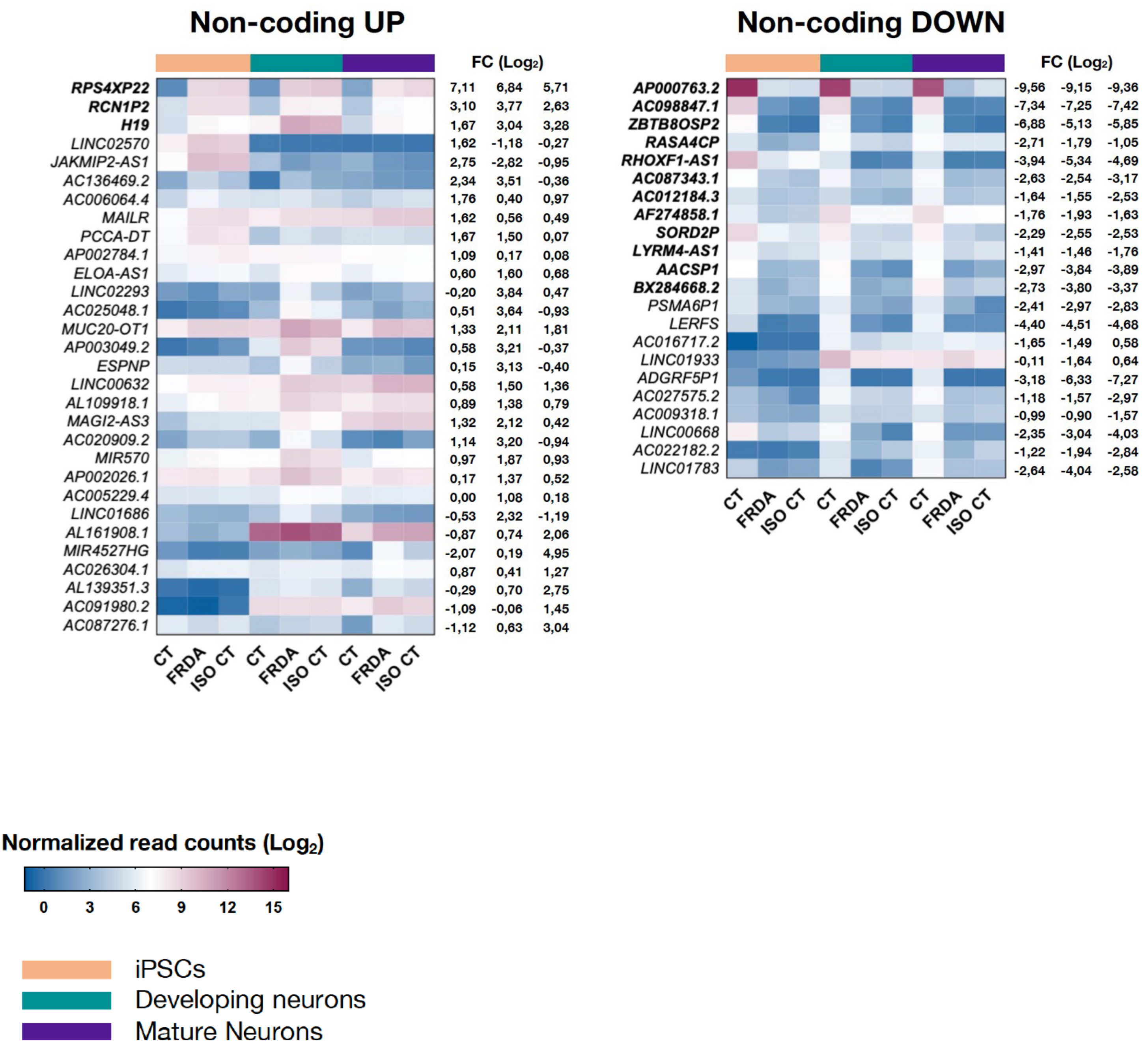

Supplementary Figure 7. Volcano plots and Venn diagrams of DEPs in FRDA, CT and ISO CT neurons.

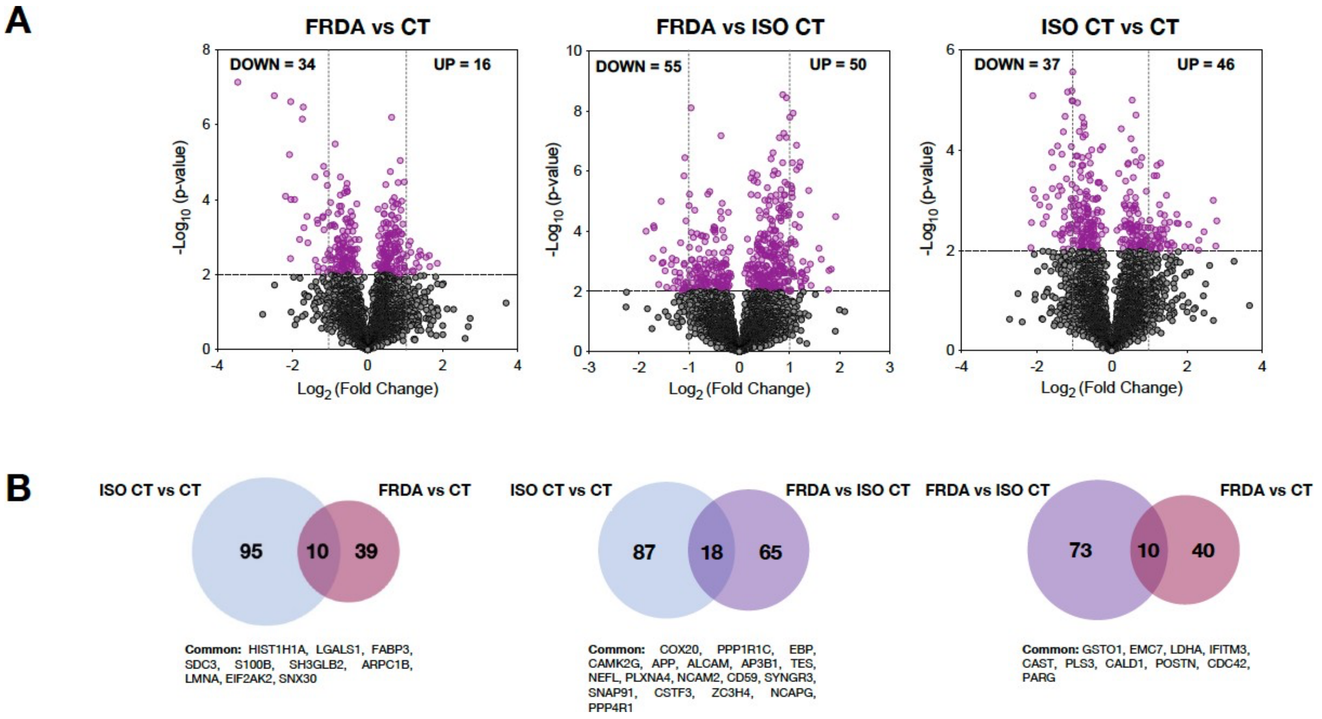

Supplementary Figure 8. Proteome profile comparison of FA4 e FA5 with lcFA4 and lcFA5 for differentially expressed proteins (DEPs).

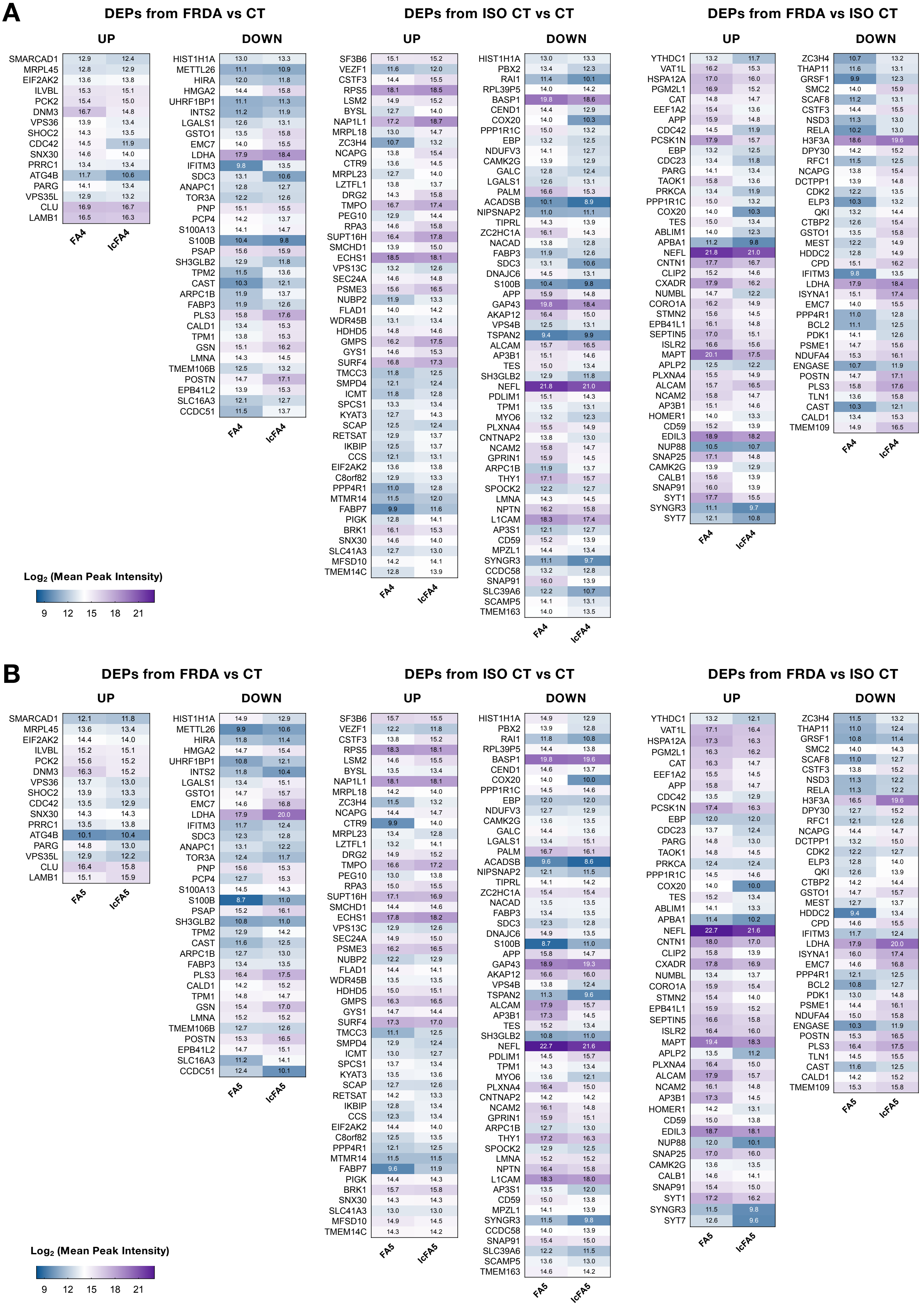

Supplementary Figure 1. Direct comparison of histone post-translational modifications in two FRDA and corresponding IOS CT fully differentiated neuronal cultures. Investigation of Histone H3 post-translational modifications of *FXN* 5'-end at exon 1 (Ex1), intronic region (intron 1) upstream (IntP1, IntP2) of GAA expansion as reported in Figure 1. This is an excerpt from Figure 1 in which for clarity results are only shown for ISO CT IcFA4 and IcFA5 (green diamonds) and corresponding FRDA lines FA 4 and FA5 (blue squares), labeled as 4 and 5.

Supplementary Figure 2. Western Blot analysis of frataxin expression. Frataxin expression was evaluated by Western Blot in differentiated fully mature neurons in two independent biological replicates per line (n = 2; A: representative blot). Protein expression was normalized to GAPDH. Mean values obtained for each group (CT, FRDA and ISO CT) were compared (Mean ± SD). Histograms (B) represent mean values of biological replicates for each group, and error bars represent the standard deviation of mean (SD). Each dot represents the mean of two independent biological replicates per line. A significant decrease in Frataxin expression was observed in FRDA lines compared with CT, along with a recovery of frataxin expression in ISO CT neurons. No significant differences were observed between CT and IC lines (two-way ANOVA with Bonferroni's test for multiple comparisons; * $p < 0.05$; *** $p < 0.001$). CT: Control; FRDA: Friedreich Ataxia; ISO CT: Isogenic Control. Original uncropped gel images are in C (Frataxin), D (GAPDH), E (Frataxin+GAPDH two-color image). Uncropped gels contain one extra sample (third lane from the right) corresponding to an FRDA line that was not further utilized in the analyses because of aberrant growth and altered karyotype.

Supplementary Figure 3. Transcriptome profiling of differentiating ISO CT cultures. **(A)** Heatmaps representing Differentially Expressed Coding Genes between ISO CT and CT (|Log_2(Fold Change)| > 1; adjusted p-value < 0.1), for each stage of differentiation (yellow: iPSCs; green: developing neurons; violet: mature neurons). For each gene, the log_2(Fold Change) between ISO CT and CT lines is shown on the right side of the corresponding heatmap. For each comparison, the transcriptional level of identified DEGs was represented also for the FRDA group. Colour scale represents log_2(Normalized read counts) values. **(B)** Heatmaps representing differentially expressed

were analysed and represented as in (B). For each DEG, the log_2(Fold Change) between ISO CT and CT lines at each stage of differentiation is shown on the right side of the corresponding heatmap (iPSCs: left; developing neurons: middle; mature neurons: right). Non-coding DEGs common to all stages of differentiation are highlighted in bold. (n=2 for CT; n=5 for FRDA; n=3 for ISO CT).

Supplementary Figure 4. Analysis of sensory neuronal markers in differentiated cultures. Heatmaps representing sensory neuron-specific transcripts detected in differentiated cultures. (A) Proprioceptive-specific genes were schematically separated in categories based on their function or cellular localization. (B) Nociceptive and mechanoreceptive-specific genes expressed in differentiated cultures. Genes common to mechanoreceptive and proprioceptive neurons are highlighted in bold. For each gene, the log2(Fold Change) between FRDA and CT neurons (left) or ISO CT and CT neurons (right) is shown on the right side of the corresponding heatmap. Color scale represents log2(normalized read counts) of transcripts.

Supplementary Figure 5. Transcriptome profile comparison of FA4 e FA5 with IcFA4 and IcFA5 for differentially expressed genes (DEGs). A direct comparison between FRDA and their sibling ISO CT lines was performed for all identified DEGs at the transcriptomic analysis. DEG expression was specifically investigated for upregulated (A) and downregulated (B) genes for lines FA4 and IcFA4 and FA5 and IcFA5, to evaluate the effects induced on gene expression by the removal of the GAA expansion mutation in genetically matched cell lines. For each gene, the ratio between FA4 and IcFA4 or FA5 and IcFA5 is represented on the ride side of the corresponding heatmap, in log2 scale. Color scale represents log2(normalized read counts) of transcripts. (n=1 for all lines).

Supplementary Figure 6. Transcriptome profile comparison of FRDA, CT and ISO CT lines for non-coding RNAs. Heatmaps representing differentially expressed non-coding genes or pseudogenes between FRDA and CT lines, at all stages of differentiation. For each DEG, the log_2(Fold Change) between FRDA and CT lines at each stage of differentiation is shown on the right side of the corresponding heatmap (iPSCs: left; developing neurons: middle; mature neurons:

Supplementary Figure 7. Volcano plots and Venn diagrams of DEPs in FRDA, CT and ISO CT neurons. **(A)** Volcano plots of significantly differentially expressed proteins in the comparison between FRDA and CT neurons (left), FRDA and ISO CT neurons (middle) and ISO CT and CT neurons (right). Red dots represent proteins exhibiting significant fold changes (p-value < 0.01). Of those, only proteins with absolute Log2(Fold Change) > 1 were selected for analysis (left and right sides of vertical broken lines). **(B)** Venn Diagrams of identified differentially expressed proteins in the comparison of FRDA and ISO CT neurons versus CT (left), FRDA and CT neurons versus ISO CT (middle) or CT and ISO CT neurons versus FRDA (right). Common identified proteins are indicated under the corresponding diagram.

Supplementary Figure 8. Proteome profile comparison of FA4 e FA5 with IcFA4 and IcFA5 for differentially expressed proteins (DEPs). A direct comparison between FRDA and their sibling ISO CT lines was performed for all identified DEPs at the proteomic analysis. DEP expression was specifically investigated for lines FA4 and IcFA4 (A) and FA5 and IcFA5 (B), to evaluate the effects induced on protein expression by the removal of the GAA expansion mutation in genetically matched cell lines. Color scale represents log2(Mean Peak Intensity) values of identified protein spectra. Exact values of Mean Peak Intensities for each protein are indicated in the heatmaps. (n=1 for all lines).

Supplementary Table 1. List of primers used for Chromatin Immunoprecipitation

RT-qPCR Primers	Sequence (5' – 3')
Ex1 Forward	GGAGCAGCATGTGGACTCTC
Ex1 Reverse	CGGCGCGGATACTTACTG
IntP1 Forward	CTCCCGGTTGCATTTACACT
IntP1 Reverse	GTGACAAGCATGGAGACAGC
IntP2 Forward	CTGACCCGACCTTTATTCCA
IntP2 Reverse	TGGGCGTCACCTTTATCTTC
IntP3 Forward	GAAACCCAAAGAATGGCTGTG
IntP3 Reverse	TTCCCTCCTCGTGAAACACC
IntP4 Forward	CTGGAAAAATAGGCAAGTGTGG
IntP4 Reverse	CAGGGGTGGAAGCCCAATAC
IntP5 Forward	CCCTTGCACATCTTGGGTAT
IntP5 Reverse	GAGAAAAGGGTGGGGAAGAG

Supplementary Table 2. List of identified differentially expressed coding and non-coding genes for iPSCs, developing neurons and mature neurons.

DIFFERENTIALLY EXPRESSED GENES – iPSCs																
UPREGULATED GENES																
Normalized read counts									GENE	Description	Chr	GO: Biological process	Log_2FC FRDA/CT	FDR	Log_2FC ISO CT/CT	FDR
iPSCs			Developing Neurons			Mature Neurons										
CT	FRDA	ISO CT	CT	FRDA	ISO CT	CT	FRDA	ISO CT								
**104**	**1068**	**554**	48000	56547	77763	43223	52417	54091	**MAP2**	microtubule associated protein 2	2	microtubule bundle formation; central nervous system neuron development; microtubule cytoskeleton organization; neuron projection development; regulation of cellular protein localization; regulation of microtubule polymerization; dendrite development; negative regulation of axon extension; negative regulation of microtubule polymerization; dendrite morphogenesis; positive regulation of anterograde dense core granule transport; regulation of organelle transport along microtubule; positive regulation of anterograde synaptic vesicle transport; negative regulation of microtubule binding; negative regulation of microtubule motor activity; cellular response to organic substance; axonogenesis; establishment of cell polarity	3,16	1,8E-08	2,26	4,8E-03
**1077**	**2726**	**2265**	1583	2731	2455	1204	1847	1961	**ECHS1**	enoyl-CoA hydratase, short chain 1	10	lipid metabolic process; fatty acid metabolic process; fatty acid beta-oxidation	1,33	8,0E-07	1,07	3,9E-03
**328**	**1210**	**574**	646	1364	1120	568	741	640	**ATF5**	activating transcription factor 5	19	regulation of transcription, DNA-templated; regulation of transcription by RNA polymerase II; negative regulation of apoptotic process; positive regulation of transcription, DNA-templated; fat cell differentiation; negative regulation of cell population proliferation; positive regulation of transcription by RNA polymerase II; negative regulation of transcription, DNA-templated; regulation of centrosome cycle; negative regulation of cell cycle G2/M phase transition; cerebellar granule cell precursor proliferation; circadian rhythm; post-embryonic development; regulation of gene expression; olfactory bulb interneuron differentiation; olfactory bulb interneuron development; olfactory lobe development; multicellular organism growth	1,86	3,1E-06	0,79	6,6E-01
**521**	**1375**	**1331**	397	795	721	394	754	690	**PSMG3**	proteasome assembly chaperone 3	7	chaperone-mediated protein complex assembly	1,39	8,8E-06	1,34	4,3E-04
**51**	**163**	**157**	821	743	782	777	765	756	**FHOD3**	formin homology 2 domain containing 3	18	actin filament organization; cortical actin cytoskeleton organization; cardiac myofibril assembly; sarcomere organization; actin filament network formation; negative regulation of actin filament polymerization	1,64	1,1E-05	1,60	4,3E-04
**7**	**746**	**721**	48	468	76	6	6	5	**ACTA1**	actin alpha 1, skeletal muscle	1	positive regulation of gene expression; mesenchyme migration; muscle filament sliding; skeletal muscle thin filament assembly; muscle contraction; skeletal muscle fiber development; response to mechanical stimulus; response to extracellular stimulus; response to lithium ion; skeletal muscle fiber adaptation; response to steroid hormone; cellular response to organonitrogen compound	5,68	7,1E-05	5,48	1,6E-03
**1063**	**2973**	**2627**	9	2	7	5	3	4	**CNMD**	chondromodulin	13	multicellular organism development; cell differentiation; cartilage development; negative regulation of angiogenesis; skeletal system development; endothelial cell morphogenesis; negative regulation of endothelial cell proliferation; proteoglycan metabolic process; negative regulation of vascular endothelial growth factor receptor signaling pathway	1,52	9,3E-04	1,32	4,5E-02
**496**	**1160**	**1062**	5	4	3	0	3	3	**NMRK2**	nicotinamide riboside kinase 2	19	phosphorylation; NAD biosynthetic process; pyridine nucleotide biosynthetic process; NAD metabolic process; negative regulation of myoblast differentiation	1,25	1,2E-03	1,11	4,1E-02
**397**	**1117**	**1068**	1016	1458	1513	683	1189	1199	**RPS28**	ribosomal protein S28	19	translation; cytoplasmic translation; rRNA processing; ribosome biogenesis; ribosomal small subunit biogenesis; ribosomal small subunit assembly; viral transcription; translational initiation; maturation of SSU-rRNA; nuclear-transcribed mRNA catabolic process, nonsense-mediated decay; SRP-dependent cotranslational protein targeting to membrane	1,47	1,4E-03	1,41	2,0E-02
**10**	**144**	**122**	15	213	161	4	124	88	**PCDHA11**	protocadherin alpha 11	5	homophilic cell adhesion via plasma membrane adhesion molecules; cell adhesion; nervous system development	3,53	1,7E-03	3,28	3,1E-02
**20**	**90**	**92**	105	408	253	515	1063	892	**SLC8A1**	solute carrier family 8 member A1	2	transmembrane transport; calcium ion transport; cell communication; regulation of cardiac muscle contraction by regulation of the release of sequestered calcium ion; negative regulation of protein serine/threonine kinase activity; cellular response to reactive oxygen species; regulation of heart rate; ion transport; sodium ion transport; calcium ion transmembrane transport; sodium ion transmembrane transport; calcium ion import across plasma membrane; positive regulation of bone mineralization; muscle contraction; regulation of cardiac conduction; regulation of cell communication by electrical coupling; cardiac muscle contraction; cellular response to caffeine; calcium ion import; response to muscle stretch; calcium ion homeostasis; cellular calcium ion homeostasis; membrane depolarization during cardiac muscle cell action potential; cellular sodium ion homeostasis; sodium ion export across plasma membrane; relaxation of cardiac muscle; cell communication by electrical coupling involved in cardiac conduction; regulation of cardiac muscle contraction by calcium ion signaling; regulation of gene expression; regulation of the force of heart contraction; sodium ion import across plasma membrane; positive regulation of the force of heart contraction; vascular associated smooth muscle contraction; metal ion transport; relaxation of smooth muscle; negative regulation of cytosolic calcium ion concentration; cardiac muscle cell development; cytosolic calcium ion transport; calcium ion transport into cytosol; regulation of postsynaptic cytosolic calcium ion concentration; response to hypoxia; regulation of sodium ion transport; positive regulation of cytosolic calcium ion concentration; response to nutrient; response to glucose; positive regulation of fibroblast migration; telencephalon development; response to ATP; response to immobilization stress; response to drug; response to hydrogen peroxide; regulation of calcium ion transport; cellular response to cAMP; cellular response to hypoxia; calcium ion export	2,04	2,0E-03	2,09	9,8E-03
**107**	**696**	**1051**	1689	1424	1415	600	1188	1057	**RIPOR2**	RHO family interacting cell polarization regulator 2	6	sensory perception of sound; skeletal muscle fiber development; cell adhesion; multicellular organism development; cell differentiation; chemotaxis; negative regulation of cell adhesion; muscle organ development; negative regulation of signal transduction; negative regulation of Rho protein signal transduction; establishment of protein localization; protein homooligomerization; positive regulation of filopodium assembly; auditory receptor cell stereocilium organization; cellular response to	2,54	2,0E-03	3,12	4,3E-04

												mechanical stimulus; positive regulation of neutrophil chemotaxis; negative regulation of protein localization to cell leading edge; cellular response to chemokine; regulation of establishment of cell polarity; positive regulation of neutrophil extravasation; negative regulation of Rho guanyl-nucleotide exchange factor activity; positive regulation of myoblast fusion; negative regulation of T cell proliferation; negative regulation of T cell migration; positive regulation of myoblast differentiation; negative regulation of establishment of T cell polarity; positive regulation of cell cycle arrest; regulation of mitotic spindle assembly				
**2440**	**5292**	**5172**	3087	3331	3054	1872	2342	2215	**LIPA**	lipase A, lysosomal acid type	10	lipid metabolic process; lipid catabolic process; low-density lipoprotein particle clearance; sterol metabolic process; cell morphogenesis; cytokine production; inflammatory response; cell population proliferation; lung development; tissue remodeling; homeostasis of number of cells within a tissue	1,11	2,2E-03	1,08	2,8E-02
**57**	**174**	**168**	402	528	540	391	507	485	**PLCL2**	phospholipase C like 2	3	signal transduction; intracellular signal transduction; lipid metabolic process; regulation of synaptic transmission, GABAergic; negative regulation of cold-induced thermogenesis; phosphatidylinositol-mediated signaling; B cell proliferation involved in immune response; B-1a B cell differentiation; gamma-aminobutyric acid signaling pathway; regulation of peptidyl-serine phosphorylation; negative regulation of B cell receptor signaling pathway; positive regulation of receptor binding; lipid catabolic process	1,58	2,5E-03	1,53	2,9E-02
**4**	**447**	**153**	10	135	80	6	71	42	**SDR42E1**	short chain dehydrogenase/reductase family 42E, member 1	16	oxidation-reduction process; steroid biosynthetic process	5,26	2,7E-03	3,78	2,9E-01
**13**	**132**	**136**	2	13	13	2	12	10	**CBSL**	cystathionine beta-synthase like	21	cysteine biosynthetic process from serine; cysteine biosynthetic process via cystathionine; cellular amino acid biosynthetic process; cysteine biosynthetic process; transsulfuration; homocysteine metabolic process; hydrogen sulfide biosynthetic process	3,21	4,5E-03	3,19	3,1E-02
**1044**	**3873**	**2038**	3940	3960	4163	1906	1970	2492	**PKDCC**	protein kinase domain containing, cytoplasmic	2	protein phosphorylation; protein transport; phosphorylation; multicellular organism development; cell differentiation; ossification; skeletal system development; positive regulation of bone mineralization; positive regulation of chondrocyte differentiation; roof of mouth development; lung alveolus development; peptidyl-tyrosine phosphorylation; embryonic digestive tract development; negative regulation of Golgi to plasma membrane protein transport; bone mineralization; limb morphogenesis; multicellular organism growth	1,85	4,7E-03	0,93	8,4E-01
**685**	**7241**	**6236**	9283	6532	8626	29875	34820	26030	**VIM**	vimentin	10	viral process; cellular response to lipopolysaccharide; cytokine-mediated signaling pathway; positive regulation of translation; muscle filament sliding; cellular response to muramyl dipeptide; intermediate filament organization; positive regulation of gene expression; negative regulation of neuron projection development; astrocyte development; positive regulation of collagen biosynthetic process; regulation of mRNA stability; intermediate filament-based process; Bergmann glial cell differentiation; SMAD protein signal transduction; lens fiber cell development; cellular response to interferon-gamma	3,07	4,8E-03	2,87	5,6E-02
**61**	**189**	**226**	9	17	11	8	17	27	**IQCA1**	IQ motif containing with AAA domain 1	2		1,63	6,1E-03	1,87	5,5E-03
**6**	**61**	**66**	424	397	277	596	708	678	**CEND1**	cell cycle exit and neuronal differentiation 1	11	cerebellum development; neuron differentiation; cell differentiation; adult walking behavior; cerebellar granular layer maturation; cerebellar Purkinje cell differentiation; radial glia guided migration of cerebellar granule cell; negative regulation of cerebellar granule cell precursor proliferation	2,86	6,1E-03	2,99	1,8E-02
**20**	**62**	**68**	41	130	118	32	99	109	**WASHC1**	WASH complex subunit 1	9	Arp2/3 complex-mediated actin nucleation; protein transport; exocytosis; extracellular matrix disassembly; positive regulation of cell migration; negative regulation of autophagy; endosomal transport; positive regulation of pseudopodium assembly; regulation of protein ubiquitination; retrograde transport, endosome to Golgi; negative regulation of phosphatidylinositol 3-kinase activity; retrograde transport, endosome to plasma membrane	1,57	9,9E-03	1,71	1,8E-02
**24**	**82**	**53**	1	1	0	3	0	0	**PNMT**	phenylethanolamine N-methyltransferase	17	methylation; catecholamine biosynthetic process; epinephrine biosynthetic process	1,78	1,2E-02	1,15	6,3E-01
**319**	**1030**	**878**	1431	1780	1729	1058	1561	1425	**PRUNE2**	prune homolog 2 with BCH domain	9	apoptotic process; polyphosphate catabolic process	1,64	1,2E-02	1,42	2,0E-01
**1085**	**2470**	**2928**	9461	10423	11726	7610	9835	11392	**FHL1**	four and a half LIM domains 1	X	muscle organ development; multicellular organism development; cell differentiation; negative regulation of cell growth; negative regulation of G1/S transition of mitotic cell cycle; regulation of potassium ion transmembrane transporter activity; animal organ morphogenesis; negative regulation of G2/M transition of mitotic cell cycle; positive regulation of potassium ion transport; regulation of membrane depolarization	1,16	1,5E-02	1,41	7,4E-03
**104**	**341**	**368**	126	99	74	64	71	53	**ISG15**	ISG15 ubiquitin like modifier	1	viral process; defense response to virus; immune system process; innate immune response; integrin-mediated signaling pathway; defense response to bacterium; modification-dependent protein catabolic process; negative regulation of viral genome replication; positive regulation of bone mineralization; interferon-gamma production; negative regulation of protein ubiquitination; type I interferon signaling pathway; translesion synthesis; ISG15-protein conjugation; negative regulation of type I interferon production; response to bacterium; interleukin-10 production; regulation of interferon-gamma production; response to type I interferon; positive regulation of erythrocyte differentiation	1,69	1,5E-02	1,79	4,3E-02
**25**	**151**	**106**	1	4	3	0	3	4	**UPK3B**	uroplakin 3B	7	negative regulation of gene expression	2,57	1,7E-02	2,01	3,8E-01
**49**	**243**	**283**	6109	7458	7204	2705	2394	2175	**KLHDC8A**	kelch domain containing 8A	1		2,11	2,0E-02	2,35	3,1E-02
**71**	**222**	**251**	24497	40133	37580	13563	15513	17966	**DCC**	DCC netrin 1 receptor	18	apoptotic process; axon guidance; nervous system development; multicellular organism development; netrin-activated signaling pathway; axonogenesis; negative regulation of netrin-activated signaling pathway; regulation of neuron death; extrinsic apoptotic signaling pathway in absence of ligand; neuron migration; negative regulation of neuron projection development; spinal cord ventral commissure morphogenesis; dorsal/ventral axon guidance; anterior/posterior axon guidance; negative regulation of collateral sprouting; postsynaptic modulation of chemical synaptic transmission; negative regulation of dendrite development	1,52	2,7E-02	1,72	3,3E-02
**267**	**688**	**583**	2	2	2	1	5	2	**ACOXL**	acyl-CoA oxidase like	2	oxidation-reduction process; fatty acid beta-oxidation; fatty acid metabolic process; fatty acid beta-oxidation using acyl-CoA oxidase; lipid homeostasis	1,41	3,7E-02	1,14	4,4E-01
**7408**	**20510**	**21351**	3389	2863	2769	3832	3425	3253	**TPM1**	tropomyosin 1	15	negative regulation of vascular associated smooth muscle cell proliferation; negative regulation of vascular associated smooth muscle cell migration; negative regulation of cell migration; cellular response to reactive oxygen species; regulation of cell shape; actin filament organization; ruffle organization; positive regulation of stress fiber assembly; muscle contraction; cardiac muscle contraction; ventricular cardiac muscle tissue morphogenesis; muscle filament sliding; sarcomere organization; wound healing; regulation of muscle contraction; cytoskeleton organization; regulation of heart contraction; positive regulation of cell adhesion; positive regulation of ATPase activity; positive regulation of heart rate by epinephrine; in utero embryonic development	1,46	3,8E-02	1,51	1,2E-01

389	1083	1313	7873	8484	8831	6143	5941	6777	NEBL	nebulette	10	muscle fiber development; cardiac muscle thin filament assembly	1,41	3,9E-02	1,70	2,9E-02
228	466	381	617	719	887	552	674	699	ZNF425	zinc finger protein 425	7	regulation of transcription, DNA-templated; regulation of transcription by RNA polymerase II; negative regulation of transcription by RNA polymerase II; negative regulation of transcription, DNA-templated	1,02	4,6E-02	0,73	6,6E-01
338	726	582	182	233	202	222	228	203	MTLN	mitoregulin	2	positive regulation of protein-containing complex assembly; positive regulation of mitochondrial membrane potential; positive regulation of sequestering of calcium ion	1,10	4,7E-02	0,78	6,8E-01
337	789	831	3001	3313	3472	5298	5382	5450	ARNT2	aryl hydrocarbon receptor nuclear translocator 2	15	regulation of transcription, DNA-templated; positive regulation of transcription, DNA-templated; positive regulation of transcription by RNA polymerase II; response to hypoxia; xenobiotic metabolic process; brain development; in utero embryonic development; central nervous system development; positive regulation of cell population proliferation; response to estradiol; negative regulation of apoptotic process	1,19	6,2E-02	1,27	1,3E-01
246	1400	1158	336	268	332	4458	5744	3670	VAT1L	vesicle amine transport 1 like	16	oxidation-reduction process	2,33	6,2E-02	2,06	3,9E-01
206	1969	1292	66	500	88	15	13	12	ACTC1	actin alpha cardiac muscle 1	15	actin filament organization; positive regulation of gene expression; mesenchyme migration; muscle filament sliding; actin filament-based movement; heart contraction; cardiac myofibril assembly; cardiac muscle tissue morphogenesis; actomyosin structure organization; skeletal muscle thin filament assembly; actin-myosin filament sliding; response to drug; negative regulation of apoptotic process; response to ethanol; cardiac muscle contraction; actin-mediated cell contraction	3,13	6,2E-02	2,45	5,7E-01
72	523	462	937	1930	1552	2181	2080	2477	PPP1R17	protein phosphatase 1 regulatory subunit 17	7	regulation of phosphatase activity; negative regulation of phosphoprotein phosphatase activity; negative regulation of catalytic activity; intracellular signal transduction; central nervous system development	2,50	7,4E-02	2,35	3,2E-01
9	45	47	14	27	26	8	10	15	RGN	regucalcin	X	positive regulation of ATPase activity; cellular calcium ion homeostasis; regulation of calcium-mediated signaling; L-ascorbic acid biosynthetic process; kidney development; liver development; negative regulation of protein phosphorylation; negative regulation of protein kinase activity; spermatogenesis; aging; positive regulation of triglyceride biosynthetic process; positive regulation of glucose metabolic process; positive regulation of phosphatase activity; negative regulation of phosphoprotein phosphatase activity; negative regulation of GTPase activity; negative regulation of apoptotic process; positive regulation of GTPase activity; negative regulation of nitric oxide biosynthetic process; positive regulation of fatty acid biosynthetic process; negative regulation of epithelial cell proliferation; negative regulation of cyclic-nucleotide phosphodiesterase activity; liver regeneration; negative regulation of flagellated sperm motility; positive regulation of superoxide dismutase activity; positive regulation of ATPase-coupled calcium transmembrane transporter activity; negative regulation of RNA biosynthetic process; negative regulation of bone development; positive regulation of proteolysis involved in cellular protein catabolic process; negative regulation of calcium-dependent ATPase activity; negative regulation of DNA catabolic process; positive regulation of dUTP diphosphatase activity; negative regulation of leucine-tRNA ligase activity; negative regulation of DNA biosynthetic process	2,22	7,4E-02	2,24	2,2E-01
39	367	387	14	231	238	15	111	150	RCN1P2	reticulocalbin 1 pseudogene 2	13		3,10	4,9E-04	3,14	3,5E-03
222	653	520	1	0	0	0	0	0	LINC02570	long intergenic non-protein coding RNA 2570	6		1,62	1,2E-02	1,25	3,5E-01
157	1035	884	30	4	5	7	4	2	JAKMIP2-AS1	JAKMIP2 antisense RNA 1	5		2,75	2,0E-02	2,44	2,1E-01
5	28	13	1	12	7	5	4	4	AC136469.2	novel transcript	19		2,34	3,2E-02	1,23	9,3E-01
26	91	49	39	51	31	19	37	40	AC006064.4	novel transcript, antisense to GAPDH	12		1,76	4,3E-02	0,87	1,0E+00
119	382	368	229	343	353	440	616	609	MAILR	macrophage interferon regulatory lncRNA	8		1,62	5,3E-02	1,57	2,4E-01
102	327	276	14	43	30	40	42	45	PCCA-DT	PCCA divergent transcript	13		1,67	7,6E-02	1,41	4,9E-01
91	196	226	165	186	163	133	141	122	AP002784.1	novel transcript	11		1,09	8,8E-02	1,29	7,9E-02

DOWNREGULATED GENES

Normalized read counts									GENE	Description	Chr	GO: Biological process	Log_2FC FRDA/CT	FDR	Log_2FC ISO CT/CT	FDR
iPSCs			Developing Neurons			Mature Neurons										
CT	FRDA	ISO CT	CT	FRDA	ISO CT	CT	FRDA	ISO CT								
19	0	0	18	0	0	11	0	0	EIF3CL	eukaryotic translation initiation factor 3 subunit C like	16	translational initiation; formation of cytoplasmic translation initiation complex; cytoplasmic translational initiation; translation	-5,08	1,0E-10	-5,10	1,8E-07
224	5	12	339	602	492	1445	2987	2702	SEZ6L	seizure related 6 homolog like	22	adult locomotory behavior; cerebellar Purkinje cell layer development; synapse maturation; regulation of protein kinase C signaling	-5,24	7,0E-10	-3,95	4,3E-04
609	93	234	1481	1516	1841	1778	1618	1845	ONECUT1	one cut homeobox 1	15	positive regulation of transcription by RNA polymerase II; cell differentiation; regulation of transcription, DNA-templated; liver development; regulation of cell-matrix adhesion; epithelial cell development; glucose metabolic process; regulation of transcription by RNA polymerase II; Notch signaling pathway; endoderm development; anatomical structure morphogenesis; B cell differentiation; positive regulation of cell migration; negative regulation of transforming growth factor	-2,68	1,8E-04	-1,34	6,3E-01

												beta receptor signaling pathway; pancreas development; endocrine pancreas development; cell fate commitment; spleen development; cilium assembly				
**72**	**12**	**25**	21	27	28	64	29	48	**FANK1**	fibronectin type III and ankyrin repeat domains 1	10	negative regulation of apoptotic process; positive regulation of transcription, DNA-templated; regulation of apoptotic process; positive regulation of apoptotic process; positive regulation of DNA-binding transcription factor activity	-2,55	1,4E-03	-1,48	5,2E-01
**4644**	**924**	**1384**	80	73	51	85	68	58	**PDGFA**	platelet derived growth factor subunit A	7	positive regulation of cell population proliferation; platelet-derived growth factor receptor signaling pathway; positive regulation of cell migration; positive regulation of fibroblast proliferation; multicellular organism development; positive regulation of cell division; angiogenesis; actin cytoskeleton organization; MAPK cascade; positive regulation of MAPK cascade; positive regulation of ERK1 and ERK2 cascade; cell-cell signaling; platelet degranulation; positive regulation of protein kinase B signaling; extracellular matrix organization; positive regulation of protein autophosphorylation; animal organ morphogenesis; wound healing; positive regulation of phosphatidylinositol 3-kinase signaling; positive regulation of mesenchymal cell proliferation; lung alveolus development; positive regulation of MAP kinase activity; regulation of peptidyl-tyrosine phosphorylation; response to wounding; negative regulation of platelet activation; regulation of smooth muscle cell migration; negative regulation of phosphatidylinositol biosynthetic process; positive regulation of metanephric mesenchymal cell migration by platelet-derived growth factor receptor-beta signaling pathway; skin development; cell activation; hair follicle development; cell projection assembly; regulation of actin cytoskeleton organization; negative chemotaxis; regulation of branching involved in salivary gland morphogenesis by epithelial-mesenchymal signaling; embryonic lung development; signal transduction	-2,21	1,6E-03	-1,66	1,9E-01
**1073**	**158**	**303**	2462	2542	2727	3189	3120	3318	**EPHB3**	EPH receptor B3	3	protein phosphorylation; transmembrane receptor protein tyrosine kinase signaling pathway; phosphorylation; peptidyl-tyrosine phosphorylation; multicellular organism development; nervous system development; angiogenesis; digestive tract morphogenesis; cell migration; thymus development; ephrin receptor signaling pathway; dendritic spine morphogenesis; protein autophosphorylation; roof of mouth development; axon guidance; positive regulation of synapse assembly; regulation of GTPase activity; positive regulation of kinase activity; regulation of axonogenesis; substrate adhesion-dependent cell spreading; dendritic spine development; regulation of cell-cell adhesion; urogenital system development; axonal fasciculation; corpus callosum development; central nervous system projection neuron axonogenesis; retinal ganglion cell axon guidance	-2,71	2,0E-03	-1,76	4,2E-01
**140**	**67**	**71**	439	285	321	536	390	447	**SCML1**	Scm polycomb group protein like 1	X	negative regulation of transcription, DNA-templated	-1,07	2,0E-03	-0,98	4,3E-02
**122**	**32**	**46**	1562	895	1100	3014	2515	2465	**SCD5**	stearoyl-CoA desaturase 5	4	oxidation-reduction process; lipid metabolic process; fatty acid metabolic process; fatty acid biosynthetic process; unsaturated fatty acid biosynthetic process; fatty-acyl-CoA biosynthetic process; monounsaturated fatty acid biosynthetic process	-1,96	2,7E-03	-1,40	3,2E-01
**714**	**191**	**266**	382	448	370	1284	1040	941	**CERCAM**	cerebral endothelial cell adhesion molecule	9	cell adhesion; leukocyte cell-cell adhesion	-1,88	3,4E-03	-1,39	2,9E-01
**1956**	**247**	**766**	5182	7524	4838	729	1028	857	**CRLF1**	cytokine receptor like factor 1	19	positive regulation of cell population proliferation; positive regulation of tyrosine phosphorylation of STAT protein; cytokine-mediated signaling pathway; negative regulation of neuron apoptotic process; interleukin-27-mediated signaling pathway; ureteric bud development; negative regulation of motor neuron apoptotic process	-2,88	4,8E-03	-1,28	9,4E-01
**2374**	**596**	**1067**	3843	4476	4185	2689	3167	3225	**ZNF516**	zinc finger protein 516	18	regulation of transcription by RNA polymerase II; positive regulation of transcription, DNA-templated; adipose tissue development; regulation of transcription, DNA-templated; positive regulation of cold-induced thermogenesis; brown fat cell differentiation; response to cold	-1,97	6,5E-03	-1,13	7,2E-01
**848**	**380**	**466**	2071	1801	1688	865	895	781	**SCN8A**	sodium voltage-gated channel alpha subunit 8	12	ion transport; transmembrane transport; ion transmembrane transport; cation transmembrane transport; sodium ion transport; regulation of ion transmembrane transport; sodium ion transmembrane transport; nervous system development; neuronal action potential; membrane depolarization during action potential; peripheral nervous system development; myelination	-1,15	1,0E-02	-0,86	4,0E-01
**2730**	**768**	**810**	2683	2380	2065	3120	2897	2643	**MLLT6**	MLLT6, PHD finger containing	17	positive regulation of transcription by RNA polymerase II; regulation of transcription, DNA-templated; renal system process; excretion; positive regulation of sodium ion transport; negative regulation of urine volume; renal sodium excretion; renal potassium excretion; negative regulation of histone H3-K79 methylation	-1,80	1,8E-02	-1,71	1,2E-01
**51**	**9**	**47**	16	16	15	7	8	10	**CPO**	carboxypeptidase O	2	proteolysis	-2,41	2,0E-02	-0,06	1,0E+00
**389**	**123**	**90**	258	178	126	799	554	700	**TMEM145**	transmembrane protein 145	19	G protein-coupled receptor signaling pathway; response to pheromone	-1,63	2,0E-02	-2,06	6,4E-03
**3966**	**1012**	**1478**	5157	6273	8993	7747	7107	7086	**TSPAN18**	tetraspanin 18	11		-1,94	2,2E-02	-1,39	5,6E-01
**4387**	**1889**	**2043**	329	309	303	406	358	337	**TBC1D8**	TBC1 domain family member 8	2	intracellular protein transport; activation of GTPase activity; positive regulation of cell population proliferation; blood circulation; positive regulation of GTPase activity	-1,18	3,2E-02	-1,08	2,5E-01
**1869**	**768**	**1058**	6167	3959	4961	4840	4760	5190	**SEPTIN6**	septin 6	X	cell cycle; cell division; cell differentiation; viral process; spermatogenesis; cilium assembly; cellular protein localization; cytoskeleton-dependent cytokinesis; mitotic cytokinesis	-1,28	3,4E-02	-0,81	7,7E-01
**247**	**92**	**149**	81	56	45	143	108	99	**PCDHAC2**	protocadherin alpha subfamily C, 2	5	homophilic cell adhesion via plasma membrane adhesion molecules; cell adhesion; nervous system development	-1,39	4,0E-02	-0,71	9,8E-01
**1441**	**399**	**583**	964	457	1169	871	734	942	**TSHZ3**	teashirt zinc finger homeobox 3	19	negative regulation of transcription, DNA-templated; regulation of gene expression; multicellular organism development; regulation of transcription by RNA polymerase II; long-term synaptic potentiation; positive regulation of synaptic transmission, glutamatergic; regulation of respiratory gaseous exchange by nervous system process	-1,80	4,3E-02	-1,26	6,8E-01
**468**	**149**	**244**	493	615	616	905	2246	2073	**NTRK3**	neurotrophic receptor tyrosine kinase 3	15	protein phosphorylation; transmembrane receptor protein tyrosine kinase signaling pathway; positive regulation of gene expression; positive regulation of cell population proliferation; positive regulation of cell migration; positive regulation of protein phosphorylation; activation of protein kinase B activity; phosphorylation; peptidyl-tyrosine phosphorylation; multicellular organism development; cell differentiation; nervous system development; neurotrophin signaling pathway; ephrin receptor signaling pathway; heart development; positive regulation of neuron projection development; positive regulation of peptidyl-serine phosphorylation; positive regulation of actin cytoskeleton reorganization; activation of MAPK activity; positive regulation of phosphatidylinositol 3-kinase signaling; activation of GTPase activity; negative regulation of protein phosphorylation; positive regulation of kinase activity; positive regulation of MAPK cascade; regulation of MAPK cascade; regulation of protein kinase B signaling; cellular response to nerve growth factor stimulus; positive regulation of positive	-1,64	4,7E-02	-0,92	9,2E-01

												chemotaxis; positive regulation of phospholipase C activity; positive regulation of neurotrophin TRK receptor signaling pathway; neuron migration; circadian rhythm; modulation by virus of host transcription; neuronal action potential propagation; myelination in peripheral nervous system; mechanoreceptor differentiation; positive regulation of apoptotic process; response to ethanol; neuron fate specification; response to axon injury; positive regulation of axon extension involved in regeneration; negative regulation of astrocyte differentiation; response to corticosterone; positive regulation of synapse assembly; negative regulation of cell death; lens fiber cell differentiation; cellular response to retinoic acid; cochlea development; regulation of postsynaptic density assembly; regulation of presynapse assembly				
**488**	**30**	**223**	17	4	9	23	5	15	**GDA**	guanine deaminase	9	guanine catabolic process; nucleobase-containing compound metabolic process; nervous system development; purine nucleotide catabolic process; guanine metabolic process	-3,45	5,3E-02	-0,87	1,0E+00
**1535**	**635**	**741**	340	546	623	5083	2919	3565	**PTPRM**	protein tyrosine phosphatase receptor type M	18	protein dephosphorylation; dephosphorylation; cell adhesion; signal transduction; peptidyl-tyrosine dephosphorylation; retina layer formation; negative regulation of angiogenesis; negative regulation of endothelial cell proliferation; negative regulation of endothelial cell migration; homophilic cell adhesion via plasma membrane adhesion molecules; neuron projection development; retinal ganglion cell axon guidance; response to drug; positive regulation of blood vessel diameter	-1,26	5,3E-02	-1,03	4,7E-01
**291**	**115**	**232**	7511	7915	6439	3463	4147	3864	**KDM6B**	lysine demethylase 6B	17	histone H3-K27 demethylation; positive regulation of transcription by RNA polymerase II; mesodermal cell differentiation; oxidation-reduction process; chromatin organization; inflammatory response; positive regulation of cold-induced thermogenesis; inflammatory response to antigenic stimulus; chromatin remodeling; regulation of gene expression; response to activity; histone demethylation; hippocampus development; cell fate commitment; endothelial cell differentiation; cardiac muscle cell differentiation; response to fungicide; cellular response to hydrogen peroxide	-1,36	7,3E-02	-0,34	1,0E+00
**2244**	**985**	**876**	218	263	243	512	265	358	**SMAD7**	SMAD family member 7	18	regulation of transcription, DNA-templated; transforming growth factor beta receptor signaling pathway; negative regulation of protein ubiquitination; protein stabilization; negative regulation of cell migration; negative regulation of BMP signaling pathway; negative regulation of DNA-binding transcription factor activity; regulation of ventricular cardiac muscle cell membrane depolarization; ventricular septum morphogenesis; negative regulation of epithelial to mesenchymal transition; negative regulation of transforming growth factor beta receptor signaling pathway; negative regulation of pathway-restricted SMAD protein phosphorylation; positive regulation of transcription by RNA polymerase II; cell differentiation; anatomical structure morphogenesis; protein deubiquitination; BMP signaling pathway; SMAD protein signal transduction; negative regulation of transcription by RNA polymerase II; positive regulation of proteasomal ubiquitin-dependent protein catabolic process; artery morphogenesis; negative regulation of peptidyl-threonine phosphorylation; ureteric bud development; negative regulation of T cell cytokine production; regulation of epithelial to mesenchymal transition; negative regulation of transcription by competitive promoter binding; regulation of transforming growth factor beta receptor signaling pathway; positive regulation of cell-cell adhesion; negative regulation of ossification; positive regulation of protein ubiquitination; regulation of activin receptor signaling pathway; negative regulation of peptidyl-serine phosphorylation; adherens junction assembly; response to laminar fluid shear stress; cellular protein-containing complex localization; negative regulation of ubiquitin-protein transferase activity; ventricular cardiac muscle tissue morphogenesis; regulation of cardiac muscle contraction; pathway-restricted SMAD protein phosphorylation; cellular response to transforming growth factor beta stimulus; negative regulation of chondrocyte proliferation; positive regulation of chondrocyte hypertrophy; cellular response to leukemia inhibitory factor; negative regulation of T-helper 17 type immune response; negative regulation of T-helper 17 cell differentiation	-1,15	8,8E-02	-1,33	1,1E-01
**277**	**92**	**221**	108	31	90	160	46	89	**FXN**	frataxin	9	iron-sulfur cluster assembly; oxidation-reduction process; positive regulation of cell population proliferation; negative regulation of apoptotic process; ion transport; cellular iron ion homeostasis; oxidative phosphorylation; heme biosynthetic process; mitochondrion organization; adult walking behavior; aerobic respiration; embryo development ending in birth or egg hatching; proprioception; negative regulation of multicellular organism growth; negative regulation of organ growth; iron ion homeostasis; positive regulation of cell growth; cellular response to hydrogen peroxide; negative regulation of release of cytochrome c from mitochondria; protein autoprocessing; positive regulation of catalytic activity; small molecule metabolic process; response to iron ion; regulation of ferrochelatase activity; iron incorporation into metallo-sulfur cluster; positive regulation of lyase activity; positive regulation of succinate dehydrogenase activity; positive regulation of aconitate hydratase activity	-1,51	2,4E-01	-0,29	1,0E+00
**33154**	**40**	**35**	21340	34	27	14136	20	41	**AP000763.2**	mitochondrially encoded cytochrome c oxidase I (MT-COI) pseudogene	11		-9,56	5,9E-130	-9,72	2,8E-94
**552**	**3**	**2**	421	2	1	305	1	2	**AC098847.1**	ribosomal protein, large, P0 pseudogene	18		-7,34	3,6E-39	-7,89	2,2E-25
**153**	**1**	**0**	102	2	1	86	1	0	**ZBTB8OSP2**	zinc finger and BTB domain containing 8 opposite strand pseudogene 2	2		-6,88	2,4E-10	-7,54	6,3E-09
**46**	**7**	**9**	114	32	22	109	52	56	**RASA4CP**	RAS p21 protein activator 4C, pseudogene	7		-2,71	2,3E-06	-2,21	3,9E-03
**1051**	**53**	**127**	61	1	1	40	1	1	**RHOXF1-AS1**	RHOXF1 antisense RNA 1	X		-3,94	1,9E-05	-2,79	6,0E-02
**90**	**4**	**5**	62	1	5	50	2	4	**RPL21P28**	ribosomal protein L21 pseudogene 28	1		-4,26	2,9E-05	-3,93	2,6E-03
**598**	**284**	**413**	689	652	808	1239	947	1120	**NNT-AS1**	NNT antisense RNA 1	5		-1,07	1,2E-04	-0,53	6,3E-01
**117**	**18**	**20**	86	14	16	101	10	12	**AC087343.1**	ribosomal protein L21 (Rpl21) pseudogene	8		-2,63	1,7E-04	-2,49	5,5E-03
**44**	**14**	**15**	32	11	8	80	14	16	**AC012184.3**	novel transcript, antisense to DDX19B and DDX19A	16		-1,64	2,0E-03	-1,49	4,5E-02
**60**	**17**	**20**	404	104	98	364	115	126	**AF274858.1**	Morf4 family associated protein 1 (MRFAP1) pseudogene	X		-1,76	2,0E-03	-1,56	6,6E-02

**442**	**84**	**51**	223	36	26	185	31	16	**SORD2P**	sorbitol dehydrogenase 2, pseudogene	15		-2,29	2,4E-03	-2,99	1,7E-04
**121**	**36**	**50**	224	277	213	286	233	184	**AL359881.1**	novel transcript	1		-1,74	2,6E-03	-1,24	2,9E-01
**231**	**106**	**106**	353	178	138	238	135	116	**WASH8P**	WAS protein family homolog 8, pseudogene	12		-1,11	7,8E-03	-1,11	4,6E-02
**167**	**34**	**73**	265	146	153	407	220	244	**LINC02175**	long intergenic non-protein coding RNA 2175	16		-2,24	1,2E-02	-1,16	8,7E-01
**72**	**20**	**27**	3	8	7	5	7	9	**AP003170.1**	interferon stimulated exonuclease gene 20kDa-like 2 (ISG20L2) pseudogene	11		-1,79	2,0E-02	-1,35	4,5E-01
**42**	**11**	**18**	181	126	105	190	124	116	**AL359881.2**	novel transcript	1		-1,84	2,2E-02	-1,19	6,6E-01
**84**	**31**	**35**	92	97	105	116	97	124	**CDC37L1-DT**	CDC37L1 divergent transcript	9		-1,38	2,3E-02	-1,24	2,5E-01
**92**	**34**	**40**	89	32	39	107	31	51	**LYRM4-AS1**	LYRM4 antisense RNA 1	6		-1,41	2,4E-02	-1,16	3,6E-01
**222**	**93**	**111**	12	7	10	20	12	12	**AC022893.1**	novel transcript	8		-1,22	3,0E-02	-0,97	4,4E-01
**57**	**10**	**10**	5	2	2	14	1	1	**AC034102.1**	RAB13 member RAS oncogene family pseudogene	12		-2,41	3,0E-02	-2,42	1,3E-01
**64**	**16**	**41**	1615	1815	1378	411	386	419	**SLC26A10**	solute carrier family 26 member 10	12		-1,96	6,2E-02	-0,65	1,0E+00
**48**	**9**	**6**	149	108	66	16	22	15	**AC084864.2**	novel transcript	7		-2,32	6,5E-02	-2,72	8,3E-02
**128**	**13**	**14**	146	8	3	115	6	7	**AACSP1**	acetoacetyl-CoA synthetase pseudogene 1	5		-2,97	8,0E-02	-2,85	3,1E-01

DIFFERENTIALLY EXPRESSED GENES – DEVELOPING NEURONS

UPREGULATED GENES

Normalized read counts									GENE	Description	Chr	GO: Biological process	Log $_2$ FC FRDA/CT	FDR	Log $_2$ FC ISO CT/CT	FDR
iPSCs			Developing Neurons			Mature Neurons										
CT	FRDA	ISO CT	CT	FRDA	ISO CT	CT	FRDA	ISO CT								
18	42	36	**36**	**3878**	**1143**	8	73	74	**SPON2**	spondin 2	4	cell adhesion; immune system process; innate immune response; mast cell mediated immunity; opsonization; response to lipopolysaccharide; positive regulation of interleukin-6 production; positive regulation of tumor necrosis factor production; defense response to bacterium; induction of bacterial agglutination; defense response to fungus; defense response to virus; positive regulation of macrophage cytokine production; cellular response to lipopolysaccharide	6,38	7,9E-12	4,60	1,4E-04
58	92	110	**20**	**127**	**63**	43	99	81	**MOB3C**	MOB kinase activator 3C	1		2,60	4,2E-08	1,60	5,0E-02
22	40	49	**19**	**69**	**61**	18	48	44	**TSTD3**	thiosulfate sulfurtransferase like domain containing 3	6		1,83	3,0E-07	1,65	1,3E-04
122	192	190	**234**	**533**	**531**	194	500	441	**MINDY1**	MINDY lysine 48 deubiquitinase 1	1	proteolysis; protein K48-linked deubiquitination	1,19	1,8E-06	1,18	4,7E-05
22	21	26	**13**	**329**	**78**	28	38	39	**RASD1**	ras related dexamethasone induced 1	17	signal transduction; G protein-coupled receptor signaling pathway; nitric oxide mediated signal transduction; negative regulation of transcription, DNA-templated	4,29	4,0E-04	2,28	6,8E-01
10886	15191	13263	**8254**	**19031**	**21163**	6392	12889	16069	**RPS23**	ribosomal protein S23	5	translation; cytoplasmic translation; viral transcription; translational initiation; stress granule assembly; nuclear-transcribed mRNA catabolic process, nonsense-mediated decay; SRP-dependent cotranslational protein targeting to membrane; maintenance of translational fidelity	1,20	7,4E-04	1,35	4,8E-04
371	477	336	**4**	**396**	**191**	17	341	188	**COMT**	catechol-O-methyltransferase	22	catecholamine metabolic process; neurotransmitter catabolic process; methylation; developmental process; dopamine metabolic process; catecholamine catabolic process; response to organic cyclic compound; response to drug; short-term memory; cellular response to phosphate starvation; dopamine catabolic process; female pregnancy; learning; estrogen metabolic process; response to lipopolysaccharide; negative regulation of renal sodium excretion; response to estrogen; negative regulation of dopamine metabolic process; response to pain; multicellular organismal reproductive process; negative regulation of smooth muscle cell proliferation; positive regulation of homocysteine metabolic process; regulation of sensory perception of pain	4,98	1,1E-03	4,00	1,1E-01
10	144	122	**15**	**213**	**161**	4	124	88	**PCDHA11**	protocadherin alpha 11	5	homophilic cell adhesion via plasma membrane adhesion molecules; cell adhesion; nervous system development	3,58	1,4E-03	3,15	4,9E-02
20	62	68	**41**	**130**	**118**	32	99	109	**WASHC1**	WASH complex subunit 1	9	Arp2/3 complex-mediated actin nucleation; protein transport; exocytosis; extracellular matrix disassembly; positive regulation of cell migration; negative regulation of autophagy; endosomal transport; positive regulation of pseudopodium assembly; regulation of protein ubiquitination;	1,66	1,8E-03	1,51	3,9E-02

												retrograde transport, endosome to Golgi; negative regulation of phosphatidylinositol 3-kinase activity; retrograde transport, endosome to plasma membrane				
89	115	83	**19**	**337**	**96**	166	121	229	**CALB2**	calbindin 2	16	regulation of cytosolic calcium ion concentration; regulation of presynaptic cytosolic calcium ion concentration; regulation of long-term synaptic potentiation	3,77	1,8E-03	2,04	8,0E-01
5	5	4	**51**	**639**	**174**	10	11	6	**CLDN5**	claudin 5	22	positive regulation of gene expression; negative regulation of gene expression; myelination; response to ethanol; tight junction assembly; negative regulation of cell migration; cell adhesion; bicellular tight junction assembly; outflow tract morphogenesis; cell-cell junction assembly; transforming growth factor beta receptor signaling pathway; learning; positive regulation of cell population proliferation; calcium-independent cell-cell adhesion via plasma membrane cell-adhesion molecules; negative regulation of angiogenesis; positive regulation of protein binding; maintenance of blood-brain barrier; negative regulation of vascular permeability; roof of mouth development; face morphogenesis; positive regulation of establishment of endothelial barrier; positive regulation of bicellular tight junction assembly; establishment of blood-retinal barrier; regulation of bicellular tight junction assembly	3,58	2,1E-03	1,70	9,4E-01
291	292	319	**747**	**1764**	**1117**	608	670	527	**PLK3**	polo like kinase 3	1	protein phosphorylation; cell cycle; phosphorylation; negative regulation of apoptotic process; apoptotic process; cellular response to DNA damage stimulus; negative regulation of transcription by RNA polymerase II; DNA damage response, signal transduction by p53 class mediator resulting in cell cycle arrest; mitotic cell cycle; regulation of cell division; regulation of signal transduction by p53 class mediator; regulation of cytokinesis; G1/S transition of mitotic cell cycle; response to osmotic stress; positive regulation of intracellular protein transport; response to radiation; mitotic cell cycle checkpoint; G2/M transition of mitotic cell cycle; protein kinase B signaling; response to reactive oxygen species; Golgi disassembly; cytoplasmic microtubule organization; mitotic G1/S transition checkpoint; positive regulation of proteasomal ubiquitin-dependent protein catabolic process involved in cellular response to hypoxia; endomitotic cell cycle; positive regulation of chaperone-mediated autophagy	1,24	3,3E-03	0,58	9,8E-01
6	14	17	**5**	**59**	**58**	2	22	20	**PCDHB8**	protocadherin beta 8	5	homophilic cell adhesion via plasma membrane adhesion molecules; cell adhesion	3,26	4,0E-03	3,20	2,8E-02
20	90	92	**105**	**408**	**253**	515	1063	892	**SLC8A1**	solute carrier family 8 member A1	2	transmembrane transport; calcium ion transport; cell communication; regulation of cardiac muscle contraction by regulation of the release of sequestered calcium ion; negative regulation of protein serine/threonine kinase activity; cellular response to reactive oxygen species; regulation of heart rate; ion transport; sodium ion transport; calcium ion transmembrane transport; sodium ion transmembrane transport; calcium ion import across plasma membrane; positive regulation of bone mineralization; muscle contraction; regulation of cardiac conduction; regulation of cell communication by electrical coupling; cardiac muscle contraction; cellular response to caffeine; calcium ion import; response to muscle stretch; calcium ion homeostasis; cellular calcium ion homeostasis; membrane depolarization during cardiac muscle cell action potential; cellular sodium ion homeostasis; sodium ion export across plasma membrane; relaxation of cardiac muscle; cell communication by electrical coupling involved in cardiac conduction; regulation of cardiac muscle contraction by calcium ion signaling; regulation of gene expression; regulation of the force of heart contraction; sodium ion import across plasma membrane; positive regulation of the force of heart contraction; vascular associated smooth muscle contraction; metal ion transport; relaxation of smooth muscle; negative regulation of cytosolic calcium ion concentration; cardiac muscle cell development; cytosolic calcium ion transport; calcium ion transport into cytosol; regulation of postsynaptic cytosolic calcium ion concentration; response to hypoxia; regulation of sodium ion transport; positive regulation of cytosolic calcium ion concentration; response to nutrient; response to glucose; positive regulation of fibroblast migration; telencephalon development; response to ATP; response to immobilization stress; response to drug; response to hydrogen peroxide; regulation of calcium ion transport; cellular response to cAMP; cellular response to hypoxia; calcium ion export	1,92	4,1E-03	1,23	6,1E-01
106	164	117	**17**	**131**	**55**	20	19	25	**TESC**	tescalcin	12	protein transport; cell differentiation; negative regulation of catalytic activity; positive regulation of transcription, DNA-templated; negative regulation of protein kinase activity; protein stabilization; positive regulation of gene expression; negative regulation of cell population proliferation; protein maturation; cellular response to retinoic acid; regulation of cell adhesion mediated by integrin; protein localization to plasma membrane; positive regulation of sodium:proton antiporter activity; positive regulation of megakaryocyte differentiation; positive regulation of granulocyte differentiation	2,77	4,4E-03	1,53	8,2E-01
808	3610	3892	**7**	**156**	**63**	46	24	51	**APELA**	apelin receptor early endogenous ligand	4	multicellular organism development; cell differentiation; angiogenesis; heart development; positive regulation of ERK1 and ERK2 cascade; positive regulation of angiogenesis; vasculogenesis; gastrulation; endoderm development; mesoderm migration involved in gastrulation; adult heart development; embryonic heart tube development; positive regulation of heart contraction; apelin receptor signaling pathway; placenta blood vessel development; coronary vasculature development; mesendoderm migration; cell migration involved in mesendoderm migration; positive regulation of trophoblast cell migration; positive regulation of blood vessel endothelial cell proliferation involved in sprouting angiogenesis; positive regulation of G protein-coupled receptor internalization	3,72	5,4E-03	2,52	5,4E-01
14	23	38	**54**	**1074**	**203**	2	45	22	**OTX1**	orthodenticle homeobox 1	2	regulation of transcription, DNA-templated; multicellular organism development; positive regulation of transcription by RNA polymerase II; regulation of transcription by RNA polymerase II; anterior/posterior pattern specification; metencephalon development; forebrain development; midbrain development; inner ear morphogenesis; diencephalon morphogenesis	4,09	5,4E-03	1,74	1,0E+00
7	19	20	**9**	**156**	**42**	2	8	27	**CDH15**	cadherin 15	16	homophilic cell adhesion via plasma membrane adhesion molecules; cell adhesion; cell-cell adhesion; positive regulation of muscle cell differentiation; adherens junction organization; cell-cell adhesion via plasma-membrane adhesion molecules	3,86	6,5E-03	2,01	9,2E-01
65	136	120	**31**	**312**	**152**	17	44	44	**ARC**	activity regulated cytoskeleton associated protein	8	mRNA transport; multicellular organism development; endocytosis; cytoskeleton organization; cell migration; protein homooligomerization; regulation of cell morphogenesis; long-term memory; regulation of long-term synaptic potentiation; regulation of neuronal synaptic plasticity; modulation of chemical synaptic transmission; long-term synaptic potentiation; endoderm development; learning; anterior/posterior pattern specification; dendritic spine morphogenesis; regulation of dendritic spine morphogenesis; regulation of postsynaptic neurotransmitter receptor internalization; vesicle-mediated intercellular transport; regulation of long-term synaptic depression; positive regulation of AMPA receptor activity	3,12	8,0E-03	2,10	6,1E-01
909	861	1004	**115**	**442**	**422**	457	616	601	**KHNYN**	KH and NYN domain containing	14	RNA phosphodiester bond hydrolysis, endonucleolytic	1,87	9,5E-03	1,81	7,1E-02
18	41	26	**97**	**862**	**165**	83	232	158	**C4orf48**	chromosome 4 open reading frame 48	4		3,04	9,5E-03	0,72	1,0E+00
20	2624	2895	**20**	**37489**	**38283**	25	15558	15743	**NNAT**	neuronatin	20	brain development; multicellular organism development; positive regulation of insulin secretion; protein lipoylation	5,42	1,1E-02	5,23	5,9E-02

81	386	341	**3**	**118**	**49**	9	117	71	**TXNRD2**	thioredoxin reductase 2	22	oxidation-reduction process; cell redox homeostasis; cellular oxidant detoxification; cellular response to oxidative stress; response to oxygen radical	4,22	1,2E-02	3,07	5,1E-01
97	122	131	**46**	**115**	**71**	177	210	181	**HBEGF**	heparin binding EGF like growth factor	5	positive regulation of protein kinase B signaling; positive regulation of cell migration; MAPK cascade; signal transduction; membrane organization; positive regulation of cell population proliferation; cell chemotaxis; ERBB2 signaling pathway; regulation of cell motility; muscle organ development; epidermal growth factor receptor signaling pathway; negative regulation of epidermal growth factor receptor signaling pathway; positive regulation of epidermal growth factor-activated receptor activity; positive regulation of wound healing; regulation of heart contraction; cell migration; positive regulation of cell growth; wound healing, spreading of epidermal cells; positive regulation of smooth muscle cell proliferation; positive regulation of peptidyl-tyrosine phosphorylation; negative regulation of elastin biosynthetic process; positive regulation of keratinocyte migration	1,30	1,5E-02	0,61	1,0E+00
31	92	85	**30**	**133**	**157**	12	91	94	**PCDHGA10**	protocadherin gamma subfamily A, 10	5	homophilic cell adhesion via plasma membrane adhesion molecules; cell adhesion	2,09	1,7E-02	2,30	2,8E-02
210	226	260	**230**	**1292**	**364**	46	57	76	**CCDC88B**	coiled-coil domain containing 88B	11	cytoskeleton-dependent intracellular transport; positive regulation of cytokine production; cytoplasmic microtubule organization; positive regulation of T cell proliferation; defense response to protozoan; positive regulation of T cell activation	2,44	1,7E-02	0,64	1,0E+00
24	43	37	**10**	**37**	**27**	15	37	41	**PEX11G**	peroxisomal biogenesis factor 11 gamma	19	peroxisome fission; regulation of peroxisome size	1,81	1,8E-02	1,34	5,5E-01
9	25	26	**34**	**381**	**133**	14	29	21	**SUSD2**	sushi domain containing 2	22	negative regulation of cell division; negative regulation of cell cycle G1/S phase transition	3,32	1,8E-02	1,82	9,5E-01
94	149	302	**1849**	**8242**	**4072**	432	438	464	**DLL3**	delta like canonical Notch ligand 3	19	multicellular organism development; cell differentiation; Notch signaling pathway; skeletal system development; somitogenesis; compartment pattern specification; tissue development; paraxial mesoderm development; negative regulation of neurogenesis	2,16	2,0E-02	1,13	9,9E-01
220	228	255	**818**	**1999**	**1185**	319	379	345	**GADD45A**	growth arrest and DNA damage inducible alpha	1	regulation of cell cycle; cell cycle; cellular response to DNA damage stimulus; cell cycle arrest; DNA damage response, signal transduction by p53 class mediator resulting in cell cycle arrest; positive regulation of apoptotic process; cellular response to mechanical stimulus; apoptotic process; positive regulation of JNK cascade; DNA repair; positive regulation of p38MAPK cascade; regulation of cyclin-dependent protein serine/threonine kinase activity; activation of MAPKKK activity; negative regulation of transcription by RNA polymerase II; positive regulation of reactive oxygen species metabolic process; signal transduction in response to DNA damage; cellular response to ionizing radiation; negative regulation of blood vessel endothelial cell migration; negative regulation of protein kinase activity; centrosome cycle; negative regulation of angiogenesis; negative regulation of peptidyl-serine phosphorylation of STAT protein; mitotic cell cycle arrest; negative regulation of protein serine/threonine kinase activity	1,29	2,1E-02	0,53	1,0E+00
124	163	185	**24**	**183**	**145**	155	166	143	**PSTPIP2**	proline-serine-threonine phosphatase interacting protein 2	18	cytoskeleton organization; cell migration; actin filament polymerization	2,72	2,1E-02	2,39	2,6E-01
142	159	182	**1163**	**4388**	**2280**	623	947	938	**SEPTIN4**	septin 4	17	cell cycle; apoptotic process; cell division; cell differentiation; spermatogenesis; positive regulation of apoptotic process; positive regulation of protein ubiquitination; cellular protein localization; cytoskeleton-dependent cytokinesis; regulation of apoptotic process; positive regulation of intrinsic apoptotic signaling pathway; regulation of exocytosis	1,90	2,1E-02	0,96	1,0E+00
449	458	571	**746**	**1641**	**1213**	388	299	336	**TOB1**	transducer of ERBB2, 1	17	negative regulation of cell population proliferation; negative regulation of translation; negative regulation of nucleic acid-templated transcription; regulation of gene expression; positive regulation of nuclear-transcribed mRNA poly(A) tail shortening; positive regulation of nuclear-transcribed mRNA catabolic process, deadenylation-dependent decay; negative regulation of BMP signaling pathway; negative regulation of osteoblast differentiation; negative regulation of nuclear-transcribed mRNA poly(A) tail shortening; regulation of SMAD protein signal transduction	1,13	2,3E-02	0,70	8,6E-01
38	48	47	**69**	**203**	**100**	70	125	94	**PBX4**	PBX homeobox 4	19	regulation of transcription, DNA-templated; positive regulation of transcription, DNA-templated; regulation of transcription by RNA polymerase II; animal organ morphogenesis; eye development; brain development; embryonic organ development; neuron development	1,54	2,6E-02	0,52	1,0E+00
58	71	44	**57**	**202**	**71**	124	145	119	**ADGRB1**	adhesion G protein-coupled receptor B1	8	G protein-coupled receptor signaling pathway; cell surface receptor signaling pathway; negative regulation of angiogenesis; muscle organ development; signal transduction; immune system process; innate immune response; nervous system development; phagocytosis; defense response to Gram-negative bacterium; cell adhesion; negative regulation of endothelial cell migration; phagocytosis, recognition; phagocytosis, engulfment; adenylate cyclase-activating G protein-coupled receptor signaling pathway; axonogenesis; peripheral nervous system development; negative regulation of cell population proliferation; negative regulation of protein ubiquitination; negative regulation of protein catabolic process; apoptotic cell clearance; engulfment of apoptotic cell; regulation of synaptic plasticity; positive regulation of synapse assembly; positive regulation of myoblast fusion; positive regulation of reactive oxygen species biosynthetic process	1,77	2,6E-02	0,29	1,0E+00
118	275	186	**68**	**350**	**119**	63	132	79	**RTN4R**	reticulon 4 receptor	22	positive regulation of GTPase activity; negative regulation of neuron projection development; negative regulation of axon regeneration; cell surface receptor signaling pathway; positive regulation of Rho protein signal transduction; negative regulation of axonogenesis; negative regulation of axon extension; corpus callosum development; neuronal signal transduction; axonogenesis	2,27	2,6E-02	0,75	1,0E+00
67	74	74	**32**	**80**	**38**	20	32	27	**TBC1D2**	TBC1 domain family member 2	9	intracellular protein transport; activation of GTPase activity; positive regulation of GTPase activity	1,30	2,8E-02	0,23	1,0E+00
56	126	86	**40**	**320**	**45**	26	39	42	**CCDC85B**	coiled-coil domain containing 85B	11	cell differentiation; viral process; regulation of growth; negative regulation of cell growth; negative regulation of transcription, DNA-templated; negative regulation of fat cell differentiation	2,85	2,9E-02	0,14	1,0E+00
379	431	442	**735**	**2249**	**1694**	1081	654	571	**PRSS23**	serine protease 23	11	proteolysis; cellular protein metabolic process; post-translational protein modification	1,59	3,3E-02	1,18	6,5E-01
303	598	485	**491**	**1643**	**881**	513	867	842	**ZFR2**	zinc finger RNA binding protein 2	19		1,71	4,0E-02	0,82	1,0E+00
1	3	5	**6**	**63**	**20**	6	2	2	**MYL4**	myosin light chain 4	17	cardiac muscle contraction; muscle filament sliding; positive regulation of ATPase activity; regulation of the force of heart contraction	3,34	4,0E-02	1,69	1,0E+00
347	674	424	**239**	**1561**	**522**	135	249	179	**ELN**	elastin	7	outflow tract morphogenesis; extracellular matrix organization; animal organ morphogenesis; aortic valve morphogenesis; respiratory gaseous exchange by respiratory system; blood circulation; regulation of smooth muscle cell proliferation; skeletal muscle tissue development; regulation of actin filament polymerization; stress fiber assembly	2,58	4,0E-02	1,04	1,0E+00

215	264	235	**2692**	**6727**	**4441**	1012	1428	1277	**DISP3**	dispatched RND transporter family member 3	1	lipid metabolic process; cell differentiation; steroid metabolic process; cholesterol metabolic process; smoothened signaling pathway; positive regulation of neural precursor cell proliferation; negative regulation of neuron differentiation; cholesterol homeostasis; regulation of lipid transport; positive regulation of lipid metabolic process	1,33	4,1E-02	0,72	1,0E+00
75	149	113	**45**	**218**	**77**	73	91	98	**TMEM160**	transmembrane protein 160	19		2,18	4,1E-02	0,72	1,0E+00
34	98	77	**136**	**599**	**164**	71	157	105	**ANTKMT**	adenine nucleotide translocase lysine methyltransferase	16	methylation; peptidyl-lysine methylation; peptidyl-lysine trimethylation; positive regulation of proton-transporting ATP synthase activity, rotational mechanism; regulation of mitochondrial ATP synthesis coupled proton transport	2,09	4,3E-02	0,26	1,0E+00
12	9	15	**33**	**198**	**90**	59	48	26	**PLEKHD1**	pleckstrin homology and coiled-coil domain containing D1	14		2,51	4,6E-02	1,38	1,0E+00
1489	740	646	**630**	**3086**	**1508**	1557	933	817	**HES6**	hes family bHLH transcription factor 6	2	regulation of transcription, DNA-templated; negative regulation of DNA-binding transcription factor activity; multicellular organism development; cell differentiation; regulation of transcription by RNA polymerase II; anterior/posterior pattern specification; regulation of neurogenesis; negative regulation of transcription by RNA polymerase II; nervous system development	2,20	4,8E-02	1,19	1,0E+00
417	305	314	**184**	**764**	**350**	256	228	224	**LTBP3**	latent transforming growth factor beta binding protein 3	11	positive regulation of mesenchymal stem cell differentiation; elastic fiber assembly; transforming growth factor beta activation; positive regulation of mesenchymal stem cell proliferation; skeletal system development; transforming growth factor beta receptor signaling pathway; negative regulation of bone mineralization; negative regulation of chondrocyte differentiation; positive regulation of bone resorption; bone remodeling; bone morphogenesis; lung saccule development	1,99	4,8E-02	0,88	1,0E+00
5897	6188	5744	**1884**	**4484**	**3851**	2652	4023	3034	**CLIC1**	chloride intracellular channel 1	6	chloride transport; regulation of cell cycle; ion transport; ion transmembrane transport; regulation of ion transmembrane transport; signal transduction; chloride transmembrane transport; platelet aggregation; positive regulation of osteoblast differentiation; regulation of mitochondrial membrane potential	1,23	5,3E-02	1,01	5,9E-01
328	1210	574	**646**	**1364**	**1120**	568	741	640	**ATF5**	activating transcription factor 5	19	regulation of transcription, DNA-templated; regulation of transcription by RNA polymerase II; negative regulation of apoptotic process; positive regulation of transcription, DNA-templated; fat cell differentiation; negative regulation of cell population proliferation; positive regulation of transcription by RNA polymerase II; negative regulation of transcription, DNA-templated; regulation of centrosome cycle; negative regulation of cell cycle G2/M phase transition; cerebellar granule cell precursor proliferation; circadian rhythm; post-embryonic development; regulation of gene expression; olfactory bulb interneuron differentiation; olfactory bulb interneuron development; olfactory lobe development; multicellular organism growth	1,07	5,4E-02	0,79	7,8E-01
541	576	433	**23**	**161**	**72**	23	104	84	**SLC22A31**	solute carrier family 22 member 31	16	transmembrane transport; ion transport	2,55	5,6E-02	1,45	1,0E+00
0	0	0	**16**	**109**	**64**	1	1	3	**AGBL1**	ATP/GTP binding protein like 1	15	proteolysis; protein side chain deglutamylation; C-terminal protein deglutamylation	2,82	5,8E-02	1,97	8,2E-01
298	525	509	**1431**	**6026**	**2888**	988	1361	1236	**PDE2A**	phosphodiesterase 2A	11	signal transduction; adenylate cyclase-inhibiting G protein-coupled receptor signaling pathway; negative regulation of transcription by RNA polymerase II; positive regulation of inflammatory response; G protein-coupled receptor signaling pathway; cellular response to mechanical stimulus; positive regulation of gene expression; heart valve development; ventricular septum development; aorta development; regulation of cGMP-mediated signaling; negative regulation of cGMP-mediated signaling; regulation of mitochondrion organization; cAMP-mediated signaling; cGMP-mediated signaling; cellular response to macrophage colony-stimulating factor stimulus; negative regulation of vascular permeability; positive regulation of vascular permeability; regulation of cAMP-mediated signaling; negative regulation of cAMP-mediated signaling; cGMP catabolic process; establishment of endothelial barrier; cellular response to cAMP; cellular response to cGMP; cellular response to transforming growth factor beta stimulus; cellular response to 2,3,7,8-tetrachlorodibenzodioxine; monocyte differentiation; cellular response to drug; cellular response to granulocyte macrophage colony-stimulating factor stimulus	2,04	6,7E-02	0,98	1,0E+00
77	58	94	**363**	**1704**	**640**	166	142	132	**GADD45G**	growth arrest and DNA damage inducible gamma	9	regulation of cell cycle; apoptotic process; multicellular organism development; cell differentiation; positive regulation of apoptotic process; positive regulation of cold-induced thermogenesis; positive regulation of JNK cascade; positive regulation of p38MAPK cascade; activation of MAPKKK activity	2,20	6,7E-02	0,80	1,0E+00
43	46	49	**24**	**70**	**44**	10	8	8	**CLCN1**	chloride voltage-gated channel 1	7	transmembrane transport; chloride transport; chloride transmembrane transport; ion transport; regulation of ion transmembrane transport; ion transmembrane transport; muscle contraction; neuronal action potential propagation	1,53	7,3E-02	0,86	1,0E+00
1418	2773	2640	**218**	**455**	**344**	295	427	442	**CBR1**	carbonyl reductase 1	21	oxidation-reduction process; epithelial cell differentiation; cyclooxygenase pathway; drug metabolic process; vitamin K metabolic process	1,04	7,7E-02	0,64	9,8E-01
148	104	123	**843**	**2328**	**1179**	234	228	231	**FAM110A**	family with sequence similarity 110 member A	20		1,47	7,7E-02	0,49	1,0E+00
392	525	491	**28**	**100**	**51**	48	70	51	**EGFL7**	EGF like domain multiple 7	9	cell adhesion; multicellular organism development; cell differentiation; angiogenesis; anatomical structure development; blood vessel development; vasculogenesis; positive regulation of endothelial cell proliferation; negative regulation of Notch signaling pathway	1,76	7,9E-02	0,80	1,0E+00
268	478	464	**55**	**223**	**74**	40	63	56	**ADGRE5**	adhesion G protein-coupled receptor E5	19	G protein-coupled receptor signaling pathway; cell surface receptor signaling pathway; cell adhesion; signal transduction; neutrophil degranulation; inflammatory response; immune response; cell-cell signaling; adenylate cyclase-activating G protein-coupled receptor signaling pathway	1,92	8,6E-02	0,38	1,0E+00
4	3	4	**54**	**389**	**49**	2	5	1	**LCNL1**	lipocalin like 1	9		2,88	9,4E-02	-0,02	1,0E+00
4802	5234	5996	**449**	**3425**	**3067**	1416	492	439	**PDPN**	podoplanin	1	negative regulation of apoptotic process; multicellular organism development; amino acid transmembrane transport; negative regulation of cell population proliferation; positive regulation of cell migration; Rho protein signal transduction; regulation of cell shape; cell-cell adhesion; platelet activation; positive regulation of epithelial to mesenchymal transition; wound healing, spreading of cells; positive regulation of cellular component movement; folic acid transport; positive regulation of extracellular matrix disassembly; cell morphogenesis; lung development; regulation of lamellipodium morphogenesis; lymphangiogenesis; lymph node development; actin-mediated cell contraction; regulation of substrate adhesion-dependent cell spreading; positive regulation of platelet aggregation; water transport; tube morphogenesis; response to hyperoxia; lymphatic endothelial cell fate commitment; regulation of myofibroblast contraction; invadopodium organization	2,65	9,5E-02	2,48	5,0E-01
2	427	451	**4**	**538**	**707**	5	286	468	**RPS4XP22**	ribosomal protein S4X pseudogene 22	19		6,84	9,1E-27	7,22	1,8E-28

57	86	98	**48**	**147**	**142**	68	109	130	**ELOA-AS1**	ELOA antisense RNA 1	1		1,60	3,3E-07	1,55	1,8E-05
39	367	387	**14**	**231**	**238**	15	111	150	**RCN1P2**	reticulocalbin 1 pseudogene 2	13		3,77	9,3E-06	3,79	1,0E-04
4	3	6	**6**	**99**	**24**	5	7	13	**LINC02293**	long intergenic non-protein coding RNA 2293	14		3,84	4,3E-04	1,80	8,6E-01
0	1	2	**15**	**196**	**80**	10	5	9	**AC025048.1**	novel transcript	17		3,64	4,3E-04	2,31	4,0E-01
217	561	532	**452**	**1997**	**1150**	252	914	704	**MUC20-OT1**	MUC20 overlapping transcript	3		2,11	4,5E-04	1,32	4,7E-01
0	1	3	**83**	**751**	**389**	4	3	2	**AP003049.2**	novel transcript, antisense to C11orf87	11		3,21	1,2E-02	2,18	6,4E-01
32	35	29	**13**	**127**	**23**	11	9	4	**ESPNP**	espin pseudogene	1		3,13	1,5E-02	0,77	1,0E+00
131	199	207	**210**	**603**	**525**	483	1245	1220	**LINC00632**	long intergenic non-protein coding RNA 632	X		1,50	1,8E-02	1,30	2,8E-01
72	135	203	**189**	**496**	**381**	208	361	357	**AL109918.1**	heterogeneous nuclear ribonucleoprotein A/B pseudogene	6		1,38	2,2E-02	1,00	6,4E-01
17	46	50	**45**	**212**	**165**	465	602	558	**MAGI2-AS3**	MAGI2 antisense RNA 3	7		2,12	3,9E-02	1,76	5,0E-01
7	17	18	**13**	**134**	**42**	2	1	5	**AC020909.2**	novel transcript	19		3,20	3,9E-02	1,53	1,0E+00
43	166	124	**210**	**1927**	**1392**	34	441	160	**H19**	H19 imprinted maternally expressed transcript	11		3,04	4,1E-02	2,51	5,1E-01
81	167	153	**134**	**504**	**332**	60	123	129	**MIR570**	microRNA 570	3		1,87	4,5E-02	1,27	8,2E-01
260	293	217	**340**	**885**	**494**	224	325	281	**AP002026.1**	novel transcript, antisense to ADH4, ADH6 & ADH1A	4		1,37	4,6E-02	0,53	1,0E+00
48	49	54	**53**	**114**	**109**	54	62	61	**AC005229.4**	novel transcript, antisense to CUL1	7		1,08	7,5E-02	1,02	4,3E-01
11	7	6	**14**	**71**	**43**	9	4	3	**LINC01686**	long intergenic non-protein coding RNA 1686	1		2,32	7,8E-02	1,57	9,1E-01

DOWNREGULATED GENES

Normalized read counts									GENE	Description	Chr	GO: Biological process	Log_2FC FRDA/CT	FDR	Log_2FC ISO CT/CT	FDR
iPSCs			Developing Neurons			Mature Neurons										
CT	FRDA	ISO CT	CT	FRDA	ISO CT	CT	FRDA	ISO CT								
88	64	65	**348**	**56**	**65**	255	84	102	**SAXO2**	stabilizer of axonemal microtubules 2	15	microtubule anchoring	-2,61	5,9E-13	-2,38	4,8E-08
19	0	0	**18**	**0**	**0**	11	0	0	**EIF3CL**	eukaryotic translation initiation factor 3 subunit C like	16	translational initiation; formation of cytoplasmic translation initiation complex; cytoplasmic translational initiation; translation	-5,07	9,7E-11	-6,21	5,8E-07
210	100	85	**196**	**90**	**120**	236	103	128	**THNSL1**	threonine synthase like 1	10		-1,11	1,2E-03	-0,70	5,1E-01
669	465	626	**391**	**80**	**101**	482	340	465	**SYT6**	synaptotagmin 6	1	acrosome reaction; vesicle-mediated transport; regulation of dopamine secretion; calcium-ion regulated exocytosis; regulation of calcium ion-dependent exocytosis; calcium ion-regulated exocytosis of neurotransmitter; cellular response to calcium ion; acrosomal vesicle exocytosis; presynaptic dense core vesicle exocytosis	-2,24	2,5E-03	-1,90	1,1E-01
27723	30500	32082	**21182**	**10009**	**10902**	13127	14531	12828	**ITM2C**	integral membrane protein 2C	2	neuron differentiation; negative regulation of neuron projection development; negative regulation of amyloid precursor protein biosynthetic process; positive regulation of extrinsic apoptotic signaling pathway	-1,08	3,2E-03	-0,95	8,4E-02
1116	1231	1546	**1489**	**219**	**194**	2944	3264	2273	**TSPAN2**	tetraspanin 2	1	inflammatory response; brain development; astrocyte development; microglia development; myelination; oligodendrocyte differentiation; axon development	-2,68	5,6E-03	-2,80	2,0E-02
1934	1318	1418	**348**	**104**	**101**	431	204	162	**GABRA5**	gamma-aminobutyric acid type A receptor subunit alpha5	15	ion transport; chloride transport; gamma-aminobutyric acid signaling pathway; ion transmembrane transport; signal transduction; chemical synaptic transmission; regulation of membrane potential; nervous system process; chloride transmembrane transport; synaptic transmission, GABAergic; regulation of postsynaptic membrane potential; behavioral fear response; brain development; sensory perception of sound; associative learning; regulation of neuron apoptotic process; negative regulation of neuron apoptotic process; neuron development; inner ear receptor cell development; innervation; cochlea development	-1,72	6,5E-03	-1,75	3,4E-02

588	572	837	**45**	**8**	**15**	4	1	3	**COBL**	cordon-bleu WH2 repeat protein	7	digestive tract development; embryonic axis specification; neural tube closure; actin filament polymerization; somite specification; liver development; notochord development; floor plate development; collateral sprouting in absence of injury; actin filament network formation; positive regulation of dendrite development; actin cytoskeleton organization; positive regulation of ruffle assembly	-2,41	7,9E-03	-1,53	6,8E-01
2189	2598	2261	**429**	**72**	**113**	110	32	94	**TUBA4A**	tubulin alpha 4a	2	microtubule-based process; microtubule cytoskeleton organization; mitotic cell cycle; G2/M transition of mitotic cell cycle; regulation of G2/M transition of mitotic cell cycle; ciliary basal body-plasma membrane docking; platelet degranulation; cytoskeleton organization	-2,49	8,0E-03	-1,84	4,6E-01
284	164	191	**44**	**12**	**8**	67	29	24	**STAG3**	stromal antigen 3	7	cell cycle; chromosome segregation; meiotic cell cycle; sister chromatid cohesion; synaptonemal complex assembly; synapsis; protein localization to chromosome	-1,84	1,7E-02	-2,34	1,1E-02
1	1	0	**353**	**64**	**81**	207	283	195	**GLRA1**	glycine receptor alpha 1	5	ion transport; chloride transport; ion transmembrane transport; neuropeptide signaling pathway; signal transduction; excitatory postsynaptic potential; cellular response to amino acid stimulus; chemical synaptic transmission; regulation of membrane potential; nervous system process; chloride transmembrane transport; positive regulation of acrosome reaction; response to amino acid; synaptic transmission, glycinergic; cellular response to zinc ion; muscle contraction; startle response; negative regulation of transmission of nerve impulse; inhibitory postsynaptic potential; cellular response to ethanol; response to alcohol; action potential; regulation of respiratory gaseous exchange by nervous system process; anion transport; acrosome reaction; visual perception; adult walking behavior; regulation of respiratory gaseous exchange; neuromuscular process controlling posture; neuromuscular process; righting reflex	-2,30	1,8E-02	-2,00	2,7E-01
31	14	28	**166**	**31**	**26**	129	51	65	**GRIN1**	glutamate ionotropic receptor NMDA type subunit 1	9	ion transport; positive regulation of reactive oxygen species biosynthetic process; chemical synaptic transmission; regulation of membrane potential; positive regulation of transcription by RNA polymerase II; MAPK cascade; ephrin receptor signaling pathway; brain development; calcium-mediated signaling; regulation of synaptic plasticity; calcium ion transmembrane import into cytosol; excitatory chemical synaptic transmission; visual learning; protein heterotetramerization; ionotropic glutamate receptor signaling pathway; calcium ion homeostasis; response to ethanol; positive regulation of cysteine-type endopeptidase activity; regulation of NMDA receptor activity; cation transport; excitatory postsynaptic potential; positive regulation of excitatory postsynaptic potential; positive regulation of calcium ion transport into cytosol; propylene metabolic process; response to glycine; conditioned taste aversion; startle response; suckling behavior; response to amphetamine; calcium ion transport; cellular calcium ion homeostasis; learning or memory; learning; memory; long-term memory; associative learning; adult locomotory behavior; olfactory learning; regulation of cell communication; sensory perception of pain; pons maturation; cerebral cortex development; social behavior; synaptic transmission, glutamatergic; positive regulation of apoptotic process; response to morphine; regulation of neuron apoptotic process; negative regulation of neuron apoptotic process; regulation of respiratory gaseous exchange; regulation of neuronal synaptic plasticity; regulation of long-term neuronal synaptic plasticity; regulation of dendrite morphogenesis; regulation of axonogenesis; neuromuscular process; regulation of synapse assembly; prepulse inhibition; male mating behavior; protein localization to postsynaptic membrane; ion transmembrane transport	-2,29	2,2E-02	-2,53	3,9E-02
2	1	1	**283**	**76**	**97**	249	207	205	**GOLGA7B**	golgin A7 family member B	10	protein targeting to membrane; peptidyl-L-cysteine S-palmitoylation	-1,81	2,8E-02	-1,47	5,0E-01
8433	8351	7939	**75**	**28**	**39**	156	30	47	**EPCAM**	epithelial cell adhesion molecule	2	ureteric bud development; negative regulation of apoptotic process; cell-cell adhesion via plasma-membrane adhesion molecules; positive regulation of cell motility; leukocyte migration; positive regulation of transcription by RNA polymerase II; stem cell differentiation; positive regulation of cell population proliferation; positive regulation of stem cell proliferation; signal transduction involved in regulation of gene expression; negative regulation of cell-cell adhesion mediated by cadherin	-1,43	3,1E-02	-0,93	8,2E-01
1897	1552	1820	**163**	**32**	**37**	114	63	54	**KCNS3**	potassium voltage-gated channel modifier subfamily S member 3	2	ion transport; transmembrane transport; potassium ion transport; protein homooligomerization; regulation of ion transmembrane transport; potassium ion transmembrane transport; regulation of insulin secretion; ion transmembrane transport	-2,30	3,3E-02	-2,04	3,5E-01
4304	6165	5752	**322**	**120**	**171**	268	143	231	**GPR176**	G protein-coupled receptor 176	15	G protein-coupled receptor signaling pathway; signal transduction; rhythmic process; circadian behavior; chemical synaptic transmission; adenylate cyclase-inhibiting G protein-coupled receptor signaling pathway	-1,42	4,0E-02	-0,90	8,9E-01
2	2	4	**97**	**17**	**36**	50	33	47	**TCTE1**	t-complex-associated-testis-expressed 1	6	flagellated sperm motility	-2,37	4,5E-02	-1,31	1,0E+00
10188	10035	9391	**1603**	**613**	**799**	3064	1699	1654	**KIF1C**	kinesin family member 1C	17	microtubule-based movement; vesicle-mediated transport; cytoskeleton-dependent intracellular transport; retrograde vesicle-mediated transport, Golgi to endoplasmic reticulum; anterograde neuronal dense core vesicle transport; retrograde neuronal dense core vesicle transport	-1,38	4,5E-02	-0,99	7,8E-01
5648	5579	5380	**1526**	**513**	**432**	1292	932	911	**FBLIM1**	filamin binding LIM protein 1	1	cell adhesion; regulation of cell shape; cell-cell adhesion; regulation of integrin activation; cell junction assembly	-1,55	5,4E-02	-1,78	7,8E-02
9	9	13	**244**	**47**	**25**	41	33	13	**DKK 1,00**	dickkopf WNT signaling pathway inhibitor 1	10	multicellular organism development; negative regulation of Wnt signaling pathway; positive regulation of gene expression; negative regulation of cardiac muscle cell differentiation; negative regulation of canonical Wnt signaling pathway; positive regulation of tau-protein kinase activity; negative regulation of pathway-restricted SMAD protein phosphorylation; negative regulation of apoptotic process; Wnt signaling pathway; negative regulation of signaling receptor activity; negative regulation of transcription by RNA polymerase II; cell morphogenesis involved in differentiation; endoderm formation; mesoderm formation; hair follicle development; endoderm development; learning or memory; negative regulation of neuron projection development; regulation of Wnt signaling pathway; negative regulation of ossification; embryonic limb morphogenesis; negative regulation of BMP signaling pathway; forebrain development; response to retinoic acid; regulation of synapse organization; regulation of synaptic transmission, glutamatergic; limb development; head morphogenesis; face morphogenesis; motor learning; positive regulation of heart induction by negative regulation of canonical Wnt signaling pathway; Wnt signaling pathway involved in somitogenesis; modulation of age-related behavioral decline; positive regulation of midbrain dopaminergic neuron differentiation; negative regulation of presynapse assembly; negative regulation of peptidyl-serine phosphorylation; positive regulation of neuron death; positive regulation of cell death; regulation of receptor internalization; negative regulation of protein binding; negative regulation of mesodermal cell fate specification; regulation of endodermal cell fate specification; positive regulation of JUN kinase activity; positive regulation of Wnt signaling pathway, calcium modulating pathway; synapse pruning; negative regulation of canonical Wnt signaling pathway involved in cardiac muscle cell fate commitment; regulation of dopaminergic neuron differentiation; negative regulation of Wnt-Frizzled-LRP5/6 complex assembly; positive regulation of Wnt signaling pathway, planar cell polarity pathway	-2,21	5,4E-02	-3,08	9,7E-03
99	122	147	**63**	**17**	**57**	244	161	226	**OGFRL1**	opioid growth factor receptor like 1	6	opioid receptor signaling pathway	-1,87	6,0E-02	-0,14	1,0E+00

2109	2353	2101	**2755**	**900**	**1988**	3012	2409	2757	**TLN2**	talin 2	15	cell adhesion; obsolete cytoskeletal anchoring at plasma membrane; cytoskeleton organization; cell-cell adhesion; cell-cell junction assembly; endocytosis	-1,58	6,1E-02	-0,46	1,0E+00
706	957	933	**1012**	**219**	**357**	2332	2570	2178	**ADCYAP1R1**	ADCYAP receptor type I	7	G protein-coupled receptor signaling pathway; cell surface receptor signaling pathway; multicellular organism development; cell differentiation; signal transduction; spermatogenesis; adenylate cyclase-modulating G protein-coupled receptor signaling pathway; activation of phospholipase C activity; positive regulation of calcium ion transport into cytosol; cAMP-mediated signaling; response to estradiol; multicellular organismal response to stress; response to drug; positive regulation of cAMP-mediated signaling; response to ethanol; development of primary female sexual characteristics; positive regulation of small GTPase mediated signal transduction; negative regulation of cell death; positive regulation of inositol phosphate biosynthetic process	-2,15	6,7E-02	-1,43	9,1E-01
67	79	93	**441**	**87**	**218**	269	172	297	**HPCA**	hippocalcin	1	regulation of voltage-gated calcium channel activity; cellular response to calcium ion; brain development; positive regulation of phospholipase activity; response to organic cyclic compound; calcium-mediated signaling; negative regulation of guanylate cyclase activity; activation of phospholipase D activity; positive regulation of adenylate cyclase activity; inner ear development; retina development in camera-type eye; cellular response to electrical stimulus; positive regulation of protein targeting to membrane; regulation of postsynaptic neurotransmitter receptor internalization; response to ketamine; response to L-glutamate; cellular response to monosodium glutamate; response to Aroclor 1254	-2,21	7,9E-02	-0,94	1,0E+00
84	121	102	**5291**	**1142**	**1677**	3101	1858	2804	**WSCD2**	WSC domain containing 2	12		-2,07	7,9E-02	-1,54	8,0E-01
1427	505	675	**1093**	**273**	**491**	2460	2989	2585	**PRSS12**	serine protease 12	4	proteolysis; exocytosis; endocytosis; zymogen activation	-1,95	8,4E-02	-1,11	1,0E+00
209	162	169	**438**	**180**	**221**	760	695	625	**SH3GL2**	SH3 domain containing GRB2 like 2, endophilin A1	9	negative regulation of gene expression; negative regulation of protein phosphorylation; endocytosis; antigen processing and presentation of exogenous peptide antigen via MHC class II; signal transduction; central nervous system development; membrane organization; neuron projection development; negative regulation of epidermal growth factor receptor signaling pathway; negative regulation of blood-brain barrier permeability; dendrite extension; cellular response to brain-derived neurotrophic factor stimulus; regulation of receptor internalization; synaptic vesicle uncoating; synaptic vesicle endocytosis; lipid tube assembly; membrane tubulation; membrane bending; vesicle scission; positive regulation of membrane tubulation; regulation of clathrin-dependent endocytosis	-1,26	8,9E-02	-0,97	8,0E-01
313	258	282	**2409**	**827**	**1240**	3613	4047	3832	**DOK5**	docking protein 5	20	axon guidance; transmembrane receptor protein tyrosine kinase signaling pathway; neuron differentiation; positive regulation of MAPK cascade; regulation of neurotrophin TRK receptor signaling pathway	-1,50	9,9E-02	-0,92	1,0E+00
4643	5228	4547	**812**	**331**	**504**	1390	1150	1184	**PCDH1**	protocadherin 1	5	homophilic cell adhesion via plasma membrane adhesion molecules; cell adhesion; nervous system development; cell-cell signaling	-1,29	1,1E-01	-0,68	1,0E+00
277	92	221	**108**	**31**	**90**	160	46	89	**FXN**	frataxin	9	iron-sulfur cluster assembly; oxidation-reduction process; positive regulation of cell population proliferation; negative regulation of apoptotic process; ion transport; cellular iron ion homeostasis; oxidative phosphorylation; heme biosynthetic process; mitochondrion organization; adult walking behavior; aerobic respiration; embryo development ending in birth or egg hatching; proprioception; negative regulation of multicellular organism growth; negative regulation of organ growth; iron ion homeostasis; positive regulation of cell growth; cellular response to hydrogen peroxide; negative regulation of release of cytochrome c from mitochondria; protein autoprocessing; positive regulation of catalytic activity; small molecule metabolic process; response to iron ion; regulation of ferrochelatase activity; iron incorporation into metallo-sulfur cluster; positive regulation of lyase activity; positive regulation of succinate dehydrogenase activity; positive regulation of aconitate hydratase activity	-1,77	1,2E-01	-0,26	1,0E+00
33154	40	35	**21340**	**34**	**27**	14136	20	41	**AP000763.2**	mitochondrially encoded cytochrome c oxidase I (MT-COI) pseudogene	11		-9,15	6,0E-113	-9,41	9,7E-75
552	3	2	**421**	**2**	**1**	305	1	2	**AC098847.1**	ribosomal protein, large, P0 pseudogene	18		-7,25	2,4E-34	-7,68	8,2E-21
153	1	0	**102**	**2**	**1**	86	1	0	**ZBTB8OSP2**	zinc finger and BTB domain containing 8 opposite strand pseudogene 2	2		-5,13	4,2E-08	-6,16	6,0E-06
48	9	7	**56**	**7**	**4**	55	7	2	**PSMA6P1**	proteasome subunit alpha 6 pseudogene 1	Y		-2,97	3,1E-07	-3,54	6,0E-06
1051	53	127	**61**	**1**	**1**	40	1	1	**RHOXF1-AS1**	RHOXF1 antisense RNA 1	X		-5,34	7,2E-07	-4,71	2,8E-04
30	1	2	**81**	**3**	**2**	77	2	3	**LERFS**	lncRNA	9		-4,51	8,9E-07	-4,93	1,4E-05
50	7	14	**111**	**7**	**8**	186	16	29	**BX284668.2**	novel transcript	1		-3,80	3,9E-06	-3,57	4,1E-04
60	17	20	**404**	**104**	**98**	364	115	126	**AF274858.1**	Morf4 family associated protein 1 (MRFAP1) pseudogene	X		-1,93	4,8E-06	-2,00	4,7E-05
442	84	51	**223**	**36**	**26**	185	31	16	**SORD2P**	sorbitol dehydrogenase 2, pseudogene	15		-2,55	5,3E-04	-2,99	1,9E-04
117	18	20	**86**	**14**	**16**	101	10	12	**AC087343.1**	ribosomal protein L21 (RPl21) pseudogene	8		-2,54	7,4E-04	-2,34	2,8E-02
46	7	9	**114**	**32**	**22**	109	52	56	**RASA4CP**	RAS p21 protein activator 4C, pseudogene	7		-1,79	3,0E-03	-2,32	2,3E-04
128	13	14	**146**	**8**	**3**	115	6	7	**AACSP1**	acetoacetyl-CoA synthetase pseudogene 1	5		-3,84	6,2E-03	-5,11	2,8E-04
0	0	0	**82**	**28**	**41**	52	76	64	**AC016717.2**	novel transcript	2		-1,49	2,0E-02	-0,96	8,0E-01

92	34	40	**89**	**32**	**39**	107	31	51	**LYRM4-AS1**	LYRM4 antisense RNA 1	6		-1,46	2,1E-02	-1,17	4,7E-01
3	4	4	**1036**	**310**	**267**	315	482	255	**LINC01933**	long intergenic non-protein coding RNA 1933	5		-1,64	7,2E-02	-1,87	1,1E-01

MATURE NEURONS

UPREGULATED GENES

Normalized read counts									GENE	Description	Chr	GO: Biological process	Log$_2$FC FRDA/CT	FDR	Log$_2$FC ISO CT/CT	FDR
iPSCs			Developing Neurons			Mature Neurons										
CT	FRDA	ISO CT	CT	FRDA	ISO CT	CT	FRDA	ISO CT								
0	0	0	2	31	38	**17**	**735**	**646**	**GRIN3A**	glutamate ionotropic receptor NMDA type subunit 3A	9	ion transport; ion transmembrane transport; calcium ion transport; calcium ion transmembrane transport; regulation of postsynaptic membrane potential; ionotropic glutamate receptor signaling pathway; response to ethanol; dendrite development; prepulse inhibition; regulation of synaptic vesicle exocytosis	5,36	3,1E-12	5,08	5,7E-09
122	192	190	234	533	531	**194**	**500**	**441**	**MINDY1**	MINDY lysine 48 deubiquitinase 1	1	proteolysis; protein K48-linked deubiquitination	1,36	1,1E-08	1,18	5,5E-05
10	144	122	15	213	161	**4**	**124**	**88**	**PCDHA11**	protocadherin alpha 11	5	homophilic cell adhesion via plasma membrane adhesion molecules; cell adhesion; nervous system development	4,52	6,9E-06	4,02	1,7E-03
2	6	1	6	35	4	**1**	**27**	**2**	**PCDHA9**	protocadherin alpha 9	5	homophilic cell adhesion via plasma membrane adhesion molecules; cell adhesion	4,46	7,7E-06	0,82	1,0E+00
51	79	89	11	30	22	**4**	**57**	**27**	**RORB**	RAR related orphan receptor B	9	regulation of transcription, DNA-templated; multicellular organism development; intracellular receptor signaling pathway; rhythmic process; visual perception; positive regulation of transcription, DNA-templated; response to stimulus; G protein-coupled receptor signaling pathway; positive regulation of transcription by RNA polymerase II; cellular response to retinoic acid; negative regulation of transcription, DNA-templated; transcription initiation from RNA polymerase II promoter; eye photoreceptor cell development; retina development in camera-type eye; regulation of circadian rhythm; negative regulation of osteoblast differentiation; retinal cone cell development; retinal rod cell development; amacrine cell differentiation	3,50	9,6E-05	2,47	1,5E-01
31	92	85	30	133	157	**12**	**91**	**94**	**PCDHGA10**	protocadherin gamma subfamily A, 10	5	homophilic cell adhesion via plasma membrane adhesion molecules; cell adhesion	2,83	9,6E-05	2,88	7,8E-04
22	40	49	19	69	61	**18**	**48**	**44**	**TSTD3**	thiosulfate sulfurtransferase like domain containing 3	6		1,39	5,9E-04	1,27	2,2E-02
1	399	429	1	1224	1163	**1**	**739**	**1068**	**TAFA5**	TAFA chemokine like family member 5	22	signal transduction	5,68	9,3E-04	6,01	1,8E-03
127	118	141	768	1061	1160	**516**	**1117**	**966**	**C20orf194**	chromosome 20 open reading frame 194	20		1,11	2,3E-03	0,90	1,7E-01
14	5	6	960	1634	1042	**161**	**899**	**679**	**NDUFA4L2**	NDUFA4 mitochondrial complex associated like 2	12	proton transmembrane transport; electron transport chain	2,44	3,4E-03	2,01	1,7E-01
20	62	68	41	130	118	**32**	**99**	**109**	**WASHC1**	WASH complex subunit 1	9	Arp2/3 complex-mediated actin nucleation; protein transport; exocytosis; extracellular matrix disassembly; positive regulation of cell migration; negative regulation of autophagy; endosomal transport; positive regulation of pseudopodium assembly; regulation of protein ubiquitination; retrograde transport, endosome to Golgi; negative regulation of phosphatidylinositol 3-kinase activity; retrograde transport, endosome to plasma membrane	1,62	3,5E-03	1,75	6,3E-03
22	42	65	575	426	468	**192**	**615**	**452**	**PRKG2**	protein kinase cGMP-dependent 2	4	protein phosphorylation; phosphorylation; signal transduction; peptidyl-serine autophosphorylation; protein localization to plasma membrane; negative regulation of chloride transport	1,67	7,3E-03	1,22	5,3E-01
178	179	178	1167	1158	935	**383**	**1016**	**716**	**SLC10A7**	solute carrier family 10 member 7	4	ion transport; sodium ion transport; transmembrane transport; bone development; heparin biosynthetic process; Golgi vesicle transport; cellular calcium ion homeostasis; bile acid and bile salt transport; glycoprotein transport	1,40	7,3E-03	0,89	8,5E-01
1	1	0	11	16	17	**4**	**54**	**43**	**PLA2G12B**	phospholipase A2 group XIIB	10	phospholipid metabolic process; lipid catabolic process; arachidonic acid secretion; cholesterol homeostasis; triglyceride homeostasis	3,68	8,0E-03	3,29	1,3E-01
10886	15191	13263	8254	19031	21163	**6392**	**12889**	**16069**	**RPS23**	ribosomal protein S23	5	translation; cytoplasmic translation; viral transcription; translational initiation; stress granule assembly; nuclear-transcribed mRNA catabolic process, nonsense-mediated decay; SRP-dependent cotranslational protein targeting to membrane; maintenance of translational fidelity	1,00	1,7E-02	1,32	1,1E-03
6	14	17	5	59	58	**2**	**22**	**20**	**PCDHB8**	protocadherin beta 8	5	homophilic cell adhesion via plasma membrane adhesion molecules; cell adhesion	3,00	2,6E-02	2,90	1,3E-01
14	23	38	54	1074	203	**2**	**45**	**22**	**OTX1**	orthodenticle homeobox 1	2	regulation of transcription, DNA-templated; multicellular organism development; positive regulation of transcription by RNA polymerase II; regulation of transcription by RNA polymerase II; anterior/posterior pattern specification; metencephalon development; forebrain development; midbrain development; inner ear morphogenesis; diencephalon morphogenesis	3,71	4,3E-02	2,81	7,6E-01
288	267	294	2101	2305	1412	**737**	**1701**	**1095**	**SRRM3**	serine/arginine repetitive matrix 3	7		1,20	4,4E-02	0,56	1,0E+00
176	157	185	1218	2469	1422	**189**	**440**	**308**	**MUC1**	mucin 1, cell surface associated	1	stimulatory C-type lectin receptor signaling pathway; cytokine-mediated signaling pathway; negative regulation of transcription by competitive promoter binding; O-glycan processing; negative regulation of intrinsic apoptotic signaling pathway in response to DNA damage by p53 class mediator; DNA damage response, signal transduction by p53 class mediator resulting in transcription of p21 class	1,20	6,2E-02	0,69	1,0E+00

												mediator; DNA damage response, signal transduction by p53 class mediator resulting in cell cycle arrest; negative regulation of cell adhesion mediated by integrin; positive regulation of transcription from RNA polymerase II promoter in response to stress; regulation of transcription from RNA polymerase II promoter in response to stress; positive regulation of histone H4 acetylation				
24	20	23	111	232	621	**60**	**607**	**627**	**ITGA11**	integrin subunit alpha 11	15	cell adhesion; integrin-mediated signaling pathway; extracellular matrix organization; cell-matrix adhesion; muscle organ development; osteoblast differentiation; cell adhesion mediated by integrin; substrate-dependent cell migration; collagen-activated signaling pathway	3,10	6,4E-02	3,08	2,3E-01
43	54	47	2235	1598	1850	**938**	**2485**	**1836**	**MTUS2**	microtubule associated scaffold protein 2	13		1,40	6,4E-02	0,96	1,0E+00
2767	3382	2412	3207	3835	3013	**2531**	**6633**	**4239**	**MVD**	mevalonate diphosphate decarboxylase	16	isoprenoid biosynthetic process; isopentenyl diphosphate biosynthetic process, mevalonate pathway; lipid metabolic process; steroid biosynthetic process; cholesterol biosynthetic process; steroid metabolic process; cholesterol metabolic process; sterol biosynthetic process; positive regulation of cell population proliferation; regulation of cholesterol biosynthetic process; dolichyl diphosphate biosynthetic process	1,37	6,4E-02	0,73	1,0E+00
543	399	472	151	442	264	**119**	**384**	**375**	**TRAF5**	TNF receptor associated factor 5	1	signal transduction; regulation of apoptotic process; positive regulation of I-kappaB kinase/NF-kappaB signaling; positive regulation of DNA-binding transcription factor activity; apoptotic process; regulation of I-kappaB kinase/NF-kappaB signaling; positive regulation of NF-kappaB transcription factor activity; protein K63-linked ubiquitination; tumor necrosis factor-mediated signaling pathway; positive regulation of JNK cascade	1,64	8,3E-02	1,60	3,3E-01
371	477	336	4	396	191	**17**	**341**	**188**	**COMT**	catechol-O-methyltransferase	22	catecholamine metabolic process; neurotransmitter catabolic process; methylation; developmental process; dopamine metabolic process; catecholamine catabolic process; response to organic cyclic compound; response to drug; short-term memory; cellular response to phosphate starvation; dopamine catabolic process; female pregnancy; learning; estrogen metabolic process; response to lipopolysaccharide; negative regulation of renal sodium excretion; response to estrogen; negative regulation of dopamine metabolic process; response to pain; multicellular organismal reproductive process; negative regulation of smooth muscle cell proliferation; positive regulation of homocysteine metabolic process; regulation of sensory perception of pain	3,48	1,1E-01	2,68	9,4E-01
20	2624	2895	20	37489	38283	**25**	**15558**	**15743**	**NNAT**	neuronatin	20	brain development; multicellular organism development; positive regulation of insulin secretion; protein lipoylation	4,30	1,5E-01	4,17	4,5E-01
2	427	451	4	538	707	**5**	**286**	**468**	**RPS4XP22**	ribosomal protein S4X pseudogene 22	19		5,71	2,3E-23	6,40	5,4E-28
11	7	9	9502	15271	10959	**465**	**1969**	**2083**	**AL161908.1**	novel transcript, antisense to LIM1B	9		2,06	1,1E-03	2,12	5,7E-03
4	1	1	9	10	23	**2**	**131**	**47**	**MIR4527HG**	MIR4527 host gene	18		4,95	1,5E-02	3,47	6,9E-01
39	73	73	52	69	69	**49**	**118**	**98**	**AC026304.1**	novel transcript	3		1,27	2,0E-02	0,99	5,8E-01
43	166	124	210	1927	1392	**34**	**441**	**160**	**H19**	H19 imprinted maternally expressed transcript	11		3,28	3,2E-02	1,91	1,0E+00
0	0	1	43	65	51	**7**	**50**	**49**	**AL139351.3**	novel transcript	20		2,75	4,6E-02	2,67	2,1E-01
1	0	0	410	381	413	**236**	**639**	**514**	**AC091980.2**	novel transcript, antisense to TLX3	5		1,45	7,5E-02	1,12	8,7E-01
72	31	54	15	25	30	**5**	**58**	**31**	**AC087276.1**	novel transcript, sense overlapping TTC17	11		3,04	9,4E-02	2,17	1,0E+00

DOWNREGULATED GENES

Normalized read counts									GENE	Description	Chr	GO: Biological process	Log2FC FRDA/CT	FDR	Log2FC ISO CT/CT	FDR
iPSCs			Developing Neurons			Mature Neurons										
CT	FRDA	ISO CT	CT	FRDA	ISO CT	CT	FRDA	ISO CT								
19	0	0	18	0	0	**11**	**0**	**0**	**EIF3CL**	eukaryotic translation initiation factor 3 subunit C like	16	translational initiation; formation of cytoplasmic translation initiation complex; cytoplasmic translational initiation; translation	-5,82	2,4E-08	-4,62	4,2E-05
8433	8351	7939	75	28	39	**156**	**30**	**47**	**EPCAM**	epithelial cell adhesion molecule	2	ureteric bud development; negative regulation of apoptotic process; cell-cell adhesion via plasma-membrane adhesion molecules; positive regulation of cell motility; leukocyte migration; positive regulation of transcription by RNA polymerase II; stem cell differentiation; positive regulation of cell population proliferation; positive regulation of stem cell proliferation; signal transduction involved in regulation of gene expression; negative regulation of cell-cell adhesion mediated by cadherin	-2,35	4,3E-07	-1,72	1,5E-02
243	285	225	598	568	810	**5040**	**773**	**1177**	**CBLN1**	cerebellin 1 precursor	16	nervous system development; chemical synaptic transmission; synapse organization; heterophilic cell-cell adhesion via plasma membrane cell adhesion molecules; cerebellar granule cell differentiation; positive regulation of long-term synaptic depression; protein secretion; positive regulation of synapse assembly; negative regulation of excitatory postsynaptic potential; regulation of postsynaptic density assembly; maintenance of synapse structure; regulation of presynapse assembly; negative regulation of inhibitory synapse assembly	-2,62	3,2E-06	-2,03	1,6E-02
88	64	65	348	56	65	**255**	**84**	**102**	**SAXO2**	stabilizer of axonemal microtubules 2	15	microtubule anchoring	-1,58	2,1E-04	-1,31	5,0E-02
210	100	85	196	90	120	**236**	**103**	**128**	**THNSL1**	threonine synthase like 1	10		-1,18	3,0E-04	-0,87	1,7E-01

3	5	8	0	0	0	**46**	**1**	**4**	**UTS2**	urotensin 2	1	regulation of blood vessel diameter; regulation of blood pressure; G protein-coupled receptor signaling pathway; muscle contraction; chemical synaptic transmission	-4,38	7,3E-03	-3,17	4,2E-01
3	4	6	314	90	97	**1055**	**130**	**453**	**SV2C**	synaptic vesicle glycoprotein 2C	5	transmembrane transport; chemical synaptic transmission; neurotransmitter transport	-2,79	8,7E-03	-1,09	1,0E+00
19	6	13	0	0	0	**4**	**0**	**1**	**RUNX3**	RUNX family transcription factor 3	1	regulation of transcription, DNA-templated; positive regulation of transcription, DNA-templated; regulation of transcription by RNA polymerase II; neuron differentiation; regulation of cell differentiation; negative regulation of transcription by RNA polymerase II; negative regulation of cell cycle; protein phosphorylation; hemopoiesis; negative regulation of epithelial cell proliferation; ossification; negative regulation of CD4-positive, alpha-beta T cell differentiation; chondrocyte differentiation; peripheral nervous system neuron development; positive regulation of CD8-positive, alpha-beta T cell differentiation; response to transforming growth factor beta	-3,49	1,5E-02	-1,81	1,0E+00
45	65	84	4	2	3	**297**	**22**	**99**	**PTGER4**	prostaglandin E receptor 4	5	G protein-coupled receptor signaling pathway; signal transduction; immune response; bone development; positive regulation of inflammatory response; negative regulation of inflammatory response; cellular response to mechanical stimulus; inflammatory response; ERK1 and ERK2 cascade; positive regulation of cytosolic calcium ion concentration; adenylate cyclase-activating G protein-coupled receptor signaling pathway; JNK cascade; negative regulation of cytokine secretion; regulation of stress fiber assembly; positive regulation of cytokine secretion; adenylate cyclase-modulating G protein-coupled receptor signaling pathway; T-helper cell differentiation; response to mechanical stimulus; regulation of ossification; response to lipopolysaccharide; negative regulation of integrin activation; cellular response to prostaglandin E stimulus; negative regulation of eosinophil extravasation	-3,34	3,3E-02	-1,37	1,0E+00
3	3	8	6	4	6	**47**	**8**	**6**	**METTL24**	methyltransferase like 24	6	methylation	-2,42	4,3E-02	-2,90	4,6E-02
1971	1862	1855	893	1052	955	**1355**	**529**	**603**	**ETV6**	ETS variant transcription factor 6	12	regulation of transcription, DNA-templated; regulation of transcription by RNA polymerase II; vitellogenesis; neurogenesis; cell differentiation; positive regulation of transcription by RNA polymerase II; mesenchymal cell apoptotic process; negative regulation of transcription by RNA polymerase II; hematopoietic stem cell proliferation	-1,34	4,3E-02	-1,15	5,0E-01
6180	5493	5431	166	146	162	**498**	**219**	**221**	**HAS3**	hyaluronan synthase 3	16	positive regulation of transcription, DNA-templated; carbohydrate metabolic process; hyaluronan biosynthetic process; extracellular polysaccharide biosynthetic process; extracellular matrix assembly; positive regulation of hyaluranon cable assembly	-1,18	6,4E-02	-1,16	2,7E-01
294	332	304	13	23	24	**187**	**51**	**102**	**PPP1R16B**	protein phosphatase 1 regulatory subunit 16B	20	regulation of phosphoprotein phosphatase activity; regulation of phosphatidylinositol 3-kinase signaling; negative regulation of protein dephosphorylation; positive regulation of endothelial cell proliferation; regulation of filopodium assembly; positive regulation of protein dephosphorylation; regulation of protein dephosphorylation; establishment of endothelial barrier; negative regulation of peptidyl-serine dephosphorylation; positive regulation of blood vessel endothelial cell proliferation involved in sprouting angiogenesis; regulation of sprouting angiogenesis	-1,82	6,4E-02	-0,84	1,0E+00
38	61	79	2	1	1	**47**	**4**	**11**	**PVALB**	parvalbumin	22	cochlea development	-3,05	7,8E-02	-1,88	1,0E+00
71	80	58	78	49	40	**71**	**17**	**37**	**DACT2**	dishevelled binding antagonist of beta catenin 2	6	negative regulation of cell adhesion; epithelial cell morphogenesis; skin development; inner medullary collecting duct development; negative regulation of nodal signaling pathway; hematopoietic progenitor cell differentiation	-1,99	8,4E-02	-0,92	1,0E+00
553	901	925	70	50	53	**284**	**97**	**116**	**SOCS3**	suppressor of cytokine signaling 3	17	intracellular signal transduction; negative regulation of apoptotic process; receptor signaling pathway via JAK-STAT; protein ubiquitination; negative regulation of signal transduction; regulation of growth; regulation of phosphatidylinositol 3-kinase activity; phosphatidylinositol phosphorylation; negative regulation of protein kinase activity; post-translational protein modification; cytokine-mediated signaling pathway; regulation of protein phosphorylation; positive regulation of tyrosine phosphorylation of STAT protein; negative regulation of tyrosine phosphorylation of STAT protein; regulation of cell differentiation; positive regulation of cell differentiation; negative regulation of receptor signaling pathway via JAK-STAT; negative regulation of insulin receptor signaling pathway; negative regulation of inflammatory response; regulation of interferon-gamma-mediated signaling pathway; branching involved in labyrinthine layer morphogenesis; placenta blood vessel development; trophoblast giant cell differentiation; spongiotrophoblast differentiation; interleukin-6-mediated signaling pathway; cellular response to leukemia inhibitory factor	-1,52	8,7E-02	-1,27	7,4E-01
447	526	642	47	25	40	**157**	**58**	**83**	**DOCK9**	dedicator of cytokinesis 9	13	small GTPase mediated signal transduction; positive regulation of GTPase activity; blood coagulation	-1,40	1,4E-01	-0,89	1,0E+00
376	434	335	5	3	6	**51**	**14**	**14**	**SYNJ2**	synaptojanin 2	6	phosphatidylinositol dephosphorylation; membrane organization; phosphatidylinositol biosynthetic process; synaptic vesicle endocytosis; brain development; inositol phosphate dephosphorylation	-1,82	1,5E-01	-1,80	4,9E-01
277	92	221	108	31	90	**160**	**46**	**89**	**FXN**	frataxin	9	iron-sulfur cluster assembly; oxidation-reduction process; positive regulation of cell population proliferation; negative regulation of apoptotic process; ion transport; cellular iron ion homeostasis; oxidative phosphorylation; heme biosynthetic process; mitochondrion organization; adult walking behavior; aerobic respiration; embryo development ending in birth or egg hatching; proprioception; negative regulation of multicellular organism growth; negative regulation of organ growth; iron ion homeostasis; positive regulation of cell growth; cellular response to hydrogen peroxide; negative regulation of release of cytochrome c from mitochondria; protein autoprocessing; positive regulation of catalytic activity; small molecule metabolic process; response to iron ion; regulation of ferrochelatase activity; iron incorporation into metallo-sulfur cluster; positive regulation of lyase activity; positive regulation of succinate dehydrogenase activity; positive regulation of aconitate hydratase activity	-1,75	2,1E-01	-0,81	1,0E+00
33154	40	35	21340	34	27	**14136**	**20**	**41**	**AP000763.2**	mitochondrially encoded cytochrome c oxidase I (MT-COI) pseudogene	11		-9,36	3,0E-95	-8,27	3,5E-72
552	3	2	421	2	1	**305**	**1**	**2**	**AC098847.1**	ribosomal protein, large, P0 pseudogene	18		-7,42	3,9E-29	-7,02	1,2E-19
44	14	15	32	11	8	**80**	**14**	**16**	**AC012184.3**	novel transcript, antisense to DDX19B and DDX19A	16		-2,53	6,5E-10	-2,29	4,7E-06
153	1	0	102	2	1	**86**	**1**	**0**	**ZBTB8OSP2**	zinc finger and BTB domain containing 8 opposite strand pseudogene 2	2		-5,85	2,4E-08	-6,73	7,6E-07
4	0	0	59	0	0	**55**	**0**	**0**	**ADGRF5P1**	adhesion G protein-coupled receptor F5 pseudogene 1	9		-7,27	1,8E-07	-6,87	4,2E-05

12	5	2	87	28	22	**109**	**13**	**15**	**AC027575.2**	novel transcript	18		-2,97	2,6E-07	-2,77	1,4E-04
117	18	20	86	14	16	**101**	**10**	**12**	**AC087343.1**	ribosomal protein L21 (RPl21) pseudogene	8		-3,17	2,3E-06	-2,96	7,5E-04
1051	53	127	61	1	1	**40**	**1**	**1**	**RHOXF1-AS1**	RHOXF1 antisense RNA 1	X		-4,69	2,6E-05	-4,24	3,9E-03
50	7	14	111	7	8	**186**	**16**	**29**	**BX284668.2**	novel transcript	1		-3,37	4,5E-05	-2,51	6,8E-02
60	17	20	404	104	98	**364**	**115**	**126**	**AF274858.1**	Morf4 family associated protein 1 (MRFAP1) pseudogene	X		-1,63	4,0E-04	-1,50	1,7E-02
442	84	51	223	36	26	**185**	**31**	**16**	**SORD2P**	sorbitol dehydrogenase 2, pseudogene	15		-2,53	6,7E-04	-3,43	8,3E-06
92	34	40	89	32	39	**107**	**31**	**51**	**LYRM4-AS1**	LYRM4 antisense RNA 1	6		-1,76	1,2E-03	-1,05	7,8E-01
12	6	5	41	21	22	**43**	**14**	**18**	**AC009318.1**	novel transcript, antisense to FAR2	12		-1,57	4,4E-03	-1,24	2,8E-01
128	13	14	146	8	3	**115**	**6**	**7**	**AACSP1**	acetoacetyl-CoA synthetase pseudogene 1	5		-3,89	7,0E-03	-3,55	9,5E-02
219	30	44	102	9	1	**140**	**6**	**4**	**LINC00668**	long intergenic non-protein coding RNA 668	18		-4,03	4,0E-02	-4,28	8,6E-02
1	0	1	50	11	22	**74**	**8**	**27**	**AC022182.2**	novel transcript	8		-2,84	7,5E-02	-1,27	1,0E+00
27	4	5	50	2	3	**96**	**14**	**18**	**LINC01783**	long intergenic non-protein coding RNA 1783	1		-2,58	9,7E-02	-2,17	7,3E-01

Supplementary Table 3. Gene and protein expression of commonly identified markers in FRDA.

Gene name	Description	Normalized read counts			Log$_2$ FC (FRDA vs CT)	Adj p-value	Mean peak intensity			Log$_2$ FC (FRDA vs CT)	p-value
		Mean CT	Mean FRDA	Mean ISO CT			Mean CT	Mean FRDA	Mean ISO CT		
**IRON HOMEOSTASIS**											
**ACO1 (IRP1)**	aconitase 1	376,17	396,17	377,87	**0,071**	0,968	14441,8	12354,5	21312,1	**-0,225**	0,424
**IREB2 (IRP2)**	iron responsive element binding protein 2	9898,82	10504,92	9772,08	**0,085**	0,946					
**FTH1**	ferritin heavy chain 1	6297,04	8423,56	8313,49	**0,417**	0,718	13712,5	12471,7	7112,4	**-0,137**	0,654
**FTL**	ferritin light chain	13635,7	16389,20	16557,82	**0,261**	0,849	10441,2	14490,8	17710,8	**0,473**	0,231
**IREG1**	ferroportin-1	130,72	116,48	172,49	**-0,019**	0,999					
**TFRC**	transferrin receptor	4778,88	4439,17	5277,34	**-0,116**	0,962	14091,0	16383,5	22994,0	**0,217**	0,429
**FBXL5**	F-box and leucine rich repeat protein 5	4546,78	4483,85	4778,91	**-0,020**	0,989					
**TF**	transferrin	56,52	106,76	133,34	**0,905**	0,720	66054,1	53408,9	41030,9	**-0,307**	0,171
**TFRC**	transferrin receptor	4778,88	4439,17	5277,34	**-0,116**	0,962					
**TFR2**	transferrin receptor 2	85,24	136,71	133,94	**0,674**	0,716					
**SLC25A28**	mitoferrin-2	695,91	771,53	693,24	**0,149**	0,922					
**DMT1**	solute carrier family 11 member 2	2843,03	2777,61	2615,55	**-0,035**	0,980					
**HIF1A**	hypoxia inducible factor 1 subunit alpha	8431,72	8814,44	8051,17	**0,062**	0,968					
**HIF1AN**	hypoxia inducible factor 1 subunit alpha inhibitor	4709,07	4254,29	4498,54	**-0,146**	0,790					
**EPAS1 (HIF2A)**	endothelial PAS domain protein 1	42,40	25,51	17,35	**-0,696**	0,869					
**Fe-S CLUSTER METABOLISM**											
**NFS1**	NFS1 cysteine desulfurase	519,05	445,19	488,85	**-0,221**	0,782	4592,0	3321,8	3360,0	**-0,467**	0,148
**ISCU**	iron-sulfur cluster assembly enzyme	1542,55	1898,36	1801,57	**0,298**	0,570					
**LYRM4 (ISD11)**	LYR motif containing 4	1063,58	1088,79	1135,61	**0,033**	0,984					
**ISCA1**	iron-sulfur cluster assembly 1	1556,89	1490,26	1549,56	**-0,063**	0,980	5923,7	7379,9	5711,2	**0,317**	0,607
**ISCA2**	iron-sulfur cluster assembly 2	516,06	440,48	502,23	**-0,228**	0,835					
**FDX1**	ferredoxin 1	114,96	102,59	95,46	**-0,166**	0,913					
**FDXR**	ferredoxin reductase	169,32	181,38	148,10	**0,093**	0,974	18131,2	15805,7	17247,4	**-0,198**	0,635
**HSPA9**	heat shock protein family A (Hsp70) member 9	2157,18	1818,66	1837,56	**-0,247**	0,809	667919,4	770581,7	788489	**0,206**	0,226
**HSCB**	HscB mitochondrial iron-sulfur cluster cochaperone	190,57	180,39	183,92	**-0,083**	0,962					
**GLRX**	glutaredoxin	806,28	1035,14	847,88	**0,356**	0,819	18840,9	16460,3	17493,4	**-0,195**	0,578
**GLRX2**	glutaredoxin 2	332,32	336,17	327,15	**0,016**	0,994					
**GLRX3**	glutaredoxin 3	2524,20	2666,78	2548,81	**0,078**	0,962	105363,3	121356,2	131291	**0,204**	0,135
**NUBP1**	nucleotide binding protein 1	855,26	712,76	649,25	**-0,262**	0,825					
**NUBP2**	nucleotide binding protein 2	656,82	932,51	747,65	**0,495**	0,750	3785,5	4910,2	8705,8	**0,375**	0,384
**NARF**	nuclear prelamin A recognition factor	5655,53	5591,14	5798,12	**-0,014**	0,994					
**CIAO1**	cytosolic iron-sulfur assembly component 1	2094,92	2110,34	2053,77	**0,011**	0,991	1429,7	4026	3406,1	**1,494**	0,011
**MITOCHONDRIAL Fe-S PROTEINS**											
**NDUFS1**	NADH:ubiquinone oxidoreductase core subunit S1	2626,51	3040,60	2826,87	**0,211**	0,795	112035,3	133029,9	116340	**0,248**	0,088
**NDUFS2**	NADH:ubiquinone oxidoreductase core subunit S2	1993,35	1919,07	2013,20	**-0,054**	0,963	121309,6	120737,1	112029	**-0,007**	0,958
**NDUFS7**	NADH:ubiquinone oxidoreductase core subunit S7	479,92	849,93	586,45	**0,802**	0,669	34396,8	46721,1	44989,1	**0,442**	0,021
**NDUFS8**	NADH:ubiquinone oxidoreductase core subunit S8	1213,85	1702,99	1390,34	**0,479**	0,745	50235,0	78825,7	63470,6	**0,650**	0,136
**NDUFV1**	NADH:ubiquinone oxidoreductase core subunit V1	4511,76	5241,70	4802,20	**0,215**	0,790	71843,9	64869,2	50453,0	**-0,147**	0,141
**NDUFV2**	NADH:ubiquinone oxidoreductase core subunit V2	34,28	60,87	39,30	**0,794**	0,791	107372,6	139448,4	136270	**0,377**	0,153
**SDHB**	succinate dehydrogenase complex iron sulfur subunit B	1270,98	1061,55	1073,89	**-0,260**	0,779	48769,6	45546,4	46100,7	**-0,099**	0,645
**UQCRFS1**	Rieske iron-sulfur protein	492,89	382,46	450,67	**-0,363**	0,851	135660,2	121098,9	123410	**-0,164**	0,306
**ACO2**	aconitase 2	1333,28	1344,84	1372,49	**0,012**	0,993	212450,1	195053,9	174644	**-0,123**	0,213
**OXIDATIVE STRESS**											
**SOD2**	superoxide dismutase 2	8828,19	8381,57	8668,55	**-0,070**	0,974	132451,8	143580,6	104841	**0,116**	0,153
**CAT**	catalase	770,77	1152,69	945,46	**0,572**	0,772	54048,4	63429,7	30772,2	**0,231**	0,497
**NFE2L2**	nuclear factor, erythroid 2 like 2	1237,90	1194,35	1209,02	**-0,051**	0,982					
**DNA DAMAGE**											
**MSH2**	mutS homolog 2	1341,66	1213,53	1149,30	**-0,146**	0,919	36452,7	46858,0	65600,6	**0,362**	0,244
**MSH3**	mutS homolog 3	258,46	235,38	221,80	**-0,136**	0,918	1906,6	3248,7	2683,7	**0,769**	0,298
**MSH6**	mutS homolog 6	1900,62	1402,77	1608,32	**-0,435**	0,765	23783,5	40868,3	81032,6	**0,781**	0,091

Supplementary Table 4. List of identified differentially expressed proteins in FRDA and ISO CT mature neurons.

DIFFERENTIALLY EXPRESSED PROTEINS – FRDA vs CT										
UPREGULATED										
PROTEIN	GROUP AND RELATED GENE (GN)	GO: BIOLOGICAL FUNCTION	p-value	Protein Mean Peak Intensity					Log$_2$FC FRDA/CT
				Mean CT	Mean FRDA	Mean ISO CT	Median CT	Median FRDA	
sp|Q9H4L7-3|SMRCD_HUMAN	Isoform 3 of SWI/SNF-related matrix-associated actin-dependent regulator of chromatin subfamily A containing DEAD/H box 1 OS=Homo sapiens OX=9606 GN=SMARCAD1	ATPase activity, acting on DNA; ATP binding; chromatin binding; DNA binding; DNA helicase activity; nucleosome-dependent ATPase activity; ubiquitin bindin; ATP-dependent chromatin remodeling; chromatin remodeling; chromosome separation; DNA double-strand break processing; histone H3 deacetylation; histone H4 deacetylation; regulation of DNA recombination	**0,003**	**2383,1**	**6056,7**	4878,7	2501,5	6254,7	1,35
sp|Q9BRJ2|RM45_HUMAN	39S ribosomal protein L45, mitochondrial OS=Homo sapiens OX=9606 GN=MRPL45 PE=1 SV=2	RNA binding; mitochondrial translational elongation; mitochondrial translational termination	**0,006**	**3162,0**	**8172,4**	6993,8	2309,5	8422,2	1,37
sp|P19525|E2AK2_HUMAN	Interferon-induced, double-stranded RNA-activated protein kinase OS=Homo sapiens OX=9606 GN=EIF2AK2 PE=1 SV=2	ATP binding; double-stranded RNA binding; eukaryotic translation initiation factor 2alpha kinase activity; identical protein binding; non-membrane spanning protein tyrosine kinase activity; protein kinase activity; protein serine kinase activity; protein threonine kinase activity; transmembrane receptor protein tyrosine kinase activity; activation of MAPKK activity; cellular response to amino acid starvation; defense response to virus; endoplasmic reticulum unfolded protein response; innate immune response; negative regulation of apoptotic process; negative regulation of osteoblast proliferation; negative regulation of translation; negative regulation of viral genome replication; positive regulation of apoptotic process; positive regulation of chemokine production; positive regulation of cytokine production; positive regulation of NF-kappaB transcription factor activity; positive regulation of NIK/NF-kappaB signaling; positive regulation of stress-activated MAPK cascade; protein autophosphorylation; protein phosphorylation; regulation of hematopoietic progenitor cell differentiation; regulation of hematopoietic stem cell differentiation; regulation of hematopoietic stem cell proliferation; regulation of NLRP3 inflammasome complex assembly; response to interferon-alpha; response to lipopolysaccharide; response to toxic substance; response to virus; response to vitamin E; translation	**0,005**	**3072,5**	**11122,9**	11814,6	2972,5	8138,0	1,86
sp|A1L0T0|ILVBL_HUMAN	Acetolactate synthase-like protein OS=Homo sapiens OX=9606 GN=ILVBL PE=1 SV=2	acetolactate synthase activity; flavin adenine dinucleotide binding; lyase activity; magnesium ion binding; thiamine pyrophosphate bindingacetolactate synthase activity; flavin adenine dinucleotide binding; lyase activity; magnesium ion binding; thiamine pyrophosphate binding; fatty acid alpha-oxidation; isoleucine biosynthetic process; valine biosynthetic process	**0,003**	**14797,6**	**30474,7**	29355,4	14787,4	27224,7	1,04
sp|Q16822|PCKGM_HUMAN	Phosphoenolpyruvate carboxykinase [GTP], mitochondrial OS=Homo sapiens OX=9606 GN=PCK2 PE=1 SV=4	GTP binding; manganese ion binding; cellular response to dexamethasone stimulus; cellular response to glucose stimulus; cellular response to insulin stimulus; cellular response to tumor necrosis; gluconeogenesis; glycerol biosynthetic process from pyruvate; hepatocyte differentiation; NADH oxidation; oxaloacetate metabolic process; positive regulation of insulin secretion; propionate catabolic process; response to lipid; response to lipopolysaccharide; response to starvation	**0,008**	**15690,9**	**32336,3**	29279,3	17062,8	36017,7	1,04
sp|Q9UQ16-2|DYN3_HUMAN	Isoform 2 of Dynamin-3 OS=Homo sapiens OX=9606 GN=DNM3	GTPase activity; GTP binding; identical protein binding; microtubule binding; nitric-oxide synthase binding; structural constituent of postsynapse; type 1 metabotropic glutamate receptor binding; type 5 metabotropic glutamate receptor binding; dynamin family protein polymerization involved in mitochondrial fission; endocytosis; filopodium assembly; membrane fusion; mitochondrial fission; negative regulation of dendritic spine; morphogenesis; positive regulation of filopodium assembly; positive regulation of synaptic vesicle recycling; postsynaptic neurotransmitter receptor internalization; receptor internalization; regulation of synapse structure or activity; synapse assembly; synaptic vesicle budding from presynaptic endocytic zone membrane	**0,002**	**34483,6**	**73319,7**	39624,3	39350,4	76370,3	1,09
sp|Q86VN1|VPS36_HUMAN	Vacuolar protein-sorting-associated protein 36 OS=Homo sapiens OX=9606 GN=VPS36 PE=1 SV=1	phosphatidylinositol-3-phosphate binding; protein C-terminus binding; ubiquitin binding; endosomal transport; macroautophagy; multivesicular body assembly; protein transport to vacuole involved in ubiquitin-dependent protein catabolic process via the multivesicular body sorting pathway	**0,001**	**6324,8**	**13900,4**	9997,8	6734,2	13474,7	1,14
sp|Q9UQ13|SHOC2_HUMAN	Leucine-rich repeat protein SHOC-2 OS=Homo sapiens OX=9606 GN=SHOC2 PE=1 SV=2	protein phosphatase 1 binding; protein phosphatase regulator activity; fibroblast growth factor receptor signaling pathway; MAPK cascade; positive regulation of Ras protein signal transduction; Ras protein signal transduction; signal transduction	**0,002**	**8455,5**	**18748,0**	11569,1	9299,4	17982,7	1,15
sp|P60953-1|CDC42_HUMAN	Isoform 1 of Cell division control protein 42 homolog OS=Homo sapiens OX=9606 GN=CDC42	G protein activity; GTPase activity; GTP binding; GTP-dependent protein binding; identical protein binding; mitogen-activated protein kinase kinase kinase binding; protein kinase binding; thioesterase binding; ubiquitin protein ligase activity; actin cytoskeleton organization; actin filament branching; actin filament organization; adherens junction organization; cardiac conduction system development; Cdc42 protein signal transduction; cell migration; cell projection assembly; cellular protein localization; cellular response to interferon-gamma; dendritic cell migration; dendritic spine morphogenesis; endocytosis; establishment of Golgi localization; establishment or maintenance of cell polarity; Fc-gamma receptor signaling pathway involved in phagocytosis; filopodium assembly; Golgi organization; heart contraction; integrin-mediated signaling pathway; modification of synaptic structure; negative regulation of epidermal growth factor receptor signaling pathway; negative regulation of protein-containing complex assembly; neuron fate determination; neuropilin signaling pathway; nuclear migration; organelle transport along microtubule; positive regulation of filopodium assembly; positive regulation of intracellular protein transport; positive regulation of lamellipodium assembly; positive regulation of muscle cell differentiation; positive regulation of neuron apoptotic process; positive regulation of stress fiber assembly; positive regulation of substrate adhesion-dependent cell spreading; positive regulation of synapse structural plasticity; regulation of attachment of spindle microtubules to kinetochore; viral RNA genome replication; Wnt signaling pathway, planar cell polarity pathway	**0,007**	**8005,6**	**18426,8**	8752,9	4504,4	21819,9	1,20
sp|Q5VWJ9|SNX30_HUMAN	Sorting nexin-30 OS=Homo sapiens OX=9606 GN=SNX30 PE=1 SV=1	Phosphatidylinositol binding; protein transport; intracellular trafficking	**0,002**	**6938,5**	**18207,1**	17019,6	7531,6	18044,3	1,39

sp|Q96M27|PRRC1_HUMAN	Protein PRRC1 OS=Homo sapiens OX=9606 GN=PRRC1 PE=1 SV=1	Identical protein binging; activation of PKA activity	**0,003**	**3420,4**	**9135,2**	10360,5	3245,2	8698,4	1,42
sp|Q9Y4P1-2|ATG4B_HUMAN	Isoform 2 of Cysteine protease ATG4B OS=Homo sapiens OX=9606 GN=ATG4B	cysteine-type endopeptidase activity; autophagy; macroautophagy; positive regulation of protein catabolic process; protein delipidation; protein transport; proteolysis	**0,003**	**971,0**	**2853,9**	1851,7	745,8	3439,5	1,56
sp|Q86W56-4|PARG_HUMAN	Isoform 4 of Poly(ADP-ribose) glycohydrolase OS=Homo sapiens OX=9606 GN=PARG	poly(ADP-ribose) glycohydrolase activity; ATP generation from poly-ADP-D-ribose; base-excision repair, gap-filling; carbohydrate metabolic process; nucleotide-sugar metabolic process; regulation of DNA repair; regulation of histone modification	**0,006**	**6888,5**	**21685,1**	8766,8	2726,1	24338,9	1,65
sp|Q7Z3J2|VP35L_HUMAN	VPS35 endosomal protein sorting factor-like OS=Homo sapiens OX=9606 GN=VPS35L PE=1 SV=2	endocytic recycling; Golgi to plasma membrane transport; neutrophil degranulation; protein transport	**0,005**	**2641,4**	**6463,8**	6618,5	2207,2	6745,8	1,29
sp|P10909-5|CLUS_HUMAN	Isoform 5 of Clusterin OS=Homo sapiens OX=9606 GN=CLU	amyloid-beta binding; chaperone binding; low-density lipoprotein particle receptor binding; misfolded protein binding; protein-containing complex binding; protein heterodimerization activity; signaling receptor binding; tau protein binding; ubiquitin protein ligase binding; unfolded protein binding; antimicrobial humoral response; cell morphogenesis; central nervous system myelin maintenance; chaperone-mediated protein complex assembly; chaperone-mediated protein folding; complement activation; immune response;regulation of amyloid-beta formation; regulation of neuronal signal transduction; regulation of neuron death; release of cytochrome c from mitochondria	**0,008**	**51063,8**	**119029,0**	79311,2	50662,5	117686,8	1,22
sp|P07942|LAMB1_HUMAN	Laminin subunit beta-1 OS=Homo sapiens OX=9606 GN=LAMB1 PE=1 SV=2	Cell Adhesion; cell migration; extracellular matrix organization; neuronprojection development; neuron-glial interaction involved in cerebral cortex radial migration	**0,003**	**24084,1**	**75103,3**	50825,5	25640,4	93515,5	1,64
**DOWNREGULATED**									
**PROTEIN**	**GROUP AND RELATED GENE (GN)**	**GO: BIOLOGICAL FUNCTION**	**p-value**	**Protein Mean Peak Intensity**					**Log$_2$FC FRDA/CT**
				**Mean CT**	**Mean FRDA**	**Mean ISO CT**	**Median CT**	**Median FRDA**	
sp|Q02539|H11_HUMAN	Histone H1.1 OS=Homo sapiens OX=9606 GN=HIST1H1A PE=1 SV=3	chromosome condensation; negative regulation of chromatin silencing; negative regulation of DNA recombination; chromatin binding; double-stranded DNA binding	**0,000**	**162206,4**	**14742,2**	7956,5	152890,7	12918,8	-3,46
sp|Q96S19-5|MTL26_HUMAN	Isoform 5 of Methyltransferase-like 26 OS=Homo sapiens OX=9606 GN=METTL26	methyltransferase	**0,004**	**26797,8**	**6447,1**	6426,1	26698,2	1487,3	-2,06
sp|P54198-2|HIRA_HUMAN	Isoform Short of Protein HIRA OS=Homo sapiens OX=9606 GN=HIRA	histone binding; RNA polymerase II transcription factor binding; transcription corepressor activity anatomical structure morphogenesis; DNA replication-independent nucleosome assembly; regulation of transcription by RNA polymerase II; transcription, DNA-templated	**0,004**	**8658,0**	**3299,6**	2955,7	7770,3	2667,1	-1,39
sp|P52926|HMGA2_HUMAN	High mobility group protein HMGI-C OS=Homo sapiens OX=9606 GN=HMGA2 PE=1 SV=1	DNA binding; nucleosomal binding; SMAD binding; transcription coregulator activity; transcription corepressor activity; transcription factor binding; transcription regulatory region sequence-specific DNA binding; base-excision repair; cell division; chondrocyte differentiation; chromatin organization; chromosome breakage; chromosome condensation; DNA damage response, detection of DNA damage; endodermal cell differentiation; epithelial to mesenchymal transition; fat cell differentiation; heterochromatin assembly; histone H2A-S139 phosphorylation; mesenchymal cell differentiation; mesodermal cell differentiation; mesodermal-endodermal cell signaling; mitotic G2 DNA damage checkpoint; multicellular organism development; negative regulation by host of viral transcription; negative regulation of apoptotic process; negative; regulation of cellular senescence; negative regulation of DNA binding; negative regulation of double-strand break repair via nonhomologous end joining; negative regulation of single stranded viral RNA replication via double stranded DNA intermediate; negative regulation of transcription, DNA-templated; negative regulation of transcription by RNA polymerase II; oncogene-induced cell senescence; positive regulation of angiogenesis; positive regulation of apoptotic process; positive regulation of cell proliferation in bone marrow; positive regulation of cellular response to X-ray; positive regulation of cellular senescence; positive regulation of gene expression; positive regulation of protein serine/threonine kinase activity; positive regulation of response to DNA damage stimulus; positive regulation of stem cell proliferation; positive regulation of transcription, DNA-templated; positive regulation of transcription by RNA polymerase II; regulation of cell cycle process; regulation of cellular response to drug; regulation of growth regulation of stem cell population maintenance; regulation of transcription, DNA-templated; response to virus; senescence-associated heterochromatin focus assembly; stem cell differentiation	**0,000**	**94730,2**	**37407,7**	54202,3	79679,0	34183,4	-1,34
sp|Q6BDS2|URFB1_HUMAN	UHRF1-binding protein 1 OS=Homo sapiens OX=9606 GN=UHRF1BP1 PE=1 SV=1	histone deacetylase binding; identical protein binding; may act as a negative regulator of cell growth	**0,008**	**3695,0**	**1768,2**	2984,7	3780,3	1514,2	-1,06
sp|Q9H0H0|INT2_HUMAN	Integrator complex subunit 2 OS=Homo sapiens OX=9606 GN=INTS2 PE=1 SV=2	snRNA 3'-end processing; snRNA processing; snRNA transcription by RNA polymerase II	**0,002**	**5561,2**	**2767,0**	3406,5	5858,2	2663,4	-1,01
sp|P09382|LEG1_HUMAN	Galectin-1 OS=Homo sapiens OX=9606 GN=LGALS1 PE=1 SV=2	carbohydrate binding; identical protein binding; lactose binding; laminin binding; RNA binding apoptotic process; cellular protein metabolic process; cellular response to glucose stimulus; cellular response to organic cyclic compound; myoblast differentiation; negative regulation of cell-substrate adhesion; negative regulation of neuron projection development; plasma cell differentiation; positive regulation of erythrocyte aggregation; positive regulation of I-kappaB kinase/NF-kappaB signaling; positive regulation of viral entry into host cell; post-translational protein modification; regulation of apoptotic process; response to axon injury; response to drug ; response to isolation stress; T cell costimulation	**0,000**	**42609,4**	**7614,2**	17767,9	43715,6	7357,6	-2,48
sp|P78417|GSTO1_HUMAN	Glutathione S-transferase omega-1 OS=Homo sapiens OX=9606 GN=GSTO1 PE=1 SV=2	glutathione dehydrogenase (ascorbate) activity; glutathione transferase activity; methylarsonate reductase activity; oxidoreductase activity cellular response to arsenic-containing substance; glutathione derivative biosynthetic process; glutathione metabolic process; interleukin-12-mediated signaling pathway; L-ascorbic acid metabolic process; methylation; negative regulation of ryanodine-sensitive calcium-release channel activity; positive regulation of ryanodine-sensitive calcium-release channel activity; positive regulation of skeletal muscle contraction by regulation of release of sequestered calcium ion; regulation of cardiac muscle contraction by regulation of the release of	**0,000**	**54244,4**	**12811,0**	41800,8	51628,2	11358,4	-2,08

		sequestered calcium ion; regulation of release of sequestered calcium ion into cytosol by sarcoplasmic reticulum; xenobiotic catabolic process							
sp|Q9NPA0|EMC7_HUMAN	ER membrane protein complex subunit 7 OS=Homo sapiens OX=9606 GN=EMC7 PE=1 SV=1	carbohydrate binding; protein insertion into ER membrane by stop-transfer membrane-anchor sequence; tail-anchored membrane protein insertion into ER membrane; part of the endoplasmic reticulum membrane protein complex (EMC) that enables the energy-independent insertion into endoplasmic reticulum membranes of newly synthesized membrane proteins	**0,000**	**98207,5**	**23769,4**	59332,4	106419,1	27504,3	-2,05
sp|P00338|LDHA_HUMAN	L-lactate dehydrogenase A chain OS=Homo sapiens OX=9606 GN=LDHA PE=1 SV=2	L-lactate dehydrogenase activity; carbohydrate metabolic process; carboxylic acid metabolic process	**0,000**	**728496,5**	**221896,9**	568446,7	728226,0	225622,6	-1,72
sp|Q01628|IFM3_HUMAN	Interferon-induced transmembrane protein 3 OS=Homo sapiens OX=9606 GN=IFITM3 PE=1 SV=2	defense response to virus; immune response; negative regulation of viral entry into host cell; negative regulation of viral genome replication; negative regulation of viral transcription; response to interferon-alpha; response to interferon-beta; response to interferon-gamma; response to virus; type I interferon signaling pathway	**0,001**	**6776,0**	**2249,2**	6132,4	6805,8	1148,2	-1,59
sp|O75056|SDC3_HUMAN	Syndecan-3 OS=Homo sapiens OX=9606 GN=SDC3 PE=1 SV=2	identical protein binding; cell migration; glycosaminoglycan biosynthetic process; glycosaminoglycan catabolic process; leukocyte migration; retinoid metabolic process; may have a role in the organization of cell shape by affecting the actin cytoskeleton, possibly by transferring signals from the cell surface in a sugar-dependent mechanism	**0,006**	**29250,0**	**11609,7**	6666,0	29752,7	11238,0	-1,33
sp|Q9H1A4|APC1_HUMAN	Anaphase-promoting complex subunit 1 OS=Homo sapiens OX=9606 GN=ANAPC1 PE=1 SV=1	molecular adaptor acticity; anaphase-promoting complex-dependent catabolic process; cell division; metaphase/anaphase transition of mitotic cell cycle; protein K11-linked ubiquitination; regulation of exit from mitosis; regulation of mitotic cell cycle phase transition; ubiquitin-dependent protein catabolic process	**0,001**	**13038,2**	**5830,0**	6525,8	13409,8	5737,5	-1,16
sp|Q9H497-2|TOR3A_HUMAN	Isoform 2 of Torsin-3A OS=Homo sapiens OX=9606 GN=TOR3A	ATP binding; ATPase activity	**0,004**	**9201,2**	**4179,3**	5398,0	8838,6	3972,4	-1,14
sp|P00491|PNPH_HUMAN	Purine nucleoside phosphorylase OS=Homo sapiens OX=9606 GN=PNP PE=1 SV=2	guanosine phosphorylase activity; dAMP catabolic process	**0,007**	**68926,3**	**34322,0**	40639,6	52149,7	34474,3	-1,01
sp|P48539|PCP4_HUMAN	Calmodulin regulator protein PCP4 OS=Homo sapiens OX=9606 GN=PCP4 PE=1 SV=3	calcium ion binding; calmodulin binding; calmodulin dependent kinase signaling pathway; positive regulation of neuron differentiation	**0,007**	**76299,6**	**33823,1**	38857,0	78335,5	41455,1	-1,17
sp|Q99584|S10AD_HUMAN	Protein S100-A13 OS=Homo sapiens OX=9606 GN=S100A13 PE=1 SV=1	calcium-dependent protein binding; calcium ion binding; copper ion binding; fibroblast growth factor binding; lipid binding; protein homodimerization activity; RAGE receptor binding; zinc ion binding; mast cell degranulation; positive regulation of cell population proliferation; positive regulation of cytokine production; positive regulation of I-kappaB kinase/NF-kappaB signaling; positive regulation of interleukin-1 alpha production; protein transport; regulation of cell shape; response to copper ion; response to electrical stimulus	**0,000**	**44553,1**	**16816,7**	42855,2	45934,5	17453,9	-1,41
sp|P04271|S100B_HUMAN	Protein S100-B OS=Homo sapiens OX=9606 GN=S100B PE=1 SV=2	Calcium-dependent protein binding; calcium ion binding; identical protein binding; protein homodimerization activity; RAGE receptor binding; S100 protein binding; tau protein binding; zinc ion binding axonogenesis; central nervous system development; innate immune response; learning or memory; positive regulation of cell population proliferation; positive regulation of I-kappaB kinase/NF-kappaB signaling	**0,000**	**4858,3**	**1181,2**	1452,5	5091,4	913,5	-2,04
sp|P07602-3|SAP_HUMAN	Isoform Sap-mu-9 of Prosaposin OS=Homo sapiens OX=9606 GN=PSAP	identical protein binding; phospholipid binding; protease binding; protein homodimerization activity; enzyme activator activity; adenylate cyclase-inhibiting G protein-coupled receptor signaling pathway; epithelial cell differentiation involved in prostate gland development; ganglioside GM1 transport to membrane; glycosphingolipid metabolic process; G protein-coupled receptor signaling pathway; lysosomal transport; neutrophil degranulation; platelet degranulation; positive regulation of beta-galactosidase activity; prostate gland growth; regulation of autophagy; regulation of lipid metabolic process	**0,008**	**121325,2**	**48654,8**	60125,4	122082,2	47824,0	-1,32
sp|Q9NR46|SHLB2_HUMAN	Endophilin-B2 OS=Homo sapiens OX=9606 GN=SH3GLB2 PE=1 SV=1	Cadherin binding; identical protein binding	**0,000**	**29053,1**	**6385,6**	7041,0	26340,0	4314,2	-2,19
sp|P07951-2|TPM2_HUMAN	Isoform 2 of Tropomyosin beta chain OS=Homo sapiens OX=9606 GN=TPM2	actin binding; actin filament binding; identical protein binding; protein heterodimerization activity; protein homodimerization activity; structural constituent of muscle; actin filament organization; muscle contraction ; muscle filament sliding; regulation of ATPase activity	**0,000**	**20198,9**	**5246,2**	12062,3	20606,7	3876,3	-1,94
sp|P20810-6|ICAL_HUMAN	Isoform 6 of Calpastatin OS=Homo sapiens OX=9606 GN=CAST	cadherin binding; calcium-dependent cysteine-type endopeptidase inhibitor activity; endopeptidase inhibitor activity; RNA binding inhibition of cysteine-type endopeptidase activity; negative regulation of type B; pancreatic cell apoptotic process; presynaptic active zone organization	**0,001**	**6050,0**	**1722,6**	4798,6	6818,9	860,0	-1,81
sp|O15143|ARC1B_HUMAN	Actin-related protein 2/3 complex subunit 1B OS=Homo sapiens OX=9606 GN=ARPC1B PE=1 SV=3	Arp2/3 complex-mediated actin nucleation; ephrin receptor signaling pathway; Fc-gamma receptor signaling pathway involved in phagocytosis; response to estradiol; response to estrogen; actin binding; structural constituent of cytoskeleton	**0,000**	**19370,0**	**5806,3**	9225,2	17823,9	5933,2	-1,74
sp|P05413|FABPH_HUMAN	Fatty acid-binding protein, heart OS=Homo sapiens OX=9606 GN=FABP3 PE=1 SV=4	cytoskeletal protein binding; icosatetraenoic acid binding; long-chain fatty acid binding; long-chain fatty acid transporter activity; oleic acid binding brown fat cell differentiation; cholesterol homeostasis; fatty acid metabolic process; intracellular lipid transport; long-chain fatty acid transport; negative regulation of cell population proliferation; phospholipid homeostasis; positive regulation of long-chain fatty acid import into cell; positive regulation of phospholipid biosynthetic process; regulation of fatty acid oxidation; regulation of phosphatidylcholine biosynthetic process; response to drug; response to fatty acid; response to insulin; triglyceride catabolic process	**0,000**	**35322,0**	**11533,1**	9372,6	33570,8	9986,9	-1,61
sp|P13797|PLST_HUMAN	Plastin-3 OS=Homo sapiens OX=9606 GN=PLS3 PE=1 SV=4	actin filament binding; calcium ion binding actin filament bundle assembly; actin filament network formation; bone development	**0,000**	**128506,5**	**51227,6**	150688,1	146194,4	48804,9	-1,33
sp|Q05682-5|CALD1_HUMAN	Isoform 5 of Caldesmon OS=Homo sapiens OX=9606 GN=CALD1	actin binding; cadherin binding; calmodulin binding; myosin binding; tropomyosin binding; actin filament bundle assembly; angiogenesis; muscle contraction	**0,000**	**31728,9**	**13931,4**	29765,1	28902,0	12843,1	-1,19
sp|P09493-4|TPM1_HUMAN	Isoform 4 of Tropomyosin alpha-1 chain OS=Homo sapiens OX=9606 GN=TPM1	actin binding; actin filament binding ; cytoskeletal protein binding; identical protein binding; protein heterodimerization activity; protein homodimerization activity; structural constituent of cytoskeleton; structural constituent of muscle	**0,000**	**59520,2**	**26441,3**	32966,9	58235,6	27690,7	-1,17
sp|P06396-2|GELS_HUMAN	Isoform 2 of Gelsolin OS=Homo sapiens OX=9606 GN=GSN	actin binding; calcium ion binding; myosin II binding; phosphatidylinositol 3-kinase catalytic subunit binding; phosphatidylinositol-4,5-bisphosphate binding; actin filament capping; actin filament depolymerization; actin filament polymerization; actin filament reorganization; actin filament severing; actin nucleation; aging; amyloid fibril formation; cardiac muscle cell contraction; cell projection	**0,000**	**108015,2**	**50728,5**	100901,0	100845,1	52184,5	-1,09

		assembly; cellular response to cadmium ion; cellular response to interferon-gamma; central nervous system development; cilium assembly; negative regulation of viral entry into host cell; neutrophil degranulation; oligodendrocyte development; phagocytosis, engulfment; phosphatidylinositol-mediated signaling; positive regulation of actin nucleation; positive regulation of cysteine-type endopeptidase activity involved in apoptotic signaling pathway; positive regulation of gene expression; positive regulation of keratinocyte apoptotic process; positive regulation of protein processing in phagocytic vesicle; protein destabilization; regulation of cell adhesion; regulation of establishment of T cell polarity; regulation of plasma membrane raft polarization; regulation of podosome assembly; regulation of receptor clustering; relaxation of cardiac muscle; renal protein absorption; response to ethanol; response to folic acid; response to muscle stretch; sequestering of actin monomers; striated muscle atrophy; tissue regeneration; wound healing							
sp|P02545|LMNA_HUMAN	Prelamin-A/C OS=Homo sapiens OX=9606 GN=LMNA PE=1 SV=1	identical protein binding; structural molecule activity; cellular protein localization; cellular response to hypoxia; DNA double-strand break attachment to nuclear envelope; establishment or maintenance of microtubule; cytoskeleton polarity; IRE1-mediated unfolded protein response; mitotic nuclear envelope reassembly; muscle organ development; negative regulation of cardiac muscle hypertrophy in response to stress; negative regulation of cell population proliferation; nuclear envelope organization; positive regulation of cell aging; protein localization to nucleus; regulation of cell migration; regulation of telomere maintenance; nuclear assembly, chromatin organization, nuclear membrane and telomere dynamics; required for normal development of peripheral nervous system and skeletal muscle and for muscle satellite cell proliferation	**0,000**	**50695,3**	**24063,7**	24589,4	46463,5	23290,2	-1,07
sp|Q9NUM4|T106B_HUMAN	Transmembrane protein 106B OS=Homo sapiens OX=9606 GN=TMEM106B PE=1 SV=2	dendrite morphogenesis and branching; lysosomal transport; lysosome localization; lysosome organization	**0,003**	**11092,9**	**5332,1**	7955,7	10581,2	4901,5	-1,06
sp|Q15063-5|POSTN_HUMAN	Isoform 5 of Periostin OS=Homo sapiens OX=9606 GN=POSTN	cell adhesion molecule binding; heparin binding; metal ion binding; bone regeneration; cell adhesion; cellular response to fibroblast growth factor stimulus; cellular response to transforming growth factor beta; cellular response to tumor necrosis factor; cellular response to vitamin K; extracellular matrix organization; negative regulation of cell-matrix adhesion; negative regulation of substrate adhesion-dependent cell spreading; neuron projection extension; positive regulation of chemokine (C-X-C motif) ligand 2 production; positive regulation of smooth muscle cell migration; regulation of Notch signaling pathway; regulation of systemic arterial blood pressure; response to hypoxia; response to mechanical stimulus; response to muscle activity; tissue development; wound healing	**0,000**	**52190,0**	**25267,4**	83386,8	52547,3	18971,6	-1,05
sp|O43491-2|E41L2_HUMAN	Isoform 2 of Band 4.1-like protein 2 OS=Homo sapiens OX=9606 GN=EPB41L2	actin binding; PH domain binding; spectrin binding; structural molecule activity; actomyosin structure organization; cell cycle; cell division; cortical actin cytoskeleton organization; positive regulation of protein localization to cell cortex	**0,000**	**34255,4**	**17115,9**	34051,0	34673,3	16833,9	-1,00
sp|O15427|MOT4_HUMAN	Monocarboxylate transporter 4 OS=Homo sapiens OX=9606 GN=SLC16A3 PE=1 SV=1	lactate transmembrane transporter activity; monocarboxylic acid transmembrane transporter activity; RNA binding; symporter activity leukocyte migration; monocarboxylic acid transport; plasma membrane lactate transport; pyruvate metabolic process	**0,001**	**16322,5**	**5038,0**	9766,9	16549,7	3905,9	-1,70
sp|Q96ER9|CCD51_HUMAN	Coiled-coil domain-containing protein 51 OS=Homo sapiens OX=9606 GN=CCDC51 PE=1 SV=2	mitochondrial ATP-gated potassium channel activity; potassium ion transmembrane transport; may contribute to the homeostatic control of cellular metabolism under stress conditions by regulating the mitochondrial matrix volume	**0,003**	**10002,8**	**4990,9**	6763,4	10016,5	4178,0	-1,00

DIFFERENTIALLY EXPRESSED PROTEINS – FRDA vs ISO CT

UPREGULATED

PROTEIN	GROUP AND RELATED GENE (GN)	GO: BIOLOGICAL FUNCTION	p-value	Protein Mean Peak Intensity					Log$_2$FC FRDA/ ISO CT
				Mean ISO CT	Mean FRDA	Mean CT	Median ISO CT	Median FRDA	
sp|Q96MU7-2|YTDC1_HUMAN	Isoform 2 of YTH domain-containing protein 1 OS=Homo sapiens OX=9606 GN=YTHDC1	dosage compensation by inactivation of X chromosome; mRNA export from the nucleus; mRNA splicing, via spliceosome; posttranscriptional regulation of gene expression	**0,000**	**3945,5**	**8453,7**	16266,7	4221,9	8767,0	1,10
sp|Q9HCJ6|VAT1L_HUMAN	Synaptic vesicle membrane protein VAT-1 homolog-like OS=Homo sapiens OX=9606 GN=VAT1L PE=1 SV=2	oxidoreductase activity; zinc ion binding	**0,000**	**75389,8**	**153541,5**	89811,6	85987,6	168354,1	1,03
sp|O43301|HS12A_HUMAN	Heat shock 70 kDa protein 12A OS=Homo sapiens OX=9606 GN=HSPA12A PE=1 SV=2	ATP binding	**0,000**	**57363,0**	**117513,2**	75095,8	54891,2	106579,2	1,03
sp|Q6PCE3|PGM2L_HUMAN	Glucose 1,6-bisphosphate synthase OS=Homo sapiens OX=9606 GN=PGM2L1 PE=1 SV=3	glucose-1,6-bisphosphate synthase activity; intramolecular transferase activity, phosphotransferases; galactose catabolic process; glucose 6-phosphate metabolic process; glucose metabolic process; glycogen biosynthetic process; glycogen catabolic process	**0,002**	**77243,3**	**158411,6**	129424,1	70848,5	145826,2	1,04
sp|P04040|CATA_HUMAN	Catalase OS=Homo sapiens OX=9606 GN=CAT PE=1 SV=3	cellular response to growth factor stimulus; cellular response to oxidative stress; cholesterol metabolic process; negative regulation of apoptotic process; response to hypoxia; antioxidant activity	**0,003**	**30772,1**	**63429,7**	54048,4	31104,6	61985,0	1,04
sp|Q05639|EF1A2_HUMAN	Elongation factor 1-alpha 2 OS=Homo sapiens OX=9606 GN=EEF1A2 PE=1 SV=1	positive regulation of apoptotic process; positive regulation of lipid kinase activity; regulation of chaperone-mediated autophagy; response to electrical stimulus; translation; translation elongation	**0,000**	**21916,3**	**45294,8**	32298,4	21960,5	48219,7	1,05
sp|P05067-3|A4_HUMAN	Isoform L-APP677 of Amyloid-beta precursor protein OS=Homo sapiens OX=9606 GN=APP	acetylcholine receptor activator activity; chaperone; chemoattractant activity; chromatin binding; DNA binding; enzyme binding; enzyme binding; G protein-coupled receptor binding; growth factor receptor binding; heparan sulfate binding; integrin binding; activation of MAPK activity; adult locomotory behavior; axo-dendritic transport; axon midline choice point recognition; axonogenesis; calcium-mediated signaling; cell adhesion; cellular copper ion homeostasis; cellular process; cellular protein metabolic process; cellular response to amyloid-beta; cellular response to cAMP; dendrite development; endocytosis; extracellular matrix organization; locomotory behavior; modulation of excitatory postsynaptic potential; negative regulation of gene expression; negative regulation of long-term synaptic potentiation; negative regulation of mitochondrion organization; negative regulation of neuron death; neuromuscular process controlling balance; neuron projection	**0,000**	**33268,8**	**69069,6**	79564,4	30510,9	67344,0	1,05

		development; neuron projection maintenance; neuron remodeling; synapse organization; synaptic growth at neuromuscular junction							
sp|P60953-1|CDC42_HUMAN	Isoform 1 of Cell division control protein 42 homolog OS=Homo sapiens OX=9606 GN=CDC42	G protein activity; GTPase activity; GTP binding; GTP-dependent protein binding; identical protein binding; mitogen-activated protein kinase kinase kinase binding; protein kinase binding; thioesterase binding; ubiquitin protein ligase activity; actin cytoskeleton organization; actin filament branching; actin filament organization; adherens junction organization; cardiac conduction system development; Cdc42 protein signal transduction; cell migration; cell projection assembly; cellular protein localization; cellular response to interferon-gamma; dendritic cell migration; dendritic spine morphogenesis; endocytosis; establishment of Golgi localization; establishment or maintenance of cell polarity; Fc-gamma receptor signaling pathway involved in phagocytosis; filopodium assembly; Golgi organization; heart contraction; integrin-mediated signaling pathway; modification of synaptic structure; negative regulation of epidermal growth factor receptor signaling pathway; negative regulation of protein-containing complex assembly; neuron fate determination; neuropilin signaling pathway; nuclear migration; organelle transport along microtubule; positive regulation of filopodium assembly; positive regulation of intracellular protein transport; positive regulation of lamellipodium assembly; positive regulation of muscle cell differentiation; positive regulation of neuron apoptotic process; positive regulation of stress fiber assembly; positive regulation of substrate adhesion-dependent cell spreading; positive regulation of synapse structural plasticity; regulation of attachment of spindle microtubules to kinetochore; viral RNA genome replication; Wnt signaling pathway, planar cell polarity pathway	**0,003**	**8752,9**	**18426,8**	8005,6	7597,3	21819,9	1,07
sp|Q9UHG2|PCSK1_HUMAN	ProSAAS OS=Homo sapiens OX=9606 GN=PCSK1N PE=1 SV=1	neuropeptide signaling pathway; endopeptidase inhibitor activity	**0,000**	**99290,2**	**219291,2**	169430,5	83346,9	236706,5	1,14
sp|Q15125|EBP_HUMAN	3-beta-hydroxysteroid-Delta(8),Delta(7)-isomerase OS=Homo sapiens OX=9606 GN=EBP PE=1 SV=3	cholesterol biosynthetic process; cholesterol metabolic process; skeletal system development	**0,002**	**4857,6**	**11475,3**	13202,2	4735,5	12372,6	1,24
sp|Q9UJX2|CDC23_HUMAN	Cell division cycle protein 23 homolog OS=Homo sapiens OX=9606 GN=CDC23 PE=1 SV=3	ubiquitin-protein transferase activity; anaphase-promoting complex-dependent catabolic process; cell division	**0,002**	**6416,0**	**15171,1**	12493,9	6898,5	15424,3	1,24
sp|Q86W56-4|PARG_HUMAN	Isoform 4 of Poly(ADP-ribose) glycohydrolase OS=Homo sapiens OX=9606 GN=PARG	poly(ADP-ribose) glycohydrolase activity; ATP generation from poly-ADP-D-ribose; base-excision repair, gap-filling; carbohydrate metabolic process; nucleotide-sugar metabolic process; regulation of DNA repair; regulation of histone modification	**0,004**	**8766,8**	**21685,1**	6888,5	3421,7	24338,9	1,31
sp|Q7L7X3|TAOK1_HUMAN	Serine/threonine-protein kinase TAO1 OS=Homo sapiens OX=9606 GN=TAOK1 PE=1 SV=1	tubulin binding; protein kinase activity; DNA repair; microtubule cytoskeleton organization; mitotic spindle organization; neuron projection morphogenesis; regulation of actin cytoskeleton organization; regulation of microtubule cytoskeleton organization	**0,004**	**25920,7**	**65189,6**	36856,9	18497,0	72282,0	1,33
sp|P17252|KPCA_HUMAN	Protein kinase C alpha type OS=Homo sapiens OX=9606 GN=PRKCA PE=1 SV=4	ATP binding; calcium-dependent protein kinase C activity; enzyme binding; integrin binding; protein kinase activity; apoptotic signaling pathway; axon guidance; cell adhesion; intracellular signal transduction; positive regulation of cardiac muscle hypertrophy; positive regulation of cell adhesion; positive regulation of ERK1 and ERK2 cascade	**0,002**	**5216,4**	**13320,6**	7554,7	4863,3	15984,1	1,35
sp|Q8WVI7|PPR1C_HUMAN	Protein phosphatase 1 regulatory subunit 1C OS=Homo sapiens OX=9606 GN=PPP1R1C PE=3 SV=1	protein phosphatase inhibitor activity; intracellular signal transduction; cell cycle; cell division	**0,001**	**14664,0**	**39211,4**	43879,7	10588,7	38670,9	1,42
sp|Q5RI15|COX20_HUMAN	Cytochrome c oxidase assembly protein COX20, mitochondrial OS=Homo sapiens OX=9606 GN=COX20 PE=1 SV=2	mitochondrial cytochrome c oxidase assembly	**0,009**	**2947,2**	**10087,9**	10517,7	1673,7	8871,8	1,78
sp|Q9UGI8|TES_HUMAN	Testin OS=Homo sapiens OX=9606 GN=TES PE=1 SV=1	cadherin binding; RNA binding; negative regulation of cell population proliferation; may play a role in cell adhesion, cell spreading and in the reorganization of the actin cytoskeleton	**0,000**	**10767,2**	**40885,9**	45960,6	9723,2	40036,8	1,92
sp|O14639-2|ABLM1_HUMAN	Isoform 2 of Actin-binding LIM protein 1 OS=Homo sapiens OX=9606 GN=ABLIM1	actin binding; cytolskeleton organization; animal organ morphogenesis	**0,001**	**7838,7**	**15892,2**	13327,7	7255,2	13520,5	1,02
sp|Q02410-2|APBA1_HUMAN	Isoform 2 of Amyloid-beta A4 precursor protein-binding family A member 1 OS=Homo sapiens OX=9606 GN=APBA1	axo-dendritic transport; cell adhesion; chemical synaptic transmission; gamma-aminobutyric acid secretion; glutamate secretion; intracellular protein transport; in utero embryonic development; locomotory behavior; multicellular organism growth; nervous system development; neurotransmitter transport; protein-containing complex assembly; regulation of gene expression; regulation of synaptic vesicle exocytosis	**0,009**	**1878,5**	**3828,4**	3679,8	1196,0	4287,1	1,03
sp|P07196|NFL_HUMAN	Neurofilament light polypeptide OS=Homo sapiens OX=9606 GN=NEFL PE=1 SV=3	protein binding; phospholipase binding; structural constituent of cytoskeleton; structural constituent of postsynaptic intermediate filament cytoskeleton; anterograde axonal transport; axonal transport of mitochondrion; intermediate filament organization; intermediate filament polymerization or depolymerization; locomotion; MAPK cascade; microtubule cytoskeleton organization; negative regulation of neuron apoptotic process; neurofilament bundle assembly; neurofilament cytoskeleton organization; neuromuscular process controlling balance; neuron projection morphogenesis; peripheral nervous system axon regeneration; positive regulation of axonogenesis; protein polymerization; regulation of axon diameter; regulation of NMDA receptor activity; response to acrylamide; response to corticosterone; response to peptide hormone; response to sodium arsenite; response to toxic substance; retrograde axonal transport; spinal cord development; synapse maturation	**0,001**	**2300996,1**	**4732646,2**	7321839,6	1986276,0	3747057,2	1,04
sp|Q12860|CNTN1_HUMAN	Contactin-1 OS=Homo sapiens OX=9606 GN=CNTN1 PE=1 SV=1	carbohydrate binding; cell adhesion; cerebellum development; neuron projection development; positive regulation of sodium ion transport	**0,000**	**97306,6**	**202911,5**	165656,2	106492,8	209852,8	1,06
sp|Q9UDT6|CLIP2_HUMAN	CAP-Gly domain-containing linker protein 2 OS=Homo sapiens OX=9606 GN=CLIP2 PE=1 SV=1	cytoplasmic microtubule organization	**0,002**	**27022,7**	**56784,0**	49352,5	16534,7	56345,1	1,07
sp|P78310|CXAR_HUMAN	Coxsackievirus and adenovirus receptor OS=Homo sapiens OX=9606 GN=CXADR PE=1 SV=1	cell adhesion molecule binding; beta-catenin binding; actin cytoskeleton reorganization; AV node cell-bundle of His cell adhesion involved in cell communication; AV node cell to bundle of His cell communication; cardiac muscle fiber development; cell-cell junction organization; heart development; heterophilic cell-cell adhesion via plasma membrane cell adhesion molecules; homotypic cell-cell adhesion; regulation of AV node cell action potential; mitochondrion organization	**0,000**	**121187,0**	**255101,9**	188907,3	123193,0	249588,0	1,07
sp|Q9Y6R0|NUMBL_HUMAN	Numb-like protein OS=Homo sapiens OX=9606 GN=NUMBL PE=1 SV=1	adherens junction organization; axonogenesis; cytokine-mediated signaling pathway; lateral ventricle development; nervous system development; positive regulation of neurogenesis	**0,003**	**9418,8**	**20118,9**	17084,1	7112,2	20772,1	1,09

sp|P31146|COR1A_HUMAN	Coronin-1A OS=Homo sapiens OX=9606 GN=CORO1A PE=1 SV=4	actin binding; actin filament binding; actin monomer binding; cytoskeletal protein binding; myosin heavy chain binding; actin cytoskeleton organization; actin filament organization; calcium ion transport; cell-substrate adhesion; negative regulation of neuron apoptotic process; negative regulation of vesicle fusion	**0,000**	**49341,0**	**107414,9**	92671,3	39579,1	119923,2	1,12
sp|Q93045-2|STMN2_HUMAN	Isoform 2 of Stathmin-2 OS=Homo sapiens OX=9606 GN=STMN2	calcium-dependent protein binding; tubulin binding; cellular response to nerve growth factor stimulus; microtubule depolymerization; negative regulation of microtubule depolymerization; negative regulation of microtubule polymerization; negative regulation of neuron projection development; neuron projection development; positive regulation of microtubule depolymerization; positive regulation of neuron projection development; regulation of microtubule polymerization or depolymerization	**0,000**	**19646,8**	**43423,5**	23141,8	19731,8	41561,3	1,14
sp|Q9H4G0|E41L1_HUMAN	Band 4.1-like protein 1 OS=Homo sapiens OX=9606 GN=EPB41L1 PE=1 SV=2	actin binding; structural molecule activity; actomyosin structure organization; actin cytoskeleton organization	**0,000**	**37100,4**	**84858,3**	57427,8	35669,8	76778,8	1,19
sp|Q99719|SEPT5_HUMAN	Septin-5 OS=Homo sapiens OX=9606 GN=SEPTIN5 PE=1 SV=1	GTPase activity;structure molecule activity; adult behavior; cellular protein localization; cytoskeleton-dependent cytokinesis; regulation of exocytosis; regulation of synaptic vesicle exocytosis; social behavior; synaptic vesicle targeting	**0,000**	**57280,1**	**131721,2**	110599,9	58600,3	129382,9	1,20
sp|Q6UXK2|ISLR2_HUMAN	Immunoglobulin superfamily containing leucine-rich repeat protein 2 OS=Homo sapiens OX=9606 GN=ISLR2 PE=2 SV=1	positive regulation of axon extension	**0,000**	**43179,1**	**100245,9**	61334,5	49339,3	94547,6	1,22
sp|P10636-8|TAU_HUMAN	Isoform Tau-F of Microtubule-associated protein tau OS=Homo sapiens OX=9606 GN=MAPT	actin binding; DNA binding; enzyme binding; RNA binding; microtubule binding; axonal transport; axonal transport of mitochondrion; axon development; cell-cell signaling; central nervous system neuron development; cytoplasmic microtubule organization; generation of neurons; intracellular distribution of mitochondria; microtubule cytoskeleton organization; neuron projection development; positive regulation of axon extension; positive regulation of microtubule polymerization; positive regulation of neuron death; positive regulation of protein localization to synapse; positive regulation of superoxide anion generation; regulation of long-term synaptic depression; regulation of microtubule cytoskeleton organization; regulation of microtubule polymerization; regulation of mitochondrial fission; regulation of synaptic plasticity; synapse organization	**0,000**	**397463,6**	**923887,1**	660284,3	297999,9	800202,7	1,22
sp|Q06481-4|APLP2_HUMAN	Isoform 4 of Amyloid-like protein 2 OS=Homo sapiens OX=9606 GN=APLP2	DNA binding; protein binding; cellular protein metabolic process; G protein-coupled receptor signaling pathway; post-translational protein modification	**0,005**	**5299,8**	**12366,1**	11731,2	3580,6	12767,6	1,22
sp|Q9HCM2|PLXA4_HUMAN	Plexin-A4 OS=Homo sapiens OX=9606 GN=PLXNA4 PE=1 SV=4	semaphorin receptor activity; negative regulation of cell adhesion; positive regulation of axonogenesis; regulation of axon extension involved in axon guidance; regulation of cell migration; regulation of cell shape; semaphorin-plexin signaling pathway involved in axon guidance	**0,001**	**25731,0**	**62063,4**	64963,2	29293,1	48946,9	1,27
sp|Q13740|CD166_HUMAN	CD166 antigen OS=Homo sapiens OX=9606 GN=ALCAM PE=1 SV=2	protein binding; axon extension involved in axon guidance; cell adhesion; heterophilic cell-cell adhesion via plasma membrane cell adhesion molecules; neuron projection extension; signal transduction	**0,006**	**53443,3**	**144044,7**	237315,0	53646,2	96957,5	1,43
sp|O15394|NCAM2_HUMAN	Neural cell adhesion molecule 2 OS=Homo sapiens OX=9606 GN=NCAM2 PE=1 SV=2	identical protein binding; axonal fasciculation; neuron cell-cell adhesion	**0,001**	**27533,9**	**85653,3**	67221,9	25818,9	69555,9	1,64
sp|O00203-3|AP3B1_HUMAN	Isoform 2 of AP-3 complex subunit beta-1 OS=Homo sapiens OX=9606 GN=AP3B1	GTP-dependent protein binding; anterograde axonal transport; anterograde synaptic vesicle transport; establishment of protein localization to mitochondrial membrane involved in mitochondrial fission; mRNA transcription by RNA polymerase II	**0,002**	**54492,8**	**188646,5**	233981,0	36883,8	173241,8	1,79
sp|Q86YM7|HOME1_HUMAN	Homer protein homolog 1 OS=Homo sapiens OX=9606 GN=HOMER1 PE=1 SV=2	G protein-coupled glutamate receptor binding; ion channel binding; positive regulation of calcium ion transport; positive regulation of signal transduction; regulation of dendritic spine maintenance; regulation of postsynaptic neurotransmitter receptor activity; regulation of store-operated calcium entry; regulation of synaptic transmission, glutamatergic; response to calcium ion; skeletal muscle contraction; skeletal muscle fiber development	**0,002**	**9597,6**	**19589,1**	13986,9	10032,5	19667,5	1,03
sp|P13987|CD59_HUMAN	CD59 glycoprotein OS=Homo sapiens OX=9606 GN=CD59 PE=1 SV=1	complement binding; cell surface receptor signaling pathway; endoplasmic reticulum to Golgi vesicle-mediated transport	**0,000**	**20512,1**	**42075,7**	43373,9	14890,7	40261,9	1,04
sp|O43854|EDIL3_HUMAN	EGF-like repeat and discoidin I-like domain-containing protein 3 OS=Homo sapiens OX=9606 GN=EDIL3 PE=1 SV=1	calcium ion binding; integrin binding; cell adhesion; multicellular organism development; positive regulation of cell-substrate adhesion	**0,000**	**288180,5**	**599921,7**	502832,4	291210,4	572882,1	1,06
sp|Q99567|NUP88_HUMAN	Nuclear pore complex protein Nup88 OS=Homo sapiens OX=9606 GN=NUP88 PE=1 SV=2	structural consituent of nuclear pore; transporter activity; regulation of gene silencing by miRNA; mRNA export from nucleus; protein import into nucleus; ribosomal large subunit export from nucleus; ribosomal small subunit export from nucleus	**0,008**	**1249,6**	**2956,2**	3302,8	727,1	3158,5	1,24
sp|P60880|SNP25_HUMAN	Synaptosomal-associated protein 25 OS=Homo sapiens OX=9606 GN=SNAP25 PE=1 SV=1	voltage-gated potassium channel activity; calcium-dependent protein binding; exocytic insertion of neurotransmitter receptor to postsynaptic membrane; locomotory behavior; long-term synaptic potentiation; neurotransmitter receptor internalization; neurotransmitter secretion; neurotransmitter transport; neurotransmitter uptake; regulation of neuron projection development; synaptic vesicle exocytosis; synaptic vesicle fusion to presynaptic active zone membrane	**0,000**	**71406,0**	**154173,7**	105881,6	68096,2	139767,8	1,11
sp|Q13555-6|KCC2G_HUMAN	Isoform 6 of Calcium/calmodulin-dependent protein kinase type II subunit gamma OS=Homo sapiens OX=9606 GN=CAMK2G	nervous system development; regulation of calcium ion transport; regulation of neuron projection development; regulation of skeletal muscle adaptation	**0,003**	**9449,8**	**20862,8**	24381,5	9497,4	21467,5	1,14
sp|P05937|CALB1_HUMAN	Calbindin OS=Homo sapiens OX=9606 GN=CALB1 PE=1 SV=2	calcium ion binding involved in regulation of pre- and post-synaptic cytosolic calcium ion concentration; locomotory behavior; long-term memory; regulation of cytosolic calcium ion concentration; regulation of long-term synaptic potentiation	**0,007**	**28268,5**	**66681,0**	55406,1	17414,9	71872,2	1,24
sp|O60641|AP180_HUMAN	Clathrin coat assembly protein AP180 OS=Homo sapiens OX=9606 GN=SNAP91 PE=1 SV=2	1-phosphatidylinositol binding; clathrin heavy chain binding; clathrin coat assembly; clathrin-dependent endocytosis; protein transport; regulation of clathrin-dependent endocytosis; synaptic vesicle budding from presynaptic endocytic zone membrane; vesicle budding from membrane	**0,003**	**21985,7**	**52741,6**	55052,5	19703,7	48265,5	1,26
sp|P21579|SYT1_HUMAN	Synaptotagmin-1 OS=Homo sapiens OX=9606 GN=SYT1 PE=1 SV=1	calcium-dependent protein binding; calcium ion binding; calcium ion sensor activity; calmodulin binding; clathrin binding; brain development; calcium-dependent activation of synaptic vesicle fusion; calcium-ion regulated exocytosis; calcium ion-regulated exocytosis of neurotransmitter; chemical synaptic transmission; positive regulation of dendrite extension; positive regulation of synaptic transmission; regulation of synaptic transmission, glutamatergic; spontaneous neurotransmitter secretion; synaptic vesicle endocytosis; vesicle-mediated transport	**0,000**	**85024,8**	**222114,8**	146817,8	80410,7	210481,1	1,39
sp|O43761|SNG3_HUMAN	Synaptogyrin-3 OS=Homo sapiens OX=9606 GN=SYNGR3 PE=1 SV=2	positive regulation of transporter activity; regulated exocytosis	**0,000**	**752,7**	**2269,3**	2530,7	800,3	2115,4	1,59

sp|O43581-4|SYT7_HUMAN	Isoform 4 of Synaptotagmin-7 OS=Homo sapiens OX=9606 GN=SYT7	calcium-dependent phospholipid binding; calcium ion binding; calmodulin binding; clathrin binding; calcium ion-regulated exocytosis of neurotransmitter; calcium ion regulated lysosome exocytosis; plasma membrane repair; synaptic vesicle recycling; vesicle-mediated transport	**0,002**	**1668,9**	**5949,6**	4496,1	1472,3	6035,5	1,83
**DOWNREGULATED**									
**PROTEIN**	**GROUP AND RELATED GENE (GN)**	**GO: BIOLOGICAL FUNCTION**	**p-value**	**Protein Mean Peak Intensity**					**Log$_2$FC FRDA/ISO CT**
				**Mean ISO CT**	**Mean FRDA**	**Mean CT**	**Median ISO CT**	**Median FRDA**	
sp|Q9UPT8|ZC3H4_HUMAN	Zinc finger CCCH domain-containing protein 4 OS=Homo sapiens OX=9606 GN=ZC3H4 PE=1 SV=3	DNA-binding transcription factor activity, RNA polymerase II-specific; metal ion binding; RNA binding	**0,000**	**7219,2**	**1992,1**	1752,3	9209,7	1779,8	-1,86
sp|Q96EK4|THA11_HUMAN	THAP domain-containing protein 11 OS=Homo sapiens OX=9606 GN=THAP11 PE=1 SV=2	regulation of transcription by RNA polymerase II; DNA binding; DNA-binding transcription factor activity, RNA polymerase II-specific; DNA-binding transcription repressor activity, RNA polymerase II-specific; RNA polymerase II cis-regulatory region sequence-specific DNA binding; zinc ion binding	**0,001**	**6104,8**	**2096,7**	3061,8	5267,6	1601,4	-1,54
sp|Q12849|GRSF1_HUMAN	G-rich sequence factor 1 OS=Homo sapiens OX=9606 GN=GRSF1 PE=1 SV=3	mRNA binding; anterior/posterior pattern specification; morphogenesis of embryonic epithelium; mRNA polyadenylation; positive regulation of mitochondrial RNA catabolic process; regulation of RNA splicing; tRNA processing	**0,002**	**3080,5**	**1266,3**	1091,3	2875,0	948,6	-1,28
sp|O95347|SMC2_HUMAN	Structural maintenance of chromosomes protein 2 OS=Homo sapiens OX=9606 GN=SMC2 PE=1 SV=2	chromatin binding; single-stranded DNA binding; ATP binding; ATPase activity; cell division; kinetochore organization; meiotic chromosome condensation; meiotic chromosome segregation; mitotic chromosome condensation	**0,005**	**33131,1**	**13658,1**	16917,0	20526,9	12703,2	-1,28
sp|Q9UPN6|SCAF8_HUMAN	SR-related and CTD-associated factor 8 OS=Homo sapiens OX=9606 GN=SCAF8 PE=1 SV=1	mRNA polyadenylation; negative regulation of termination of RNA polymerase II transcription, poly(A)-coupled; positive regulation of DNA-templated transcription, elongation; termination of RNA polymerase II transcription	**0,005**	**6800,5**	**2912,6**	4955,3	5924,8	2149,4	-1,22
sp|Q12996|CSTF3_HUMAN	Cleavage stimulation factor subunit 3 OS=Homo sapiens OX=9606 GN=CSTF3 PE=1 SV=1	mRNA binding; mRNA splicing; mRNA cleavage	**0,000**	**33377,1**	**14846,0**	14948,7	38310,5	13344,9	-1,17
sp|Q9BZ95-4|NSD3_HUMAN	Isoform 4 of Histone-lysine N-methyltransferase NSD3 OS=Homo sapiens OX=9606 GN=NSD3	histone methylation; regulation of transcription	**0,007**	**5253,3**	**2477,1**	1777,6	6421,0	2959,2	-1,08
sp|Q04206|TF65_HUMAN	Transcription factor p65 OS=Homo sapiens OX=9606 GN=RELA PE=1 SV=2	regulation of transcription; regulation of apoptotic process; regulation of protein catabolic process; response to muscle stretch	**0,005**	**5156,7**	**2460,3**	2291,6	5285,2	2055,6	-1,07
sp|P84243|H33_HUMAN	Histone H3.3 OS=Homo sapiens OX=9606 GN=H3F3A PE=1 SV=2	regulation of gene expression; nucleasome assembly; regulation of gene silcencing by miRNA; muscle cell differentiation; negative regulation of chromosome condensation	**0,009**	**594098,9**	**287200,1**	214567,1	697003,4	312905,9	-1,05
sp|Q9C005|DPY30_HUMAN	Protein dpy-30 homolog OS=Homo sapiens OX=9606 GN=DPY30 PE=1 SV=1	chromatin silencing at telomere; endosomal transport; histone H3-K4 methylation	**0,000**	**34138,8**	**17047,7**	20985,8	36409,2	18642,1	-1,00
sp|P35251|RFC1_HUMAN	Replication factor C subunit 1 OS=Homo sapiens OX=9606 GN=RFC1 PE=1 SV=4	base-excision repair, gap-filling; DNA clamp unloading; DNA damage response, detection of DNA damage; DNA-dependent DNA replication	**0,009**	**5027,6**	**2286,0**	1961,6	5306,0	1794,9	-1,14
sp|Q9BPX3|CND3_HUMAN	Condensin complex subunit 3 OS=Homo sapiens OX=9606 GN=NCAPG PE=1 SV=1	cell division; mitotic chromosome condensation	**0,008**	**25872,6**	**12065,7**	5220,9	26157,9	10559,9	-1,10
sp|Q9H773|DCTP1_HUMAN	dCTP pyrophosphatase 1 OS=Homo sapiens OX=9606 GN=DCTPP1 PE=1 SV=1	dCTP catabolic process; DNA protection; nucleoside triphosphate catabolic process	**0,000**	**26644,2**	**12760,9**	13429,2	23715,3	13443,4	-1,06
sp|P24941|CDK2_HUMAN	Cyclin-dependent kinase 2 OS=Homo sapiens OX=9606 GN=CDK2 PE=1 SV=2	cell division; DNA repair; DNA replication; regulation of gene expression; histone phosphorylation; protein phosphorylation; signal transduction	**0,005**	**7986,1**	**3903,5**	5040,0	7434,9	2737,7	-1,03
sp|Q9H9T3|ELP3_HUMAN	Elongator complex protein 3 OS=Homo sapiens OX=9606 GN=ELP3 PE=1 SV=2	4 iron, 4 sulfur cluster binding; acetyltransferase activity; metal ion binding; N-acetyltransferase activity; phosphorylase kinase regulator activity; tRNA binding; central nervous system development; neuron migration;<br>positive regulation of cell migration; regulation of transcription by RNA polymerase II; tRNA uridine modification	**0,001**	**12866,3**	**5387,1**	10526,5	13666,2	5514,9	-1,26
sp|Q96PU8|QKI_HUMAN	Protein quaking OS=Homo sapiens OX=9606 GN=QKI PE=1 SV=1	long-chain fatty acid biosynthetic process; mRNA processing; mRNA transport; muscle cell differentiation; myelination; positive regulation of gene expression;regulation of mRNA splicing, via spliceosome; regulation of translation; RNA splicing	**0,008**	**13689,2**	**5991,9**	6472,7	16831,0	4707,9	-1,19
sp|P56545|CTBP2_HUMAN	C-terminal-binding protein 2 OS=Homo sapiens OX=9606 GN=CTBP2 PE=1 SV=1	chromatin binding; identical protein binding; NAD binding; oxidoreductase activity; negative regulation of transcription by RNA polymerase II; positive regulation of chromatin binding; synaptic vesicle docking	**0,006**	**25731,1**	**11960,5**	12653,4	28305,8	10288,4	-1,11
sp|P78417|GSTO1_HUMAN	Glutathione S-transferase omega-1 OS=Homo sapiens OX=9606 GN=GSTO1 PE=1 SV=2	glutathione dehydrogenase (ascorbate) activity; glutathione transferase activity; methylarsonate reductase activity; oxidoreductase activity cellular response to arsenic-containing substance; glutathione derivative biosynthetic process; glutathione metabolic process; interleukin-12-mediated signaling pathway; L-ascorbic acid metabolic process; methylation; negative regulation of ryanodine-sensitive calcium-release channel activity; positive regulation of ryanodine-sensitive calcium-release channel activity; positive regulation of skeletal muscle contraction by regulation of release of sequestered calcium ion; regulation of cardiac muscle contraction by regulation of the release of sequestered calcium ion; regulation of release of sequestered calcium ion into cytosol by sarcoplasmic reticulum; xenobiotic catabolic process	**0,000**	**41800,8**	**12811,0**	54244,4	52632,9	11358,4	-1,71
sp|Q5EB52|MEST_HUMAN	Mesoderm-specific transcript homolog protein OS=Homo sapiens OX=9606 GN=MEST PE=2 SV=2	hydrolase activity; regulation of lipid storage	**0,000**	**17592,1**	**5417,0**	9789,7	14071,4	5591,8	-1,70

sp|Q7Z4H3|HDDC2_HUMAN	HD domain-containing protein 2 OS=Homo sapiens OX=9606 GN=HDDC2 PE=1 SV=1	5'-deoxynucleotidase activity; metal ion binding	**0,006**	**16916,4**	**5552,1**	2785,5	13045,3	3963,6	-1,61
sp|O75976|CBPD_HUMAN	Carboxypeptidase D OS=Homo sapiens OX=9606 GN=CPD PE=1 SV=2	metallocarboxypeptidase activity; serine-type carboxypeptidase activity; zinc ion binding; peptide metabolic process; protein processing	**0,000**	**59589,1**	**20881,7**	37782,7	71819,7	17344,6	-1,51
sp|Q01628|IFM3_HUMAN	Interferon-induced transmembrane protein 3 OS=Homo sapiens OX=9606 GN=IFITM3 PE=1 SV=2	defense response to virus; immune response; negative regulation of viral entry into host cell; negative regulation of viral genome replication; negative regulation of viral transcription; response to interferon-alpha; response to interferon-beta; response to interferon-gamma; response to virus; type I interferon signaling pathway	**0,007**	**6132,4**	**2249,2**	6776,0	4602,2	1148,2	-1,45
sp|P00338|LDHA_HUMAN	L-lactate dehydrogenase A chain OS=Homo sapiens OX=9606 GN=LDHA PE=1 SV=2	L-lactate dehydrogenase activity; carbohydrate metabolic process; carboxylic acid metabolic process	**0,001**	**568446,7**	**221896,9**	728496,5	356305,2	225622,6	-1,36
sp|Q9NPH2|INO1_HUMAN	Inositol-3-phosphate synthase 1 OS=Homo sapiens OX=9606 GN=ISYNA1 PE=1 SV=1	inositol biosynthetic process; phospholipid biosynthetic process	**0,001**	**128507,3**	**50811,9**	65935,4	157117,1	48164,8	-1,34
sp|Q9NPA0|EMC7_HUMAN	ER membrane protein complex subunit 7 OS=Homo sapiens OX=9606 GN=EMC7 PE=1 SV=1	carbohydrate binding; protein insertion into ER membrane by stop-transfer membrane-anchor sequence; tail-anchored membrane protein insertion into ER membrane; part of the endoplasmic reticulum membrane protein complex (EMC) that enables the energy-independent insertion into endoplasmic reticulum membranes of newly synthesized membrane proteins	**0,008**	**59332,4**	**23769,4**	98207,5	43973,7	27504,3	-1,32
sp|Q8TF05-2|PP4R1_HUMAN	Isoform 2 of Serine/threonine-protein phosphatase 4 regulatory subunit 1 OS=Homo sapiens OX=9606 GN=PPP4R1	protein dephosphorylation; protein phosphorylation; signal transduction	**0,002**	**5013,9**	**2202,2**	772,1	5676,0	1692,9	-1,19
sp|P10415|BCL2_HUMAN	Apoptosis regulator Bcl-2 OS=Homo sapiens OX=9606 GN=BCL2 PE=1 SV=2	BH3 domain binding; channel activity; channel inhibitor activity; identical protein binding; protease binding; actin filament organization; apoptotic process; axonogenesis; axon regeneration; cell aging; cell-cell adhesion; cellular response to DNA damage stimulus; cellular response to glucose starvation; cellular response to hypoxia; extrinsic apoptotic signaling pathway in absence of ligand; extrinsic apoptotic signaling pathway via death domain receptors; focal adhesion assembly; negative regulation of apoptotic process ; negative regulation of apoptotic signaling pathway; negative regulation of autophagy; negative regulation of calcium ion transport into cytosol; negative regulation of cell growth; negative regulation of cell migration; negative regulation of mitochondrial depolarization; negative regulation of neuron apoptotic process; positive regulation of neuron maturation; reactive oxygen species metabolic process; regulation of calcium ion transport; regulation of cell-matrix adhesion; regulation of gene expression; regulation of glycoprotein biosynthetic process; regulation of mitochondrial membrane permeability; regulation of mitochondrial membrane potential	**0,003**	**4949,0**	**2180,9**	2347,1	6176,9	1731,0	-1,18
sp|Q15118|PDK1_HUMAN	[Pyruvate dehydrogenase (acetyl-transferring)] kinase isozyme 1, mitochondrial OS=Homo sapiens OX=9606 GN=PDK1 PE=1 SV=1	3-phosphoinositide-dependent protein kinase activity; apoptotic process; cell differentiation; cell surface receptor signaling pathway; positive regulation of cell growth; synaptic growth at neuromuscular junction	**0,009**	**17280,9**	**8028,8**	9184,7	17490,6	7620,5	-1,11
sp|Q06323|PSME1_HUMAN	Proteasome activator complex subunit 1 OS=Homo sapiens OX=9606 GN=PSME1 PE=1 SV=1	anaphase-promoting complex-dependent catabolic process; antigen processing; positive regulation of endopeptidase activity; positive regulation of canonical Wnt signaling pathway; regulation of mitotic cell cycle phase transition; regulation of mRNA stability; regulation of proteasomal protein catabolic process; transmembrane transport	**0,000**	**52119,8**	**24295,1**	38792,3	51167,0	24383,3	-1,10
sp|O00483|NDUA4_HUMAN	Cytochrome c oxidase subunit NDUFA4 OS=Homo sapiens OX=9606 GN=NDUFA4 PE=1 SV=1	mitochondrial electron transport, cytochrome c to oxygen; mitochondrial electron transport, NADH to ubiquinone; positive regulation of cytochrome-c oxidase activity; proton transmembrane transport	**0,000**	**71796,7**	**33872,1**	59137,1	68944,8	31637,4	-1,08
sp|Q8NFI3|ENASE_HUMAN	Cytosolic endo-beta-N-acetylglucosaminidase OS=Homo sapiens OX=9606 GN=ENGASE PE=1 SV=1	hydrolase activity, hydrolyzing O-glycosyl compounds; protein deglycosylation	**0,007**	**3286,7**	**1640,6**	3219,5	3014,1	1280,7	-1,00
sp|Q15063-5|POSTN_HUMAN	Isoform 5 of Periostin OS=Homo sapiens OX=9606 GN=POSTN	cell adhesion molecule binding; heparin binding; metal ion binding; bone regeneration; cell adhesion; cellular response to fibroblast growth factor stimulus; cellular response to transforming growth factor beta; cellular response to tumor necrosis factor; cellular response to vitamin K; extracellular matrix organization; negative regulation of cell-matrix adhesion; negative regulation of substrate adhesion-dependent cell spreading; neuron projection extension; positive regulation of chemokine (C-X-C motif) ligand 2 production; positive regulation of smooth muscle cell migration; regulation of Notch signaling pathway; regulation of systemic arterial blood pressure; response to hypoxia; response to mechanical stimulus; response to muscle activity; tissue development; wound healing	**0,001**	**83386,8**	**25267,4**	52190,0	99875,1	18971,6	-1,72
sp|P13797|PLST_HUMAN	Plastin-3 OS=Homo sapiens OX=9606 GN=PLS3 PE=1 SV=4	actin filament binding; calcium ion binding actin filament bundle assembly; actin filament network formation; bone development	**0,000**	**150688,1**	**51227,6**	128506,5	181379,2	48804,9	-1,56
sp|Q9Y490|TLN1_HUMAN	Talin-1 OS=Homo sapiens OX=9606 GN=TLN1 PE=1 SV=3	actin filament binding; cadherin binding; integrin binding; LIM domain binding; phosphatidylinositol binding; phosphatidylserine binding; structural constituent of cytoskeleton; vinculin binding; cell-cell adhesion; cell-cell junction assembly; cell-substrate junction assembly; cortical actin cytoskeleton organization; integrin activation; integrin-mediated signaling pathway; IRE1-mediated unfolded protein response; muscle contraction; platelet aggregation; platelet degranulation; viral process	**0,001**	**37101,1**	**12961,9**	18862,4	49559,7	13137,3	-1,52
sp|P20810-6|ICAL_HUMAN	Isoform 6 of Calpastatin OS=Homo sapiens OX=9606 GN=CAST	cadherin binding; calcium-dependent cysteine-type endopeptidase inhibitor activity; endopeptidase inhibitor activity; RNA binding inhibition of cysteine-type endopeptidase activity; negative regulation of type B; pancreatic cell apoptotic process; presynaptic active zone organization	**0,002**	**4798,6**	**1722,6**	6050,0	4572,8	860,0	-1,48
sp|Q05682-5|CALD1_HUMAN	Isoform 5 of Caldesmon OS=Homo sapiens OX=9606 GN=CALD1	actin binding; cadherin binding; calmodulin binding; myosin binding; tropomyosin binding; actin filament bundle assembly; angiogenesis; muscle contraction	**0,001**	**29765,1**	**13931,4**	31728,9	28676,5	12843,1	-1,10
sp|Q9BVC6|TM109_HUMAN	Transmembrane protein 109 OS=Homo sapiens OX=9606 GN=TMEM109 PE=1 SV=1	voltage-gated ion channel activity; intrinsic apoptotic signaling pathway in response to DNA damage by p53 class mediator; negative regulation of cell death; regulation of ion transmembrane transport	**0,001**	**57317,4**	**26796,1**	21339,2	59240,8	25369,8	-1,10

DIFFERENTIALLY EXPRESSED PROTEINS – ISO CT vs CT

UPREGULATED

PROTEIN	GROUP AND RELATED GENE (GN)	GO: BIOLOGICAL FUNCTION	p-value	Protein Mean Peak Intensity					Log_2FC ISO CT/CT
				Mean CT	Mean ISO CT	Mean FRDA	Median CT	Median ISO CT	
sp|Q9Y3B4|SF3B6_HUMAN	Splicing factor 3B subunit 6 OS=Homo sapiens OX=9606 GN=SF3B6 PE=1 SV=1	mRNA binding; RNA binding; mRNA splicing, via spliceosome	**0,009**	**16933,8**	**34646,0**	31681,3	16717,6	39571,4	1,03
sp|Q14119|VEZF1_HUMAN	Vascular endothelial zinc finger 1 OS=Homo sapiens OX=9606 GN=VEZF1 PE=1 SV=2	DNA-binding transcription activator activity, RNA polymerase II-specific; cellular defense response; endothelial cell development	**0,007**	**1866,0**	**3818,9**	3181,7	1519,7	3928,4	1,03
sp|Q12996|CSTF3_HUMAN	Cleavage stimulation factor subunit 3 OS=Homo sapiens OX=9606 GN=CSTF3 PE=1 SV=1	mRNA binding; mRNA splicing; mRNA cleavage	**0,004**	**14948,7**	**33377,1**	14846,0	15526,2	38310,5	1,16
sp|P46782|RS5_HUMAN	40S ribosomal protein S5 OS=Homo sapiens OX=9606 GN=RPS5 PE=1 SV=4	mRNA binding; structural constituent of ribosome; cytoplasmic translation; nuclear-transcribed mRNA catabolic process, nonsense-mediated decay	**0,009**	**115082,3**	**260217,7**	195136,9	125754,9	285223,7	1,18
sp|Q9Y333|LSM2_HUMAN	U6 snRNA-associated Sm-like protein LSm2 OS=Homo sapiens OX=9606 GN=LSM2 PE=1 SV=1	RNA binding; small GTPase binding; U6 snRNA binding; exonucleolytic catabolism of deadenylated mRNA; mRNA catabolic process; mRNA splicing, via spliceosome; spliceosomal tri-snRNP complex assembly	**0,000**	**16206,8**	**37123,1**	21222,2	14042,3	34627,0	1,20
sp|Q13895|BYST_HUMAN	Bystin OS=Homo sapiens OX=9606 GN=BYSL PE=1 SV=3	RNA binding; snoRNA binding; maturation of SSU-rRNA from tricistronic rRNA transcript (SSU-rRNA, 5.8S rRNA, LSU-rRNA); regulation of protein localization to nucleolus; ribosome biogenesis; rRNA processing	**0,010**	**3741,0**	**10966,5**	6589,5	3379,0	11715,0	1,55
sp|P55209|NP1L1_HUMAN	Nucleosome assembly protein 1-like 1 OS=Homo sapiens OX=9606 GN=NAP1L1 PE=1 SV=1	chromatin binding; histone binding; RNA binding; DNA replication; nucleosome assembly; positive regulation of cell population proliferation; positive regulation of neural precursor cell proliferation; positive regulation of neurogenesis	**0,002**	**87163,6**	**288853,7**	168816,9	91911,8	285655,0	1,73
sp|Q9H0U6|RM18_HUMAN	39S ribosomal protein L18, mitochondrial OS=Homo sapiens OX=9606 GN=MRPL18 PE=1 SV=1	structural constituent of ribosome; mitochondrial translational elongation; mitochondrial translational termination; rRNA import into mitochondrion	**0,003**	**5059,9**	**17288,0**	12073,3	4338,3	16813,5	1,77
sp|Q9UPT8|ZC3H4_HUMAN	Zinc finger CCCH domain-containing protein 4 OS=Homo sapiens OX=9606 GN=ZC3H4 PE=1 SV=3	DNA-binding transcription factor activity, RNA polymerase II-specific; metal ion binding; RNA binding	**0,003**	**1752,3**	**7219,2**	1992,1	1184,0	9209,7	2,04
sp|Q9BPX3|CND3_HUMAN	Condensin complex subunit 3 OS=Homo sapiens OX=9606 GN=NCAPG PE=1 SV=1	cell division; mitotic chromosome condensation	**0,010**	**5220,9**	**25872,6**	12065,7	6048,9	26157,9	2,31
sp|Q6PD62|CTR9_HUMAN	RNA polymerase-associated protein CTR9 homolog OS=Homo sapiens OX=9606 GN=CTR9 PE=1 SV=1	RNA polymerase II complex binding; SH2 domain binding; histone H2B ubiquitination; histone H3-K4 trimethylation; histone monoubiquitination; negative regulation of mRNA polyadenylation; negative regulation of transcription by RNA polymerase II; positive regulation of histone H2B ubiquitination; positive regulation of histone H3-K4 methylation; positive regulation of histone H3-K79 methylation; regulation of transcription, DNA-templated; stem cell population maintenance; transcription by RNA polymerase II; Wnt signaling pathway	**0,006**	**2959,0**	**14734,8**	7027,5	2451,6	16092,6	2,32
sp|Q16540|RM23_HUMAN	39S ribosomal protein L23, mitochondrial OS=Homo sapiens OX=9606 GN=MRPL23 PE=1 SV=1	RNA binding; structural constituent of ribosome; mitochondrial translation; mitochondrial translational elongation; mitochondrial translational termination	**0,008**	**1338,5**	**9098,9**	6535,8	1035,0	6856,3	2,77
sp|Q9NQ48-3|LZTL1_HUMAN	Isoform 3 of Leucine zipper transcription factor-like protein 1 OS=Homo sapiens OX=9606 GN=LZTFL1	identical protein binding; protein-containing complex binding; cilium assembly; negative regulation of protein localization to ciliary membrane; may play a role in neurite outgrowth	**0,003**	**1836,8**	**12707,1**	7955,7	1863,4	15702,5	2,79
sp|P55039|DRG2_HUMAN	Developmentally-regulated GTP-binding protein 2 OS=Homo sapiens OX=9606 GN=DRG2 PE=1 SV=1	GTPase activity; GTP binding; metal ion binding; RNA binding; signal transduction; cytoplasmic translation	**0,002**	**20701,8**	**41598,2**	25918,6	21229,7	38004,0	1,01
sp|P42167|LAP2B_HUMAN	Lamina-associated polypeptide 2, isoforms beta/gamma OS=Homo sapiens OX=9606 GN=TMPO PE=1 SV=2	cadherin binding; DNA binding; lamin binding; regulation of transcription, DNA-templated	**0,005**	**63865,6**	**132618,8**	82154,4	69386,3	151657,4	1,05
sp|Q86TG7-2|PEG10_HUMAN	Isoform 2 of Retrotransposon-derived protein PEG10 OS=Homo sapiens OX=9606 GN=PEG10	DNA binding; RNA binding; zinc binding; apoptotic process; cell differentiation; negative regulation of transforming growth factor beta receptor signaling pathway	**0,009**	**6148,6**	**15058,4**	8272,1	4703,3	13574,0	1,29
sp|P35244|RFA3_HUMAN	Replication protein A 14 kDa subunit OS=Homo sapiens OX=9606 GN=RPA3 PE=1 SV=1	damaged DNA binding; single-stranded DNA binding; base-excision repair; base-excision repair, gap-filling; DNA damage response, detection of DNA damage; DNA replication; DNA replication initiation; double-strand break repair via homologous recombination; regulation of signal transduction by p53 class mediator; telomere maintenance	**0,007**	**19194,8**	**42012,3**	25628,1	19383,0	44564,9	1,13
sp|Q9Y5B9|SP16H_HUMAN	FACT complex subunit SPT16 OS=Homo sapiens OX=9606 GN=SUPT16H PE=1 SV=1	nucleosome binding; RNA binding; DNA repair; DNA replication; DNA replication-independent nucleosome organization; nucleosome disassembly; positive regulation of DNA-templated transcription, elongation; positive regulation of transcription elongation from RNA polymerase II promoter; regulation of signal transduction by p53 class mediator	**0,008**	**58300,9**	**142036,4**	84854,9	59834,9	119219,3	1,28
sp|A6NHR9|SMHD1_HUMAN	Structural maintenance of chromosomes flexible hinge domain-containing protein 1 OS=Homo sapiens OX=9606 GN=SMCHD1 PE=1 SV=2	ATPase activity; ATP binding; DNA binding; dosage compensation by inactivation of X chromosome; double-strand break repair; heterochromatin organization involved in chromatin silencing; inactivation of X chromosome by DNA methylation; inactivation of X chromosome by heterochromatin assembly; negative regulation of double-strand break repair via homologous recombination; positive regulation of DNA repair; positive regulation of double-strand break repair via nonhomologous end joining	**0,005**	**10049,4**	**23057,4**	13469,3	10017,8	24380,7	1,20
sp|P30084|ECHM_HUMAN	Enoyl-CoA hydratase, mitochondrial OS=Homo sapiens OX=9606 GN=ECHS1 PE=1 SV=4	branched-chain amino acid catabolic process; fatty acid beta-oxidation	**0,000**	**142273,3**	**290415,1**	279863,0	142049,3	294716,6	1,03
sp|Q709C8-4|VP13C_HUMAN	Isoform 4 of Vacuolar protein sorting-associated protein 13C OS=Homo sapiens OX=9606 GN=VPS13C	Golgi to endosome transport; mitochondrion organization; negative regulation of parkin-mediated stimulation of mitophagy in response to mitochondrial depolarization; necessary for proper mitochondrial function and maintenance of mitochondrial transmembrane potential	**0,008**	**3194,1**	**6658,3**	5478,2	2497,3	6467,4	1,06

sp|O95486|SC24A_HUMAN	Protein transport protein Sec24A OS=Homo sapiens OX=9606 GN=SEC24A PE=1 SV=2	COPII vesicle coating; endoplasmic reticulum to Golgi vesicle-mediated transport; intracellular protein transport	**0,008**	**12196,8**	**25741,8**	21727,3	12893,1	27058,1	1,08
sp|P61289-2|PSME3_HUMAN	Isoform 2 of Proteasome activator complex subunit 3 OS=Homo sapiens OX=9606 GN=PSME3	endopeptidase activator activity; anaphase-promoting complex-dependent catabolic process; apoptotic process; regulation of Wnt signaling; positive regulation of endopeptidase activity; post-translational protein modification; pre-replicative complex assembly; proteasome-mediated ubiquitin-dependent protein catabolic process; protein deubiquitination; protein polyubiquitination; regulation of cellular amino acid metabolic process; regulation of G1/S transition of mitotic cell cycle; transmembrane transport	**0,000**	**36081,7**	**78630,4**	60058,4	37450,0	77805,1	1,12
sp|Q9Y5Y2|NUBP2_HUMAN	Cytosolic Fe-S cluster assembly factor NUBP2 OS=Homo sapiens OX=9606 GN=NUBP2 PE=1 SV=1	4 iron, 4 sulfur cluster binding; ATPase activity; ATP binding; iron-sulfur cluster binding;metal ion binding; nucleotide binding; cell projection organization; iron-sulfur cluster assembly	**0,000**	**3785,6**	**8705,8**	4910,2	3698,2	9136,3	1,20
sp|Q8NFF5|FAD1_HUMAN	FAD synthase OS=Homo sapiens OX=9606 GN=FLAD1 PE=1 SV=1	ATP binding; FMN adenylyltransferase activity; FAD biosynthetic process; riboflavin metabolic process	**0,005**	**6221,1**	**14397,0**	11685,2	7286,2	15276,9	1,21
sp|Q5MNZ6|WIPI3_HUMAN	WD repeat domain phosphoinositide-interacting protein 3 OS=Homo sapiens OX=9606 GN=WDR45B PE=1 SV=2	phosphatidylinositol-3,5-bisphosphate binding; phosphatidylinositol-3-phosphate binding; TSC1-TSC2 complex binding; autophagosome assembly; autophagy of mitochondrion; autophagy of nucleus; cellular response to starvation; protein lipidation; protein localization to phagophore assembly site	**0,007**	**4137,0**	**9678,1**	7092,9	3501,1	10986,8	1,23
sp|Q9BXW7|HDHD5_HUMAN	Haloacid dehalogenase-like hydrolase domain-containing 5 OS=Homo sapiens OX=9606 GN=HDHD5 PE=1 SV=1	glycerophospholipid biosynthetic process	**0,001**	**11048,8**	**26605,3**	20260,1	10986,2	21798,5	1,27
sp|P49915|GUAA_HUMAN	GMP synthase [glutamine-hydrolyzing] OS=Homo sapiens OX=9606 GN=GMPS PE=1 SV=1	GMP synthase activity; ATP binding; glutamine metabolic process	**0,002**	**51049,9**	**124581,2**	66104,5	57323,3	102939,4	1,29
sp|P13807-2|GYS1_HUMAN	Isoform 2 of Glycogen [starch] synthase, muscle OS=Homo sapiens OX=9606 GN=GYS1	glycogen biosynthetic process; heart development	**0,004**	**12251,2**	**29912,4**	17632,9	11898,6	29817,2	1,29
sp|O15260|SURF4_HUMAN	Surfeit locus protein 4 OS=Homo sapiens OX=9606 GN=SURF4 PE=1 SV=3	Golgi organization; retrograde vesicle-mediated transport, Golgi to endoplasmic reticulum	**0,006**	**46752,8**	**115458,8**	88125,4	49770,1	133919,2	1,30
sp|Q9ULS5|TMCC3_HUMAN	Transmembrane and coiled-coil domain protein 3 OS=Homo sapiens OX=9606 GN=TMCC3 PE=1 SV=3	14-3-3 protein binding; identical protein binding (Endoplasmic reticulum)	**0,004**	**2366,2**	**6022,3**	4093,0	1593,9	6012,2	1,35
sp|Q9NXE4-3|NSMA3_HUMAN	Isoform 3 of Sphingomyelin phosphodiesterase 4 OS=Homo sapiens OX=9606 GN=SMPD4	metal ion binding; sphingomyelin phosphodiesterase activity; endoplasmic reticulum organization; glycerophospholipid catabolic process	**0,006**	**1828,5**	**4753,5**	3687,2	1615,8	3958,1	1,38
sp|O60725|ICMT_HUMAN	Protein-S-isoprenylcysteine O-methyltransferase OS=Homo sapiens OX=9606 GN=ICMT PE=1 SV=1	cellular protein modification process; C-terminal protein methylation; MAPK cascade; post-translational protein modification; protein targeting to membrane	**0,005**	**2297,6**	**5974,0**	4904,6	2073,7	6358,6	1,38
sp|Q9Y6A9|SPCS1_HUMAN	Signal peptidase complex subunit 1 OS=Homo sapiens OX=9606 GN=SPCS1 PE=1 SV=4	peptidase activity; ribosome binding; protein targeting to ER; proteolysis; signal peptide processing	**0,002**	**3672,7**	**9710,4**	9448,5	1874,7	9472,9	1,40
sp|Q6YP21|KAT3_HUMAN	Kynurenine--oxoglutarate transaminase 3 OS=Homo sapiens OX=9606 GN=KYAT3 PE=1 SV=1	2-oxoglutarate metabolic process; biosynthetic process; cellular amino acid metabolic process; kynurenine metabolic process; L-kynurenine catabolic process	**0,008**	**5112,8**	**14240,7**	10108,1	5867,1	15612,3	1,48
sp|Q12770-4|SCAP_HUMAN	Isoform 4 of Sterol regulatory element-binding protein cleavage-activating protein OS=Homo sapiens OX=9606 GN=SCAP	sterol binding; unfolded protein binding; aging; cellular lipid metabolic process; cholesterol metabolic process; COPII-coated vesicle cargo loading; regulation of fatty acid biosynthetic process; response to hypoxia;<br>response to insulin; SREBP signaling pathway	**0,003**	**1609,9**	**4676,5**	3651,0	1680,9	5473,3	1,54
sp|Q6NUM9|RETST_HUMAN	All-trans-retinol 13,14-reductase OS=Homo sapiens OX=9606 GN=RETSAT PE=1 SV=2	saturation of all-trans-retinol to all-trans-13,14-dihydroretinol	**0,007**	**3047,6**	**9164,2**	6747,3	2858,6	10129,3	1,59
sp|Q70UQ0|IKIP_HUMAN	Inhibitor of nuclear factor kappa-B kinase-interacting protein OS=Homo sapiens OX=9606 GN=IKBIP PE=1 SV=1		**0,008**	**2866,4**	**9035,8**	4910,2	2727,2	10587,8	1,66
sp|O14618|CCS_HUMAN	Copper chaperone for superoxide dismutase OS=Homo sapiens OX=9606 GN=CCS PE=1 SV=1	cadherin binding; copper ion binding; cellular response to oxidative stress; positive regulation of oxidoreductase activity; protein maturation by copper ion transfer; removal of superoxide radicals; superoxide metabolic process	**0,003**	**2894,2**	**9613,7**	5014,0	1340,9	9058,7	1,73
sp|P19525|E2AK2_HUMAN	Interferon-induced, double-stranded RNA-activated protein kinase OS=Homo sapiens OX=9606 GN=EIF2AK2 PE=1 SV=2	ATP binding; double-stranded RNA binding; eukaryotic translation initiation factor 2alpha kinase activity; identical protein binding; non-membrane spanning protein tyrosine kinase activity; protein kinase activity; protein serine kinase activity; protein threonine kinase activity; transmembrane receptor protein tyrosine kinase activity; activation of MAPKK activity; cellular response to amino acid starvation; defense response to virus; endoplasmic reticulum unfolded protein response; innate immune response; negative regulation of apoptotic process; negative regulation of osteoblast proliferation; negative regulation of translation; negative regulation of viral genome replication; positive regulation of apoptotic process; positive regulation of chemokine production; positive regulation of cytokine production; positive regulation of NF-kappaB transcription factor activity; positive regulation of NIK/NF-kappaB signaling; positive regulation of stress-activated MAPK cascade; protein autophosphorylation; protein phosphorylation; regulation of hematopoietic progenitor cell differentiation; regulation of hematopoietic stem cell differentiation; regulation of hematopoietic stem cell proliferation; regulation of NLRP3 inflammasome complex assembly; response to interferon-alpha; response to lipopolysaccharide; response to toxic substance; response to virus; response to vitamin E; translation	**0,008**	**3072,5**	**11814,6**	11122,9	2972,5	10882,5	1,94
sp|Q6P1X6|CH082_HUMAN	UPF0598 protein C8orf82 OS=Homo sapiens OX=9606 GN=C8orf82 PE=1 SV=2		**0,004**	**1452,9**	**7950,9**	4706,9	977,1	10441,7	2,45
sp|Q8TF05-2|PP4R1_HUMAN	Isoform 2 of Serine/threonine-protein phosphatase 4 regulatory subunit 1 OS=Homo sapiens OX=9606 GN=PPP4R1	protein dephosphorylation; protein phosphorylation; signal transduction	**0,001**	**772,1**	**5013,9**	2202,2	554,6	5676,0	2,70

sp|Q8NCE2|MTMRE_HUMAN	Myotubularin-related protein 14 OS=Homo sapiens OX=9606 GN=MTMR14 PE=1 SV=2	phosphatidylinositol-3-phosphatase activity; protein tyrosine phosphatase activity; macroautophagy; phosphatidylinositol biosynthetic process	**0,001**	**1190,4**	**3264,9**	2363,0	1063,7	3113,3	1,46
sp|O15540-2|FABP7_HUMAN	Isoform 2 of Fatty acid-binding protein, brain OS=Homo sapiens OX=9606 GN=FABP7	lipid binding; epithelial cell proliferation; negative regulation of cell population proliferation; nervous system development; triglyceride catabolic process	**0,004**	**1412,6**	**3898,4**	2191,3	1063,7	4279,7	1,46
sp|Q92643|GPI8_HUMAN	GPI-anchor transamidase OS=Homo sapiens OX=9606 GN=PIGK PE=1 SV=2	attachment of GPI anchor to protein; protein localization to cell surface	**0,004**	**7850,3**	**16234,2**	12577,3	7826,5	18328,4	1,05
sp|Q8WUW1-2|BRK1_HUMAN	Isoform 2 of Protein BRICK1 OS=Homo sapiens OX=9606 GN=BRK1	protein binding; actin filament organization; cell motility; positive regulation of Arp2/3 complex-mediated actin nucleation; Rac protein signal transduction; regulation of actin polymerization or depolymerization	**0,005**	**16534,1**	**39471,7**	36667,9	17642,2	40625,2	1,26
sp|Q5VWJ9|SNX30_HUMAN	Sorting nexin-30 OS=Homo sapiens OX=9606 GN=SNX30 PE=1 SV=1	Phosphatidylinositol binding; protein transport; intracellular trafficking	**0,000**	**6938,5**	**17019,6**	18207,1	7531,6	16649,3	1,29
sp|Q96GZ6-9|S41A3_HUMAN	Isoform 9 of Solute carrier family 41 member 3 OS=Homo sapiens OX=9606 GN=SLC41A3	cation transmembrane transporter activity	**0,003**	**2616,0**	**6919,2**	4617,7	2581,4	7174,6	1,40
sp|Q14728|MFS10_HUMAN	Major facilitator superfamily domain-containing protein 10 OS=Homo sapiens OX=9606 GN=MFSD10 PE=1 SV=1	organic anion transmembrane transporter activity; tetracycline transmembrane transporter activity; apoptotic process; sodium-independent organic anion transport	**0,007**	**5507,8**	**15339,6**	14750,7	6755,2	16007,8	1,48
sp|Q9P0S9|TM14C_HUMAN	Transmembrane protein 14C OS=Homo sapiens OX=9606 GN=TMEM14C PE=1 SV=1	erythrocyte differentiation; heme biosynthetic process; mitochondrial transport; regulation of heme biosynthetic process	**0,009**	**2950,1**	**12698,8**	7440,0	3196,0	14938,4	2,11

DOWNREGULATED

**PROTEIN**	**GROUP AND RELATED GENE (GN)**	**GO: BIOLOGICAL FUNCTION**	**p-value**	**Protein Mean Peak Intensity**					**Log_2FC ISO CT/CT**
				**Mean CT**	**Mean ISO CT**	**Mean FRDA**	**Median CT**	**Median ISO CT**	
sp|Q02539|H11_HUMAN	Histone H1.1 OS=Homo sapiens OX=9606 GN=HIST1H1A PE=1 SV=3	chromosome condensation; negative regulation of chromatin silencing; negative regulation of DNA recombination; chromatin binding; double-stranded DNA binding	**0,000**	**162206,4**	**7956,5**	14742,2	152890,7	7701,6	-4,35
sp|P40425|PBX2_HUMAN	Pre-B-cell leukemia transcription factor 2 OS=Homo sapiens OX=9606 GN=PBX2 PE=1 SV=2	chromatin binding; DNA-binding transcription activator activity, RNA polymerase II-specific; transcription factor binding; brain development; embryonic limb morphogenesis; embryonic organ development; neuron development; proximal/distal pattern formation; regulation of transcription by RNA polymerase II	**0,006**	**13857,9**	**6746,6**	9320,9	13178,8	5136,5	-1,04
sp|Q7Z5J4|RAI1_HUMAN	Retinoic acid-induced protein 1 OS=Homo sapiens OX=9606 GN=RAI1 PE=1 SV=2	metal ion binding; circadian regulation of gene expression; negative regulation of multicellular organism growth; positive regulation of transcription, DNA-templated; regulation of transcription by RNA polymerase II; skeletal system development	**0,000**	**5210,9**	**1722,1**	3389,7	5262,5	1525,6	-1,60
sp|Q59GN2|R39L5_HUMAN	Putative 60S ribosomal protein L39-like 5 OS=Homo sapiens OX=9606 GN=RPL39P5 PE=5 SV=2	stuctural constituent of ribosome; translation	**0,009**	**52098,5**	**21273,6**	29642,0	44741,9	22690,5	-1,29
sp|P80723|BASP1_HUMAN	Brain acid soluble protein 1 OS=Homo sapiens OX=9606 GN=BASP1 PE=1 SV=2	protein domain specific binding; transcription corepressor activity;transcription regulatory region sequence-specific DNA binding; mesenchymal to epithelial transition; negative regulation of transcription, DNA-templated; positive regulation of heart growth; substantia nigra development; thorax and anterior abdomen determination; regulate actin dynamics and presynaptic vesicle cycling at axon terminals, thereby facilitating axonal growth, regeneration, and plasticity	**0,000**	**1687939,3**	**809983,4**	1252682,1	1646542,2	743255,5	-1,06
sp|Q8N111|CEND_HUMAN	Cell cycle exit and neuronal differentiation protein 1 OS=Homo sapiens OX=9606 GN=CEND1 PE=1 SV=1	adult walking behavior; cerebellar granular layer maturation; cerebellar Purkinje cell differentiation; negative regulation of cerebellar granule cell precursor proliferation; radial glia guided migration of cerebellar granule cell	**0,003**	**43229,6**	**19533,9**	34504,5	42246,7	12176,8	-1,15
sp|Q5RI15|COX20_HUMAN	Cytochrome c oxidase assembly protein COX20, mitochondrial OS=Homo sapiens OX=9606 GN=COX20 PE=1 SV=2	mitochondrial cytochrome c oxidase assembly	**0,010**	**10517,7**	**2947,2**	10087,9	8609,5	1673,7	-1,84
sp|Q8WVI7|PPR1C_HUMAN	Protein phosphatase 1 regulatory subunit 1C OS=Homo sapiens OX=9606 GN=PPP1R1C PE=3 SV=1	protein phosphatase inhibitor activity; intracellular signal transduction; cell cycle; cell division	**0,001**	**43879,7**	**14664,0**	39211,4	43812,9	10588,7	-1,58
sp|Q15125|EBP_HUMAN	3-beta-hydroxysteroid-Delta(8),Delta(7)-isomerase OS=Homo sapiens OX=9606 GN=EBP PE=1 SV=3	cholesterol biosynthetic process; cholesterol metabolic process; skeletal system development	**0,000**	**13202,2**	**4857,6**	11475,3	12039,3	4735,5	-1,44
sp|P56181|NDUV3_HUMAN	NADH dehydrogenase [ubiquinone] flavoprotein 3, mitochondrial OS=Homo sapiens OX=9606 GN=NDUFV3 PE=1 SV=2	mitochondrial ATP synthesis coupled electron transport; mitochondrial electron transport, NADH to ubiquinone; mitochondrial respiratory chain complex I assembly	**0,001**	**21572,7**	**8248,4**	16136,6	19767,9	9730,8	-1,39
sp|Q13555-6|KCC2G_HUMAN	Isoform 6 of Calcium/calmodulin-dependent protein kinase type II subunit gamma OS=Homo sapiens OX=9606 GN=CAMK2G	nervous system development; regulation of calcium ion transport; regulation of neuron projection development; regulation of skeletal muscle adaptation	**0,005**	**24381,5**	**9449,8**	20862,8	29545,1	9497,4	-1,37
sp|P54803|GALC_HUMAN	Galactocerebrosidase OS=Homo sapiens OX=9606 GN=GALC PE=1 SV=3	galactosylceramide catabolic process; glycosphingolipid metabolic process; myelination	**0,000**	**20556,2**	**8195,8**	15326,0	19685,1	6534,5	-1,33
sp|P09382|LEG1_HUMAN	Galectin-1 OS=Homo sapiens OX=9606 GN=LGALS1 PE=1 SV=2	carbohydrate binding; identical protein binding; lactose binding; laminin binding; RNA binding apoptotic process; cellular protein metabolic process; cellular response to glucose stimulus; cellular response to organic cyclic compound; myoblast differentiation; negative regulation of cell-substrate adhesion; negative regulation of neuron projection development; plasma cell differentiation; positive regulation of erythrocyte aggregation; positive regulation of I-kappaB kinase/NF-kappaB signaling; positive	**0,002**	**42609,4**	**17767,9**	7614,2	43715,6	11256,9	-1,26

		regulation of viral entry into host cell; post-translational protein modification; regulation of apoptotic process; response to axon injury; response to drug ; response to isolation stress; T cell costimulation							
sp|O75781-2|PALM_HUMAN	Isoform 2 of Paralemmin-1 OS=Homo sapiens OX=9606 GN=PALM	adenylate cyclase-inhibiting G protein-coupled receptor signaling pathway; cellular response to electrical stimulus; cytoskeleton organization; synapse maturation	**0,000**	**148105,6**	**62681,7**	124981,8	142596,9	66614,7	-1,24
sp|P45954|ACDSB_HUMAN	Short/branched chain specific acyl-CoA dehydrogenase, mitochondrial OS=Homo sapiens OX=9606 GN=ACADSB PE=1 SV=1	branched-chain amino acid catabolic process; fatty acid metabolic process; isoleucine catabolic process	**0,004**	**1252,2**	**563,4**	873,1	1314,8	405,0	-1,15
sp|O75323|NIPS2_HUMAN	Protein NipSnap homolog 2 OS=Homo sapiens OX=9606 GN=NIPSNAP2 PE=1 SV=1	mitochondrion organization; oxidative phosphorylation; positive regulation of high voltage-gated calcium channel activity	**0,003**	**8121,5**	**3755,6**	4617,7	7638,6	3625,7	-1,11
sp|O75663|TIPRL_HUMAN	TIP41-like protein OS=Homo sapiens OX=9606 GN=TIPRL PE=1 SV=2	DNA damage checkpoint; negative regulation of phosphoprotein phosphatase activity; regulation of phosphoprotein phosphatase activity; TOR signaling	**0,004**	**63340,2**	**29390,8**	38318,2	57436,2	18339,7	-1,11
sp|Q96GY0|ZC21A_HUMAN	Zinc finger C2HC domain-containing protein 1A OS=Homo sapiens OX=9606 GN=ZC2HC1A PE=1 SV=2	metal ion binding	**0,010**	**105556,2**	**50530,2**	91155,5	127768,2	48731,4	-1,06
sp|O15069|NACAD_HUMAN	NAC-alpha domain-containing protein 1 OS=Homo sapiens OX=9606 GN=NACAD PE=1 SV=3	unfolded protein binding; protein targeting to membrane	**0,000**	**20376,3**	**9873,1**	15387,7	19560,1	11527,6	-1,05
sp|P05413|FABPH_HUMAN	Fatty acid-binding protein, heart OS=Homo sapiens OX=9606 GN=FABP3 PE=1 SV=4	cytoskeletal protein binding; icosatetraenoic acid binding; long-chain fatty acid binding; long-chain fatty acid transporter activity; oleic acid binding brown fat cell differentiation; cholesterol homeostasis; fatty acid metabolic process; intracellular lipid transport; long-chain fatty acid transport; negative regulation of cell population proliferation; phospholipid homeostasis; positive regulation of long-chain fatty acid import into cell; positive regulation of phospholipid biosynthetic process; regulation of fatty acid oxidation; regulation of phosphatidylcholine biosynthetic process; response to drug; response to fatty acid; response to insulin; triglyceride catabolic process	**0,001**	**35322,0**	**9372,6**	11533,1	33570,8	8549,3	-1,91
sp|O75056|SDC3_HUMAN	Syndecan-3 OS=Homo sapiens OX=9606 GN=SDC3 PE=1 SV=2	identical protein binding; cell migration; glycosaminoglycan biosynthetic process; glycosaminoglycan catabolic process; leukocyte migration; retinoid metabolic process; may have a role in the organization of cell shape by affecting the actin cytoskeleton, possibly by transferring signals from the cell surface in a sugar-dependent mechanism	**0,003**	**29250,0**	**6666,0**	11609,7	29752,7	2764,7	-2,13
sp|O75061-4|AUXI_HUMAN	Isoform 4 of Putative tyrosine-protein phosphatase auxilin OS=Homo sapiens OX=9606 GN=DNAJC6	clathrin binding; protein tyrosine phosphatase activity; clathrin coat disassembly; clathrin-dependent endocytosis; membrane organization; post-Golgi vesicle-mediated transport; regulation of clathrin-dependent endocytosis; synaptic vesicle uncoating	**0,003**	**46130,1**	**13027,2**	30196,2	42984,9	9906,2	-1,82
sp|P04271|S100B_HUMAN	Protein S100-B OS=Homo sapiens OX=9606 GN=S100B PE=1 SV=2	Calcium-dependent protein binding; calcium ion binding; identical protein binding; protein homodimerization activity; RAGE receptor binding; S100 protein binding; tau protein binding; zinc ion binding axonogenesis; central nervous system development; innate immune responsee; learning or memory; positive regulation of cell population proliferation; positive regulation of I-kappaB kinase/NF-kappaB signaling	**0,002**	**4858,3**	**1452,5**	1181,2	5091,4	1199,2	-1,74
sp|P05067-3|A4_HUMAN	Isoform L-APP677 of Amyloid-beta precursor protein OS=Homo sapiens OX=9606 GN=APP	acetylcholine receptor activator activity; chaperone; chemoattractant activity; chromatin binding; DNA binding; enzyme binding; enzyme binding; G protein-coupled receptor binding; growth factor receptor binding; heparan sulfate binding; integrin binding; activation of MAPK activity; adult locomotory behavior; axo-dendritic transport; axon midline choice point recognition; axonogenesis; calcium-mediated signaling; cell adhesion; cellular copper ion homeostasis; cellular process; cellular protein metabolic process; cellular response to amyloid-beta; cellular response to cAMP; dendrite development; endocytosis; extracellular matrix organization; locomotory behavior; modulation of excitatory postsynaptic potential; negative regulation of gene expression; negative regulation of long-term synaptic potentiation; negative regulation of mitochondrion organization; negative regulation of neuron death; neuromuscular process controlling balance; neuron projection development; neuron projection maintenance; neuron remodeling; synapse organization; synaptic growth at neuromuscular junction	**0,002**	**79564,4**	**33268,8**	69069,6	71795,5	30510,9	-1,26
sp|P17677|NEUM_HUMAN	Neuromodulin OS=Homo sapiens OX=9606 GN=GAP43 PE=1 SV=1	calmodulin binding; phosphatidylinositol phosphate binding; phosphatidylserine binding; axon choice point recognition; axon regeneration; cell fate commitment; glial cell differentiation; protein kinase C-activating G protein-coupled receptor signaling pathway; regulation of filopodium assembly; regulation of growth; regulation of postsynaptic specialization assembly; response to wounding; tissue regeneration	**0,000**	**1235395,2**	**546870,5**	1034495,6	1253315,8	566231,3	-1,18
sp|Q02952-3|AKA12_HUMAN	Isoform 3 of A-kinase anchor protein 12 OS=Homo sapiens OX=9606 GN=AKAP12	adenylate cyclase binding; calmodulin binding; protein kinase A binding; modulation of chemical synaptic transmission; positive regulation of ERK1 and ERK2 cascade; response to electrical stimulus; response to lipopolysaccharide; positive regulation of oligodendrocyte apoptotic process	**0,000**	**98199,7**	**47148,5**	79823,6	99442,5	45654,0	-1,06
sp|O75351|VPS4B_HUMAN	Vacuolar protein sorting-associated protein 4B OS=Homo sapiens OX=9606 GN=VPS4B PE=1 SV=2	ATPase activity; ATP binding; identical protein binding; microtubule-severing ATPase activity; protein homodimerization activity	**0,001**	**16493,4**	**8083,9**	10462,6	17530,9	7044,3	-1,03
sp|O60636|TSN2_HUMAN	Tetraspanin-2 OS=Homo sapiens OX=9606 GN=TSPAN2 PE=1 SV=2	astrocyte development; axon development; brain development; inflammatory response; microglia development; myelination; oligodendrocyte differentiation	**0,009**	**1806,3**	**808,5**	1340,4	1759,7	759,1	-1,16
sp|Q13740|CD166_HUMAN	CD166 antigen OS=Homo sapiens OX=9606 GN=ALCAM PE=1 SV=2	protein binding; axon extension involved in axon guidance; cell adhesion; heterophilic cell-cell adhesion via plasma membrane cell adhesion molecules; neuron projection extension; signal transduction	**0,009**	**237315,0**	**53443,3**	144044,7	228995,8	53646,2	-2,15
sp|O00203-3|AP3B1_HUMAN	Isoform 2 of AP-3 complex subunit beta-1 OS=Homo sapiens OX=9606 GN=AP3B1	GTP-dependent protein binding; anterograde axonal transport; anterograde synaptic vesicle transport; establishment of protein localization to mitochondrial membrane involved in mitochondrial fission; mRNA transcription by RNA polymerase II	**0,000**	**233981,0**	**54492,8**	188646,5	228019,4	36883,8	-2,10
sp|Q9UGI8|TES_HUMAN	Testin OS=Homo sapiens OX=9606 GN=TES PE=1 SV=1	cadherin binding; RNA binding; negative regulation of cell population proliferation; may play a role in cell adhesion, cell spreading and in the reorganization of the actin cytoskeleton	**0,001**	**45960,6**	**10767,2**	40885,9	47701,8	9723,2	-2,09
sp|Q9NR46|SHLB2_HUMAN	Endophilin-B2 OS=Homo sapiens OX=9606 GN=SH3GLB2 PE=1 SV=1	Cadherin binding; identical protein binding	**0,001**	**29053,1**	**7041,0**	6385,6	26340,0	2752,1	-2,04

sp|P07196|NFL_HUMAN	Neurofilament light polypeptide OS=Homo sapiens OX=9606 GN=NEFL PE=1 SV=3	protein binding; phospholipase binding; structural constituent of cytoskeleton; structural constituent of postsynaptic intermediate filament cytoskeleton; anterograde axonal transport; axonal transport of mitochondrion; intermediate filament organization; intermediate filament polymerization or depolymerization; locomotion; MAPK cascade; microtubule cytoskeleton organization; negative regulation of neuron apoptotic process; neurofilament bundle assembly; neurofilament cytoskeleton organization; neuromuscular process controlling balance; neuron projection morphogenesis; peripheral nervous system axon regeneration; positive regulation of axonogenesis; protein polymerization; regulation of axon diameter; regulation of NMDA receptor activity; response to acrylamide; response to corticosterone; response to peptide hormone; response to sodium arsenite; response to toxic substance; retrograde axonal transport; spinal cord development; synapse maturation	**0,001**	**7321839,6**	**2300996,1**	4732646,2	6625586,3	1986276,0	-1,67
sp|O00151|PDLI1_HUMAN	PDZ and LIM domain protein 1 OS=Homo sapiens OX=9606 GN=PDLIM1 PE=1 SV=4	actin binding; cadherin binding involved in cell-cell adhesion; metal ion binding; muscle alpha-actinin binding; transcription coactivator activity; actin cytoskeleton organization; establishment or maintenance of actin cytoskeleton polarity; heart development; maintenance of cell polarity; muscle structure development; regulation of transcription by RNA polymerase II; response to hypoxia; response to oxidative stress; stress fiber assembly	**0,000**	**90040,9**	**30387,5**	50060,9	85171,0	21241,2	-1,57
sp|P09493-5|TPM1_HUMAN	Isoform 5 of Tropomyosin alpha-1 chain OS=Homo sapiens OX=9606 GN=TPM1	actin binding; actin filament binding ; cytoskeletal protein binding; identical protein binding; protein heterodimerization activity; protein homodimerization activity; structural constituent of cytoskeleton; structural constituent of muscle	**0,005**	**37645,8**	**13192,8**	22935,8	35785,7	14387,8	-1,51
sp|Q9UM54-6|MYO6_HUMAN	Isoform 6 of Unconventional myosin-VI OS=Homo sapiens OX=9606 GN=MYO6	actin binding; actin-dependent ATPase activity; actin filament binding; ADP binding; ATP binding; calmodulin binding; microfilament motor activity; actin filament-based movement; actin filament organization; DNA damage response, signal transduction by p53 class mediator; endocytosis; vesicle transport along actin filament	**0,001**	**19060,2**	**7091,1**	12330,3	17728,2	6665,2	-1,43
sp|Q9HCM2|PLXA4_HUMAN	Plexin-A4 OS=Homo sapiens OX=9606 GN=PLXNA4 PE=1 SV=4	semaphorin receptor activity; negative regulation of cell adhesion; positive regulation of axonogenesis; regulation of axon extension involved in axon guidance; regulation of cell migration; regulation of cell shape; semaphorin-plexin signaling pathway involved in axon guidance	**0,005**	**64963,2**	**25731,0**	62063,4	56792,2	29293,1	-1,34
sp|Q9UHC6|CNTP2_HUMAN	Contactin-associated protein-like 2 OS=Homo sapiens OX=9606 GN=CNTNAP2 PE=1 SV=1	enzyme binding; brain development; cell adhesion; clustering of voltage-gated potassium channels; learning; limbic system development; neuron projection development; neuron projection morphogenesis; neuron recognition; positive regulation of gap junction assembly; protein localization to juxtaparanode region of axon; transmission of nerve impulse	**0,004**	**35970,4**	**14632,0**	27178,2	35855,8	13537,0	-1,30
sp|O15394|NCAM2_HUMAN	Neural cell adhesion molecule 2 OS=Homo sapiens OX=9606 GN=NCAM2 PE=1 SV=2	identical protein binding; axonal fasciculation; neuron cell-cell adhesion	**0,000**	**67221,9**	**27533,9**	85653,3	72958,3	25818,9	-1,29
sp|Q7Z2K8|GRIN1_HUMAN	G protein-regulated inducer of neurite outgrowth 1 OS=Homo sapiens OX=9606 GN=GPRIN1 PE=1 SV=2	phosphoprotein binding; neuron projection development	**0,000**	**104499,0**	**45276,8**	74697,8	104184,8	35785,5	-1,21
sp|O15143|ARC1B_HUMAN	Actin-related protein 2/3 complex subunit 1B OS=Homo sapiens OX=9606 GN=ARPC1B PE=1 SV=3	Arp2/3 complex-mediated actin nucleation; ephrin receptor signaling pathway; Fc-gamma receptor signaling pathway involved in phagocytosis; response to estradiol; response to estrogen; actin binding; structural constituent of cytoskeleton	**0,003**	**19370,0**	**9225,2**	5806,3	17823,9	7009,7	-1,07
sp|P04216|THY1_HUMAN	Thy-1 membrane glycoprotein OS=Homo sapiens OX=9606 GN=THY1 PE=1 SV=2	GPI anchor binding; GTPase activator activity; integrin binding; protein kinase binding; cell-cell adhesion; cell-cell signaling; cytoskeleton organization; focal adhesion assembly; integrin-mediated signaling pathway; regulation of axonogenesis; negative regulation of neuron projection regeneration; positive regulation of focal adhesion assembly; positive regulation of heterotypic cell-cell adhesion ; positive regulation of release of sequestered calcium ion into cytosol; regulation of cell-matrix adhesion	**0,000**	**136683,3**	**65449,2**	131625,4	129246,4	62074,0	-1,06
sp|Q92563|TICN2_HUMAN	Testican-2 OS=Homo sapiens OX=9606 GN=SPOCK2 PE=1 SV=1	calcium ion binding; extracellular matrix binding; glycosaminoglycan binding; metalloendopeptidase inhibitor activity; extracellular matrix organization; positive regulation of cell motility; positive regulation of cell-substrate adhesion; regulation of cell differentiation; synapse assembly	**0,010**	**15479,4**	**7427,4**	9968,4	14369,5	7028,8	-1,06
sp|P02545|LMNA_HUMAN	Prelamin-A/C OS=Homo sapiens OX=9606 GN=LMNA PE=1 SV=1	identical protein binding; structural molecule activity; cellular protein localization; cellular response to hypoxia; DNA double-strand break attachment to nuclear envelope; establishment or maintenance of microtubule; cytoskeleton polarity; IRE1-mediated unfolded protein response; mitotic nuclear envelope reassembly; muscle organ development; negative regulation of cardiac muscle hypertrophy in response to stress; negative regulation of cell population proliferation; nuclear envelope organization; positive regulation of cell aging; protein localization to nucleus; regulation of cell migration; regulation of telomere maintenance; nuclear assembly, chromatin organization, nuclear membrane and telomere dynamics; required for normal development of peripheral nervous system and skeletal muscle and for muscle satellite cell proliferation	**0,001**	**50695,3**	**24589,4**	24063,7	46463,5	24973,7	-1,04
sp|Q9Y639|NPTN_HUMAN	Neuroplastin OS=Homo sapiens OX=9606 GN=NPTN PE=1 SV=2	cell adhesion molecule binding; cell-cell adhesion mediator activity; ion channel binding; axon guidance; cellular calcium ion homeostasis; dendrite self-avoidance; excitatory synapse assembly; homophilic cell adhesion via plasma membrane adhesion molecules; long-term synaptic potentiation; negative regulation of cytokine production; positive regulation of cellular protein localization; positive regulation of cytosolic calcium ion concentration; positive regulation of ERK1 and ERK2 cascade; positive regulation of fibroblast growth factor receptor signaling pathway; positive regulation of long-term neuronal synaptic plasticity; positive regulation of long-term synaptic potentiation; positive regulation of neuron projection development; positive regulation of protein phosphorylation; regulation of receptor localization to synapse; trans-synaptic signaling by trans-synaptic complex, modulating synaptic transmission	**0,000**	**134619,3**	**65472,8**	91972,8	134484,9	59869,0	-1,04
sp|P32004|L1CAM_HUMAN	Neural cell adhesion molecule L1 OS=Homo sapiens OX=9606 GN=L1CAM PE=1 SV=2	axon guidance receptor activity; protein domain specific binding; axon development; axon guidance; cell adhesion; cell-matrix adhesion; chemotaxis; homophilic cell adhesion via plasma membrane adhesion molecules; nervous system development; neuron projection development; positive regulation of axon extension; synapse organization	**0,000**	**394117,0**	**192357,9**	342555,6	371148,3	167235,0	-1,03
sp|Q92572|AP3S1_HUMAN	AP-3 complex subunit sigma-1 OS=Homo sapiens OX=9606 GN=AP3S1 PE=1 SV=1	anterograde axonal transport; anterograde synaptic vesicle transport; insulin receptor signaling pathway; intracellular protein transport; vesicle-mediated transport	**0,007**	**14932,6**	**7338,2**	10737,1	14273,4	8550,7	-1,02
sp|P13987|CD59_HUMAN	CD59 glycoprotein OS=Homo sapiens OX=9606 GN=CD59 PE=1 SV=1	complement binding; cell surface receptor signaling pathway; endoplasmic reticulum to Golgi vesicle-mediated transport	**0,001**	**43373,9**	**20512,1**	42075,7	41121,0	14890,7	-1,08
sp|O95297|MPZL1_HUMAN	Myelin protein zero-like protein 1 OS=Homo sapiens OX=9606 GN=MPZL1 PE=1 SV=1	structural molecule activity; cell-cell signaling; transmembrane receptor protein tyrosine kinase signaling pathway	**0,002**	**31014,3**	**15436,6**	26992,5	26668,5	14530,2	-1,01
sp|O43761|SNG3_HUMAN	Synaptogyrin-3 OS=Homo sapiens OX=9606 GN=SYNGR3 PE=1 SV=2	positive regulation of transporter activity; regulated exocytosis	**0,001**	**2530,7**	**752,7**	2269,3	2330,9	800,3	-1,75

sp|Q4VC31|CCD58_HUMAN	Coiled-coil domain-containing protein 58 OS=Homo sapiens OX=9606 GN=CCDC58 PE=1 SV=1	Mitochondrial Matrix Import Factor 23	**0,001**	**24128,2**	**9611,4**	13886,8	21924,3	8024,0	-1,33
sp|O60641|AP180_HUMAN	Clathrin coat assembly protein AP180 OS=Homo sapiens OX=9606 GN=SNAP91 PE=1 SV=2	1-phosphatidylinositol binding; clathrin heavy chain binding; clathrin coat assembly; clathrin-dependent endocytosis; protein transport; regulation of clathrin-dependent endocytosis; synaptic vesicle budding from presynaptic endocytic zone membrane; vesicle budding from membrane	**0,006**	**55052,5**	**21985,7**	52741,6	60405,0	19703,7	-1,32
sp|Q13433|S39A6_HUMAN	Zinc transporter ZIP6 OS=Homo sapiens OX=9606 GN=SLC39A6 PE=1 SV=3	amino acid transmembrane transport; glutamine transport; sodium ion transport	**0,005**	**7822,4**	**3180,8**	6332,6	6754,3	3005,9	-1,30
sp|Q8TAC9|SCAM5_HUMAN	Secretory carrier-associated membrane protein 5 OS=Homo sapiens OX=9606 GN=SCAMP5 PE=1 SV=1	protein-containing complex binding; exocytosis; negative regulation of endocytosis; positive regulation of calcium ion-dependent exocytosis; positive regulation of cytokine production; protein transport; response to endoplasmic reticulum stress	**0,001**	**29310,3**	**13752,6**	22589,0	28484,3	10957,2	-1,09
sp|Q8TC26|TM163_HUMAN	Transmembrane protein 163 OS=Homo sapiens OX=9606 GN=TMEM163 PE=2 SV=1	zinc ion import into synaptic vesicle	**0,000**	**38717,6**	**18369,4**	27010,1	36727,5	15172,3	-1,08
